# Changes in prevalence of alcohol and tobacco consumption across districts of India, 2016 and 2021

**DOI:** 10.1186/s12889-025-23029-z

**Published:** 2025-05-27

**Authors:** Sunil Rajpal, Abhishek Kumar, Shreya Ronanki, Nehantha Sathesh, Rockli Kim, S. V. Subramanian

**Affiliations:** 1 Department of Economics, FLAME University, Pune, India; 2 Centre for Research in Wellbeing and Happiness, FLAME University, Pune, India; 3 Division of Health Policy and Management, College of Health Science, Korea University, 145 Anam-Ro, Seongbuk-Gu, Seoul, 02841 South Korea; 4 Harvard Center for Population and Development Studies, Cambridge, MA US; 5 Department of Social and Behavioral Sciences, Harvard T.H. Chan School of Public Health, Boston, MA US

**Keywords:** Alcohol, Tobacco, India, District-level consumption, Risk factors

## Abstract

**Background:**

India witnessed a rise in alcohol and tobacco consumption in the last few decades. However, the burden varies because of the huge population, diverse socioeconomic, cultural, and geographical characteristics, and different liquor policies across states. To understand the burden and progress, it is crucial to examine the consumption patterns at smaller geographical units. This study examines the trends and patterns in alcohol and tobacco consumption across 720 districts nested within 36 states (Union Territories) between 2016 and 2021.

**Methods:**

We used the fourth (2015–16) and fifth (2019–21) rounds of the National Family Health Survey of India. Both rounds provide the district-representative sample for the estimation. We used a 4-level (level 1-individuals; level 2-clusters; level 3-districts; level 4-states) random effects model to compute the predicted probabilities of alcohol and tobacco consumption (among males and females) for 720 districts in India. We used decile positions to map the consumption prevalence across districts.

**Results:**

Between 2016 and 2021, alcohol and tobacco consumption among men in India declined significantly, with national alcohol usage dropping from 29.2% to 17.5% and tobacco from 44.5% to 32.6%. The variation attributable to states for both alcohol (40.5% in 2016 and 56.6% in 2021) and tobacco (58% in 2016 and 68.3% in 2021) consumption among men was higher in 2021 as compared to 2016. The consumption of both tobacco and alcohol was notably high in the north-eastern states among both men and women. More than 80% of the districts reported a moderate to high reduction in alcohol consumption for men between the two rounds.

**Conclusions:**

The national decline in tobacco and alcohol consumption indicates progress. However, there remains a need for continuous and targeted interventions to target high-consumption pockets and address geographic disparities. The results of the present study indicate that interventions focusing on changing tobacco and alcohol consumption should consider the geographical variation at smaller administrative units. By implementing evidence-based policies and interventions suited to the needs of the local areas, public health authorities can continue to make significant strides in improving the health and well-being of the population and reducing the burden of alcohol and tobacco-related diseases.

**Supplementary Information:**

The online version contains supplementary material available at 10.1186/s12889-025-23029-z.

## Background

Over four decades ago, the 32nd World Health Assembly took explicit cognizance of the problems related to excessive consumption of alcohol and tobacco and identified it as a major public health problem [1]. Findings from recent studies have observed that any amount of alcohol and tobacco consumption is significantly associated with an increased likelihood of chronic ailments (mainly cancer and heart diseases) [2, 3]. A large proportion of diseases and deaths in South Asia are associated with problematic patterns of alcohol and tobacco consumption (smoked and smokeless) [4]. The consumption patterns are complex to understand in developing countries like India due to its huge population base and diverse characteristics across population sub-groups based on geography, social environment (deprivation, social capital, and income inequality), and cultural factors [5, 6]. For example, alcohol consumption in India varies considerably across religions and social castes. India’s alcohol and tobacco policies and administrative regulations have historically varied across states with limited national-level coordination [5, 7]. During 2016–2021, several states such as Tamil Nadu and Haryana introduced policies to restrict consumption but were forced to revoke them due to fiscal concerns and increased illicit alcohol use and distribution [7]. Further, geographical administrative units in India are categorized into three layers: states, districts, and villages. While policies and regulations are designed at the state level (based on the burden across states), they can mask the variations between districts within states. Studies have found significant inter-district and intra-district variations within the larger geographies for several outcomes like child undernutrition, child vaccination, and multidimensional poverty [8–10]. Examining the overtime trends and patterns in consumption for smaller administrative units (districts) can offer a better and more comprehensive understanding of the burden.

Interestingly, alcohol and tobacco use among females has increased in India, and future generations are anticipated to experience a relatively higher burden of non-communicable diseases (NCDs) among females [11–13]. The situation can be alarming, keeping in mind the risks pertaining to its effects on maternal and child health-related consequences [14]. Across geographies, regional variations have been observed in alcohol consumption in India, with the highest burden in the states of North-east, Chhattisgarh, Telangana, Himachal Pradesh, Punjab, and Jharkhand [15]. Further, demographic patterning shows that the age at initiation of alcohol and tobacco consumption is decreasing, with more and more younger groups being exposed to these risk factors [16]. Furthermore, younger age groups are found to be more likely to consume multiple substances (alcohol and tobacco), and hence are more prone to increased risk of hypertension and other chronic ailments, including mental health issues, in India [17].

Evidence from previous studies points towards a limited success of alcohol control policies and programs, in terms of reducing the consumption burden and its associated impact in India [18]. While policy targeting (and administrative focus) in the case of alcohol and tobacco consumption is mostly state-oriented, it is critical to understand the intricacies at the district level. Examining the burden and overtime changes in consumption patterns can offer valuable insights to enhance the policy targeting strategies.

Further, it is imperative from the policy standpoint to assess the burden of multiple substance consumption (alcohol and tobacco) to prioritize the high-burden spots at smaller geographies. This paper utilized the two rounds of nationally representative cross-sectional surveys to analyze the district-level patterns in alcohol and tobacco consumption among men and women in India. Further, we also examined the change in consumption patterns across 720 districts between 2016 and 2021.

## Methods

### Overview

The study utilized the two rounds of the cross-sectional National Family Health Survey (NFHS) conducted in 2015–16 and 2019–21. Initiated in the early 1990s, the NFHS in India provides nationally representative information on key indicators such as fertility and mortality rates, contraceptive use, immunization coverage, and nutritional status of children, women, and men, making it a critical tool for assessing India’s health and demographic trends.

### Data and survey design

The NFHS adopts a multistage, stratified cluster sampling design. Individual-level data from the fourth (2015–16) and fifth (2019–21) rounds of the surveys are the latest available and were used in this study. The NFHS collects the data for rural and urban areas separately using the latest census data as the sampling frame. According to the Demographic and Health Surveys (DHS), the clusters, which are villages (for rural areas) and Census Enumeration Blocks (CEBs) (for urban areas), serve as primary sampling units (PSUs). A representative sample of households was constructed for rural areas via stratified, probabilistic two-stage random sampling. In the first stage, the PSU (or cluster) corresponding to villages was classified on key variables including social group (percentage of population belonging to scheduled castes/scheduled tribes (SC/ST) and education (literacy rate of women aged 6 + years), and was selected by probability proportional to cluster size. This was followed by household selections from the household list using systematic sampling with equal probability [19, 20]. A similar process was used for the urban areas, but because the urban clusters correspond to CEBs, a mix of a two-stage sampling approach was employed. It may be noted that PSUs with more than 300 households were divided into 100 to 150 household segments. Hence, one cluster can be a PSU or a segment of a PSU. The original sample size for NFHS-4 was 112,122 men aged 15 years or above, and 699,686 women aged 15–49 years. After excluding 8,597 men sample above 49 years, the final analytic sample was 103,525 men and 699,686 women aged 15–49 years for NFHS-4 (2015–16). For NFHS-5, the original sample was 990,474 men (15 years or above). After excluding the sample men above 49 years, the final sample was 700,561 men and 724,115 women aged 15–49 years (Supplementary File 1—Figure S1).

### Primary outcomes and geographies

The primary outcome variables were binary (Yes = 1/No = 0) coded responses for alcohol and tobacco consumption in any form among men and women separately in both survey rounds. It is worth mentioning here that we followed the NFHS report for deriving the variable for alcohol and tobacco consumption. The survey questions regarding alcohol and tobacco consumption were general (global/overall sense) without reference to the frequency, magnitude (quantity of alcohol), and time period of consumption. For alcohol, the survey question was *“Do you drink alcohol?”*. However, the binary variable for tobacco consumption was created based on four questions related to the consumption forms, i.e., (a) chew tobacco; (b) smokes/uses gutkha/paan masala with tobacco; (c) smokes/uses paan with tobacco; (d) does not use cigarettes and tobacco. The analyses included estimates for 720 districts nested within 36 states and Union Territories (UTs).

### Statistical analyses

To compute the variation across geographical units and estimate the district-level precision-weighted estimates for the prevalence of alcohol and tobacco consumption, we employed a four-level logistic regression with individual *i* (level-1); cluster *j* (level-2); district *k* (level-3); state *l* (level-4): ${Y}_{ijkl}= {\beta }_{0}+({u}_{0jkl}+ {v}_{0kl}+ {f}_{0l})$. In the model mentioned, ${u}_{0jkl}$, ${v}_{0kl}$, ${f}_{0l}$ are model residuals specific to cluster, district, and state, respectively. These sets of residuals are assumed to have a normal distribution around the mean of 0 and the variance of ${u}_{0jkl}$ ~ (0, ${\sigma }_{\text{u}0}^{2}$); ${v}_{0kl}$ ~ (0, ${\sigma }_{\text{v}0}^{2}$); ${f}_{0l}$ ~ (0, ${\sigma }_{\text{f}0}^{2}$). Here, the term ${\sigma }_{\text{u}0}^{2}$ denotes within-district, inter-cluster variation, ${\sigma }_{\text{v}0}^{2}$ denotes within-state, inter-district variation, and ${\sigma }_{\text{f}0}^{2}$ stands for interstate variation. Variance across individual men and women is not computed directly for binary outcomes and is instead assumed to follow a logistic distribution with a fixed variance of ${\pi }^{2}$/3 or 3.29 [21]. We then computed the variance partitioning coefficient to assess the significance of each geographical unit (z) in total variability as ($\frac{{\sigma }_{z}^{2}}{{\sigma }_{u0}^{2}+ {\sigma }_{v0}^{2}+ {\sigma }_{f0}^{2}}$) * 100.

Based on the multilevel logistic model estimates, we then generated precision-weighted cluster-level predicted probabilities of alcohol and tobacco consumption for both men and women. For more robust estimates, these probabilities were predicted by pooling information (and borrowing strength) from other clusters that share the same district membership. The probability of each Y for each cluster was calculated as exp((${\beta }_{0}+{u}_{0jkl}+ {v}_{0kl}+ {f}_{0l})+(1/\text{exp}({\beta }_{0}+{u}_{0jkl}+ {v}_{0kl}+ {f}_{0l}))$. Finally, we estimated prevalence (%) for 720 districts by taking the mean of cluster-level predicted probabilities for the updated district boundaries. Multilevel modelling was performed using the STATA 15 and MLwiN 3.09 software program (using *runmlwin*) and the Markov Chain Monte Carlo (MCMC) method using the Gibbs sampler, keeping the default prior distribution of Iterated Generalized Least Squares (IGLS) as the starting value [22–24]. The logs of model output containing details about model specifications are presented as supplementary (Exhibit S1).

To infer the overtime district-level change in alcohol and tobacco consumption, we based the cut-off points – to classify the magnitude of change – on the distribution of the predicted estimates. This ensured a meaningful and interpretable threshold for classifying change. Given that NFHS-4 and NFHS-5 are large-scale, nationally representative surveys with substantial sample sizes, estimated differences were statistically significant. Therefore, we focused on substantive changes in prevalence over time. We considered a shift of less than 2.5 percentage points over the five years as “no change,” as such variations are expected and perhaps not meaningful. Any change exceeding 2.5 percentage points was classified as improvement or worsening and further categorized into three levels based on the distribution of the data. Specifically, we considered the following categories for positive as well as negative change: (a) No Change: Between 0 to 2.49 (increase) or 0 to −2.49 (decrease); (b) Small Change: Between 2.50 to 4.99 (increase) or −2.50 to −4.99 (decrease); (c) Moderate Change: Between 5.00 to 9.99 (increase) or −5.00 to −9.99 (decrease); (d) Substantial Change: 10.00 and above (increase) or −10.00 and below (decrease).

## Results

### Geographic variation in alcohol and tobacco consumption

Between 2016 and 2021, alcohol and tobacco consumption among men in India declined significantly, with alcohol usage dropping from 29.2% to 17.5% and tobacco from 44.5% to 32.6% (Table 1). Lakshadweep (0.5%), Gujarat (5%), and Jammu and Kashmir (7.5%) recorded the lowest alcohol prevalence, while Arunachal Pradesh (49.2%) and Telangana (39.2%) reported the highest alcohol prevalence in 2021. Prevalence of tobacco consumption was the highest in Mizoram (72.5%) and the lowest in Chandigarh (10.4%). States like Tamil Nadu and Tripura reported sharp declines in the prevalence of alcohol consumption, while Madhya Pradesh reported the highest reduction in tobacco consumption. Interestingly, a high correlation between the prevalence across the two survey rounds was observed (Figure S2). These patterns were consistent among women, i.e., the consumption of both alcohol and tobacco has declined among women as well.

**Table 1:** Prevalence of alcohol and tobacco consumption among adults (15–49 years) across States, India, NFHS, 2016–2021

States	Alcohol						Tobacco					
	Men			Women			Men			Women		
	2016 (%)	2021 (%)	Change (% points)	2016 (%)	2021 (%)	Change (% points)	2016 (%)	2021 (%)	Change (% points)	2016 (%)	2021 (%)	Change (% points)
Jammu & Kashmir	10.3	7.5	−2.8^c^	0.1	0.2	0.1^b^	38.2	32.2	−6.0^c^	2.8	1.2	−1.6^c^
Himachal Pradesh	39.8	25.9	−13.9^c^	0.3	0.4	0.1	40.6	24.8	−15.8^c^	0.5	0.2	−0.3^b^
Punjab	34.1	20	−14.1^c^	0.1	0.1	0	19.2	11.3	−7.9^c^	0.1	0.1	0
Chandigarh	39.3	16.9	−22.4^c^	0.5	0.1	−0.4	22.5	10.4	−12.1^c^	0.4	0	−0.4^a^
Uttarakhand	35.2	20.5	−14.7^c^	0.3	0.1	−0.2^a^	43.7	25.8	−17.9^c^	2.9	0.8	−2.1^c^
Haryana	24.5	14	−10.5^c^	0.1	0.1	0	35.8	22.5	−13.3^c^	1.6	0.4	−1.2^c^
NCT of Delhi	24.5	19.8	−4.7^b^	0.6	1.5	0.9^c^	30.3	23	−7.3^c^	1.6	1.6	0
Rajasthan	15.9	9.8	−6.1^c^	0.1	0.1	0	46.9	35	−11.9^c^	6.3	3.8	−2.5^c^
Uttar Pradesh	22.1	13.8	−8.3^c^	0.1	0.1	0^c^	53	37.3	−15.7^c^	7.6	3.3	−4.3^c^
Bihar	28.9	15.9	−13.0^c^	0.2	0.1	−0.1^c^	50.1	40	−10.1^c^	2.8	0.7	−2.1^c^
Sikkim	51.2	37.9	−13.3^c^	23	14.8	−8.2^c^	40.3	40.3	0	7.3	8.7	1.4
Arunachal Pradesh	59.3	49.2	−10.1^c^	26.3	17.8	−8.5^c^	60.2	47	−13.2^c^	18	10.7	−7.3^c^
Nagaland	38.8	28.7	−10.1^c^	3.3	1.4	−1.9^c^	69.2	54.1	−15.1^c^	27.4	14.1	−13.3^c^
Manipur	52.6	38.5	−14.1^c^	6.1	1.6	−4.5^c^	70.6	57.2	−13.4^c^	48.8	41.6	−7.2^c^
Mizoram	49.5	28.2	−21.3^c^	4.9	1	−3.9^c^	80.5	72.5	−8.0^c^	59.5	48.1	−11.4^c^
Tripura	57.2	34.1	−23.1^c^	4.8	4.4	−0.4	67.6	50.7	−16.9^c^	42.2	39.3	−2.9^b^
Meghalaya	44.6	31.3	−13.3^c^	2.1	1.1	−1^c^	72.2	54.9	−17.3^c^	32.5	25.3	−7.2^c^
Assam	35.4	24.7	−10.7^c^	7	5.6	−1.4^c^	63.8	48.1	−15.7^c^	19.8	13.3	−6.5^c^
West Bengal	28.7	18.9	−9.8^c^	0.8	0.7	−0.1	58.8	43.9	−14.9^c^	8.7	5.7	−3.0^c^
Jharkhand	39.3	32.3	−7.0^c^	4.1	2.6	−1.5^c^	48.7	40.3	−8.4^c^	5.8	2.4	−3.4^c^
Odisha	39.3	29.6	−9.7^c^	2.3	2.7	0.4^b^	55.9	45.1	−10.8^c^	17.3	12.6	−4.7^c^
Chhattisgarh	52.7	31.9	−20.8^c^	5	2.8	−2.2^c^	55.2	37.8	−17.4^c^	21.7	7.8	−13.9^c^
Madhya Pradesh	29.6	16	−13.6^c^	1.6	0.4	−1.2^c^	59.5	40.8	−18.7^c^	10.4	6.1	−4.3^c^
Gujarat	11	5	−6^c^	0.3	0.1	−0.2^b^	51.5	38.1	−13.4^c^	7.4	5.6	−1.8^c^
DN & DD	34.7	27.1	−7.6^c^	0.5	0.5	0	36.5	37.2	0.7	1.5	1.2	−0.3
Maharashtra	20.8	12.6	−8.2^c^	0.2	0.1	−0.1	36.5	28.9	−7.6^c^	5.8	4.4	−1.4^c^
Karnataka	29.3	14.3	−15.0^c^	1	0.3	−0.7^b^	34.3	23	−11.3^c^	4.2	2.7	−1.5^c^
Goa	44.7	33.5	−11.2^c^	4.2	4.8	0.6^c^	20.8	16.1	−4.7^b^	1.9	1	−0.9^b^
Lakshadweep	5.4	0.5	−4.9^c^	0	0.1	0.1	24.7	23.9	−0.8	16.4	4.4	−12.0^c^
Kerala	37	16.5	−20.5^c^	1.6	0.3	−1.3^c^	25.7	12	−13.7^c^	0.8	0.4	−0.4^c^
Tamil Nadu	46.9	23.5	−23.4^c^	0.4	0.1	−0.3^c^	31.9	15.3	−16.6^c^	2.2	1.1	−1.1^c^
Puducherry	41	24.7	−16.3^c^	0.6	0.2	−0.4^a^	14.4	11.9	−2.5	1.1	0.2	−0.9^c^
Andaman & Nicobar Islands	51.7	38.4	−13.3^c^	2.5	2.2	−0.3	61.6	53.6	−8^b^	25.1	17.5	−7.6^c^
Telangana	53.8	39.2	−14.6^c^	8.7	5	−3.7^c^	28.2	16.4	−11.8	2.8	1.8	−1^c^
Andhra Pradesh	34.8	20.4	−14.4^c^	0.4	0.2	−0.2^c^	26.7	16	−10.7^c^	2.3	1.2	−1.1^c^
Ladakh	22.7	20.5	−2.2^c^	2.8	3.6	0.8	23.6	31.5	7.9	0.5	2.6	2.1
All India	29.2	17.5	−11.7^c^	1.2	0.8	−0.5^c^	44.5	32.6	−11.9^c^	6.8	4.1	−2.7^c^

Estimates are ^a^significant at 90%, ^b^ at 95%, ^c^ at 99 s%, DN & DD refers to Dadra Nagar Haveli and Daman & Diu

### Variance decomposition across geographies

**Fig. 1:**
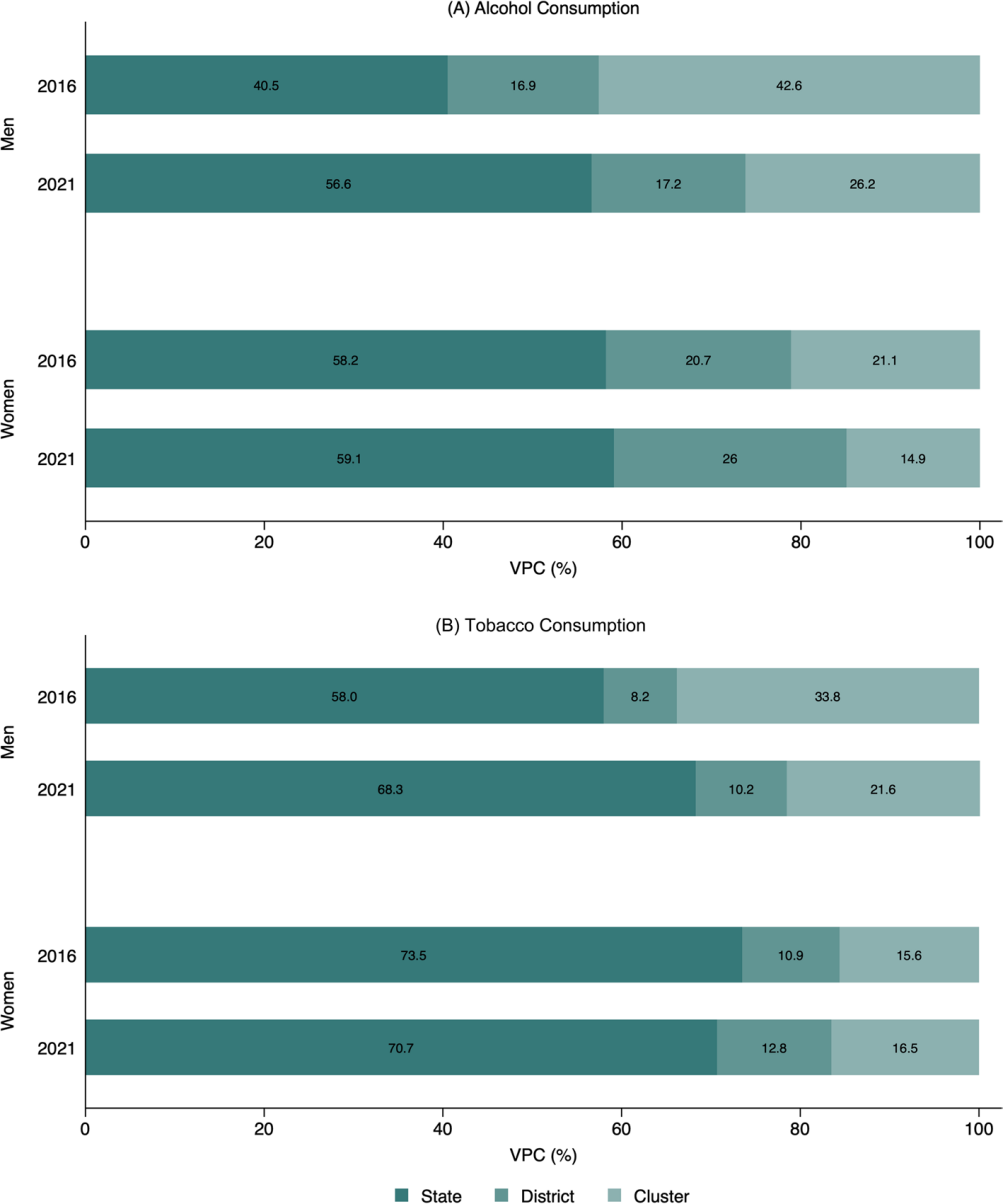
Geographic variance partitioning coefficient (VPC) (%) by clusters, districts, and states for alcohol and tobacco consumption among men and women, India, NFHS, 2016–2021

The variations in alcohol prevalence among men in 2016 attributable to different geographic levels were 40.5% to states, 16.9% to districts, and 42.6% to clusters (Fig. 1). The variations in tobacco prevalence attributable to different geographic levels were 58% to states, 8.2% to districts, and 33.8% to clusters. Interestingly, the variation attributable to states for both alcohol (40.5% in 2016 and 56.6% in 2021) and tobacco (58% in 2016 and 68.3% in 2021) was higher in 2021 as compared to 2016. The variations in prevalence of alcohol and tobacco have reduced at the cluster level. Similar geographic variations were observed with respect to the prevalence of consumption of tobacco and alcohol among women as well.

### District-level estimates of alcohol and tobacco consumption

**Fig. 2:**
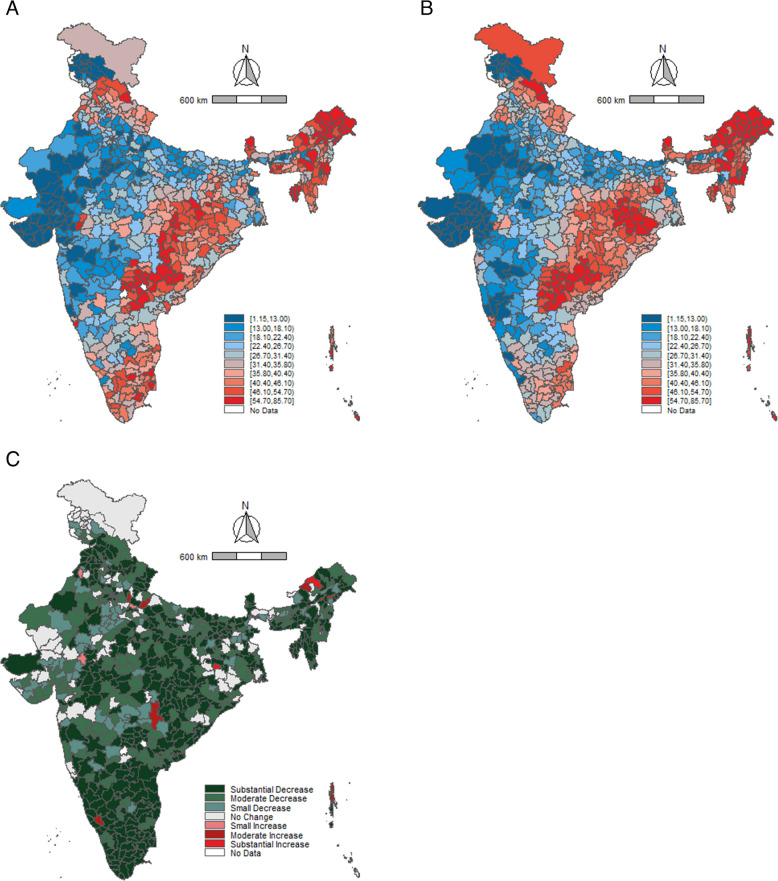
Prevalence (%) of alcohol consumption among men (15–49 years) across districts, India, NFHS, 2016–2021. Note: Prevalence cut points based on decile positions of 2016 consumption (Panels (**A**) and (**B**)). Cut-points for change (2021–2016) (Panel (**C**)): Substantial decrease (> 10.00%, dark green), Moderate decrease (5.00–9.99%, green), Small decrease (2.50–4.99%, light green), No change (−2.49 to 2.49%, gray), Small increase (2.50–4.99%, light red), Moderate increase (5.00–9.99%, red), Substantial increase (> 10.00%, dark red)

**Fig. 3:**
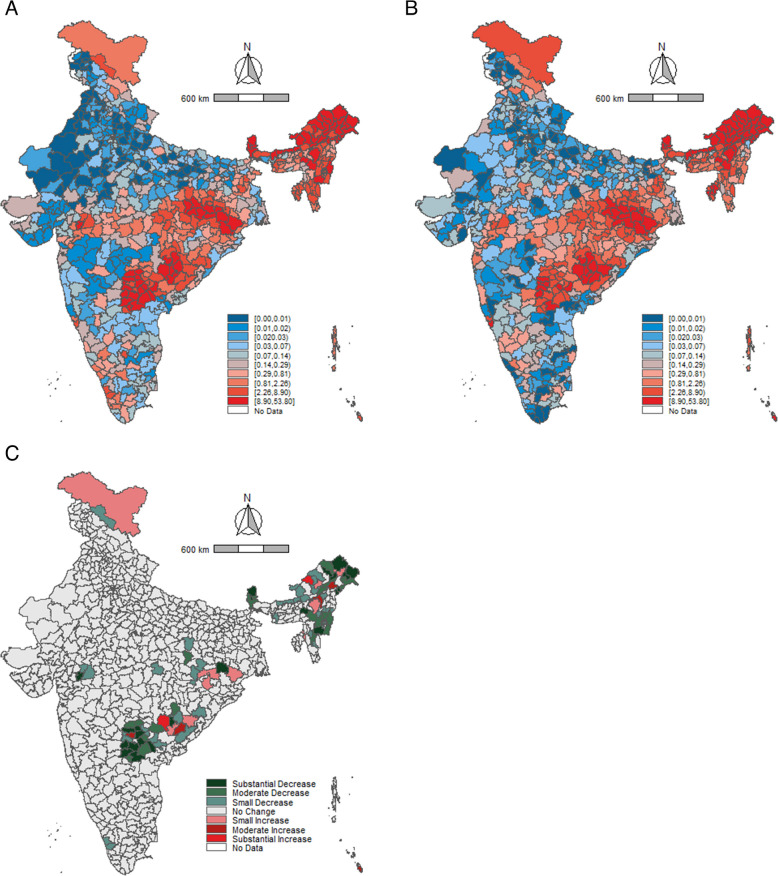
Prevalence (%) of alcohol consumption among women (15–49 years) across districts, India, NFHS, 2016–2021. Note: Prevalence cut points based on decile positions of 2016 consumption (Panels (**A**) and (**B**)). Cut-points for change (2021–2016) (Panel (**C**)): Substantial decrease (> 10.00%, dark green), Moderate decrease (5.00–9.99%, green), Small decrease (2.50–4.99%, light green), No change (−2.49 to 2.49%, gray), Small increase (2.50–4.99%, light red), Moderate increase (5.00–9.99%, red), Substantial increase (> 10.00%, dark red)

**Fig. 4:**
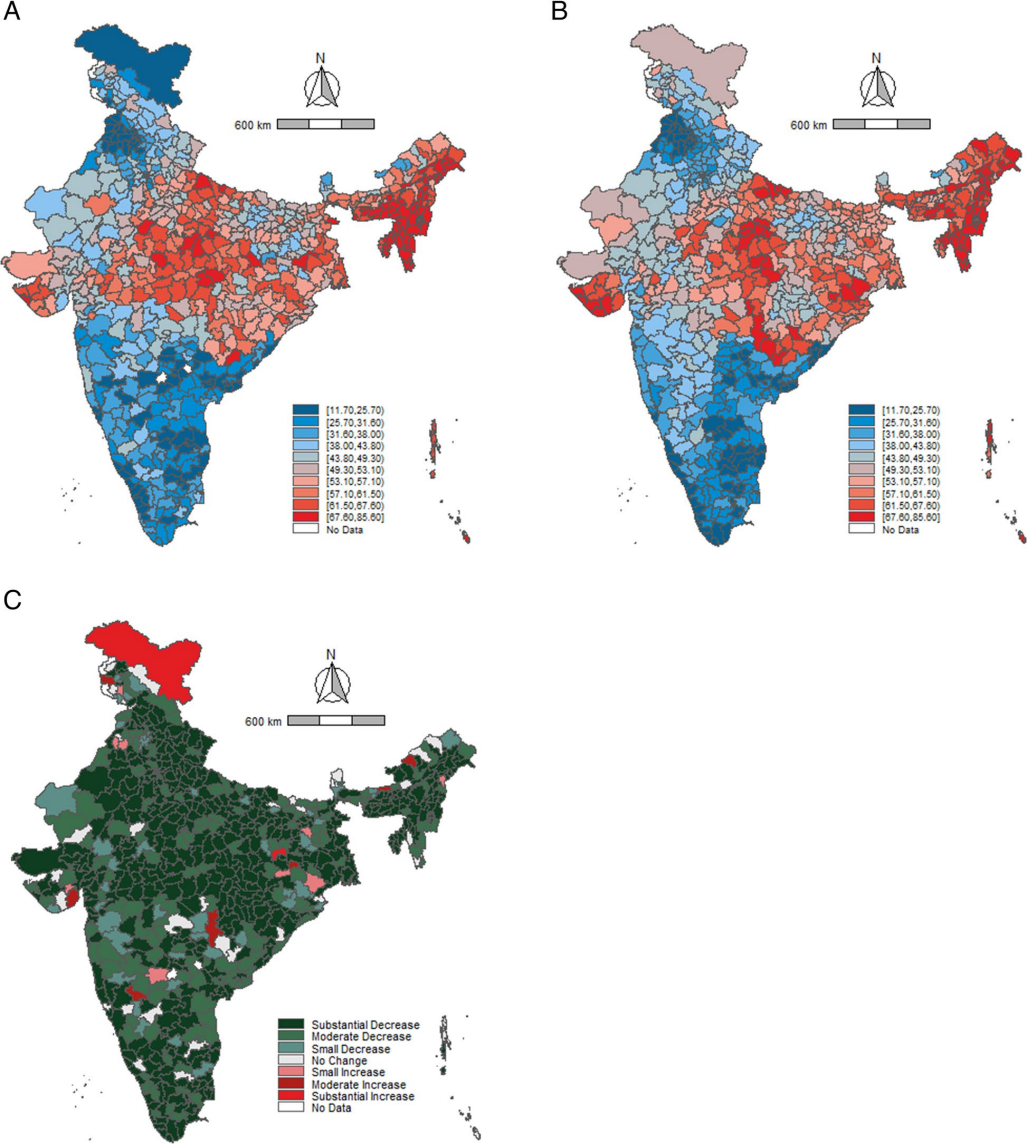
Prevalence (%) tobacco consumption among men (15–49 years) across districts, India, NFHS, 2016–2021. Note: Prevalence cut points based on decile positions of 2016 consumption (Panels (**A**) and (**B**)). Cut-points for change (2021–2016) (Panel (**C**)): Substantial decrease (> 10.00%, dark green), Moderate decrease (5.00–9.99%, green), Small decrease (2.50–4.99%, light green), No change (−2.49 to 2.49%, gray), Small increase (2.50–4.99%, light red), Moderate increase (5.00–9.99%, red), Substantial increase (> 10.00%, dark red)

**Fig. 5:**
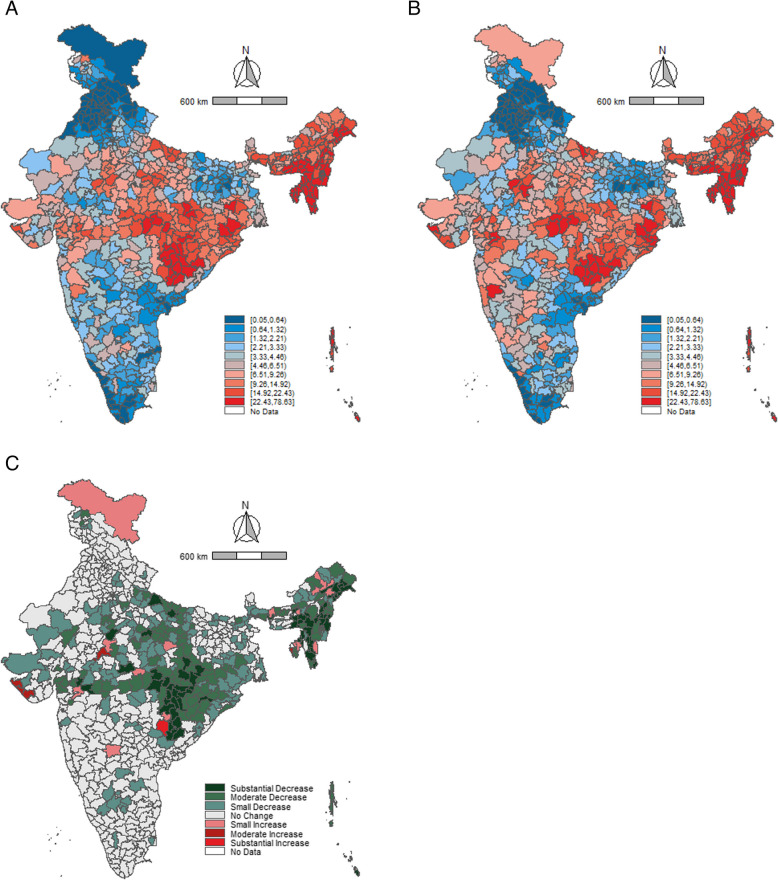
Prevalence (%) of tobacco consumption among women (15–49 years) across districts, India, NFHS, 2016–2021. Note: Prevalence cut points based on decile positions of 2016 consumption (Panels (**A**) and (**B**)). Cut-points for change (2021–2016) (Panel (**C**)): Substantial decrease (> 10.00%, dark green), Moderate decrease (5.00–9.99%, green), Small decrease (2.50–4.99%, light green), No change (−2.49 to 2.49%, gray), Small increase (2.50–4.99%, light red), Moderate increase (5.00–9.99%, red), Substantial increase (> 10.00%, dark red)

A higher number of districts in Rajasthan and Uttar Pradesh reported low consumption of alcohol in both 2016 as well as 2021 (Fig. 2A and B). The maps elicit a notable concentration of high-consumption districts in the north-east and south-east parts of the country. Between 2016 and 2021, almost all districts showed a reduction in alcohol consumption among men (Fig. 2C). Prominent reductions were observed in the southern region districts from the states of Kerala, Tamil Nadu, Karnataka, and Andhra Pradesh. The (Fig. 3A and B) show a similar pattern of alcohol-consumption prevalence among women. Further, a high number of districts in Telangana and Uttarakhand reported low consumption of tobacco prevalence in 2016 and 2021 (Fig. 4A and B, 5A and B). A large number of districts across north-eastern states such as Manipur, Meghalaya, Nagaland, and Mizoram reported a high burden of tobacco consumption. As of 2021, the prevalence of tobacco consumption was higher across all the districts in Arunachal Pradesh, Goa, Manipur, and Sikkim. It can be observed that districts from northern and central India have reported a substantial decrease in the tobacco consumption among men between 2016 and 2021 (Fig. 4C). The geographical clustering for tobacco consumption among women also showed a similar pattern (Fig. 5C).

### Distribution of the number of districts by absolute change (% points)

**Fig. 6:**
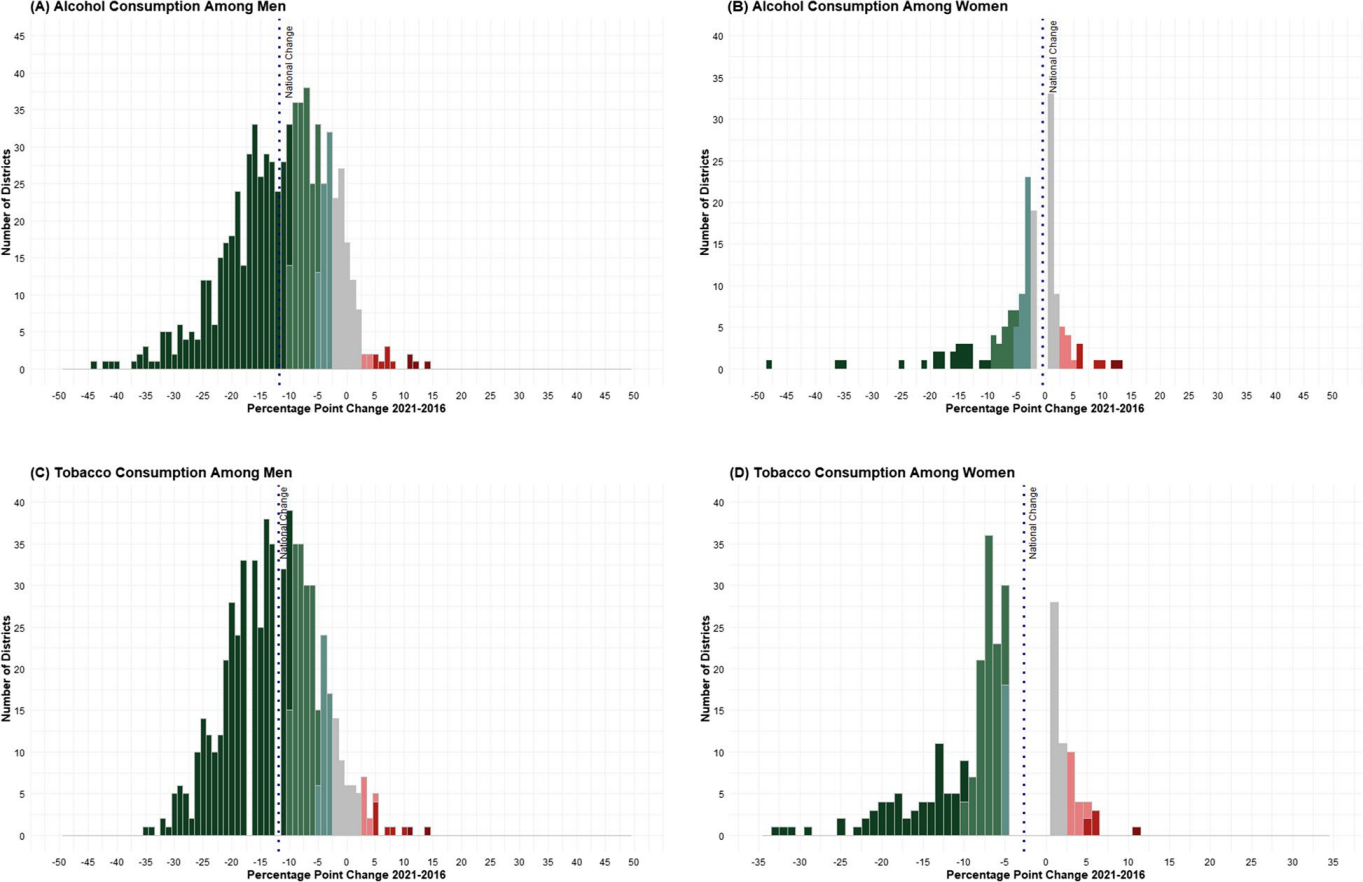
Distribution of change (% points) in the prevalence of alcohol and tobacco consumption among adults (15–49 years) across districts, India, NFHS, 2016–2021. Note: Cut-points for change (2021–2016): Substantial decrease (> 10.00%, dark green), Moderate decrease (5.00–9.99%, green), Small decrease (2.50–4.99%, light green), No change (−2.49 to 2.49%, gray), Small increase (2.50–4.99%, light red), Moderate increase (5.00–9.99%, red), Substantial increase (> 10.00%, dark red)

Figure 6 presents the distribution of the number of districts by absolute change (% points). The districts were distributed based on the following categories: Substantial Decrease (> 10.00% points), Moderate Decrease (5.00–9.99% points), Small Decrease (2.50–4.99% points), No Change (−2.49 to 2.49% points), Small Increase (2.50–4.99% points), Moderate Increase (5.00–9.99% points), Substantial Increase (> 10.00% points). More than 80% of the districts reported a moderate to substantial reduction in the prevalence of alcohol consumption among men between the two rounds (Fig. 6A and B). These patterns were similar for the consumption of alcohol and tobacco among women as well (Fig. 6C and D).

## Discussion

This study aimed to present the change in tobacco and alcohol prevalence based on two rounds of the nationally representative sample survey. The four salient findings from the study are as follows. First, alcohol and tobacco consumption among both men and women in India has declined between 2016 and 2021. Second, variance partitioning results reveal that the states and clusters accounted for the highest variation, followed by the lowest variation at the district level. Third, a large number of districts in north-eastern states such as Manipur, Meghalaya, Nagaland, and Mizoram reported a high prevalence of tobacco consumption. Fourth, the reduction in the prevalence of alcohol and tobacco was not uniform across the districts. We elaborate on each of these findings in the subsequent paragraphs.

First, alcohol and tobacco consumption among both men and women in India has declined between the two rounds of NFHS. States like Tamil Nadu and Tripura reported sharp declines in the prevalence of alcohol consumption, while Madhya Pradesh reported the highest reduction in tobacco consumption. The consumption of alcohol was the lowest in Gujarat. The consumption of both tobacco and alcohol was higher in the north-eastern region. Our findings are in line with similar studies based on previous rounds of NFHS as well as the Global Adult Tobacco Survey (GATS), asserting over time decline in the consumption of tobacco as well as alcohol in the country [25–27]. Previous studies have found that tobacco consumption in particular was high across the northeast region, which is corroborated by our findings as well [27–29]. In addition, a high correlation between the prevalence across the two survey rounds was observed. This suggests that both surveys are likely capturing similar behaviours, validating the reliability of the findings that consumption remains high across states with high prevalence.

The reduction in tobacco consumption is likely due to increased taxes over time and the imposition of additional cess, which has increased the price of the product substantially [30]. However, there remains considerable potential to further increase taxes on tobacco products [31]. For example, the current tax structure imposes lower taxes on bidis and similar products compared to cigarettes, making bidis more affordable [31]. Additionally, significant price variations across states contribute to the uneven affordability of tobacco products [31]. Addressing these gaps could help reduce tobacco consumption further. In addition, several studies have found that socio-economic factors, such as education, wealth, and age, as well as cultural norms, also play a significant role in determining the consumption of alcohol and tobacco, for which behaviour change interventions are warranted [29, 32, 33].

Second, the variance attributable to states is significantly higher than that at the district or cluster levels. This underscores the potential bias in relying solely on estimates derived from a single level of analysis. Previous studies on geospatial patterns of alcohol and tobacco use similarly found a high concentration in north-eastern states [28]. Our findings further indicate a much higher clustering of tobacco use at the cluster level than previously reported, highlighting the importance of local contexts and contextual determinants in India [34, 35]. Tobacco use remains disproportionately high among individuals from lower socioeconomic backgrounds, among scheduled caste groups and among uneducated adults. In lower-income neighbourhoods with limited health awareness with a weaker tobacco-control policy environment, the likelihood of initiation and continued use is greater [29, 36, 37]. These variations are likely influenced by differences in the availability and enforcement of tobacco control policies, the social environment, as well as shared cultural and social norms regarding tobacco use [32, 33]. This finding assumes further attention, given that the prevalence of cancers is strongly correlated with high alcohol and tobacco consumption [38, 39]. A recent observational study has identified smoking and tobacco use as one of the main risk factors for NCDs among women [14]. This is distressing since the NCDs burden among women have serious implications for child health, emphasising the urgency of addressing these issues.

Third, a large number of districts in north-eastern states such as Manipur, Meghalaya, Nagaland, and Mizoram reported a high prevalence of tobacco consumption. As of 2021, alcohol consumption was notably higher in districts of Arunachal Pradesh, Goa, Manipur, and Sikkim. The high prevalence of alcohol use in these regions is closely linked to social customs and ease of availability. Alcohol is a widely accepted norm at social events and, in many north-eastern states, traditional brewing is embedded in tribal customs, making alcohol consumption a culturally accepted practice [40–42]. This distribution may reflect differences in the implementation and effectiveness of tobacco control policies. For instance, in these states, the consumption of smokeless tobacco and bidis remains high, suggesting that existing policies have been less effective in targeting these products [31, 43]. In contrast, the low alcohol consumption levels in Gujarat highlight the success of the state’s long-standing prohibition policy, first enacted under the Gujarat Prohibition Act, 1949, prohibiting the manufacture, sale, and consumption of alcohol. This act has been further amended multiple times in the past decades to include special courts and harsher penalties, creating a more stringent and comprehensive regulatory framework to restrict alcohol use [44]. These findings emphasise the need to revisit and redesign existing policies to address current gaps and improve their effectiveness.

Fourth, the reduction in the prevalence of alcohol and tobacco was not uniform across the districts. For instance, a higher number of the districts in Mizoram, Tamil Nadu, and Kerala have reported a high reduction in the prevalence of alcohol while a large number of districts in Uttarakhand and Meghalaya reported a high reduction in the consumption of tobacco. These findings could be again attributable to how the states differ in how strictly they enforce regulations on alcohol and tobacco, such as taxation, bans, and restrictions on advertising [32, 45–47]. For example, the Mizoram Liquor Prohibition Act, 2019, which instituted a state-wide ban on alcohol, and the implementation of the National Tobacco Control Programme (NTCP) in Meghalaya, which strengthened enforcement of smoke-free laws and advertising restrictions, highlight the potential influence of state-level policy interventions on district-level substance use outcomes [48, 49]. Additionally, the shift to a higher socioeconomic status may also explain the lower alcohol consumption observed, as highlighted in several small sample-based studies [50].

## Limitations

The following are the limitations of this study. Firstly, the data used in the study are cross-sectional. Therefore, it is not possible to do a comparative analysis over a longer period of time for the same households. Second, the sample size was very small for some of the States and Union Territories, so it was difficult to obtain reliable estimates. For this reason, we have presented the broad trends and patterns and have not controlled for the socioeconomic characteristics. Third, NFHS has used the sampling frame of the 2011 Census for sampling. The data is available for 640 districts for 2016. However, in 2021, there were more than 700 districts. However, we believe that the same trends in multi-level variation will remain. Additionally, because of the nature of the data, the lowest geographic level we could observe was the PSU. Furthermore, tobacco and alcohol consumption are usually underreported in national surveys due to desirability bias and the presence of other people. Therefore, the results of this study might conceal the fact that the prevalence could be much higher.

## Conclusions

Overall, the decline in tobacco and alcohol prevalence indicates progress; however, there remains a need for continuous and targeted interventions such as taxation hikes and behavioural interventions. Introducing behaviour cessation programmes amongst adolescents and younger adults and in primary healthcare systems has demonstrated sustained effects on abstinence and use reduction [51, 52]. The results of the present study indicate that interventions focusing on changing tobacco and alcohol consumption must consider the geographical variation. By implementing evidence-based policies and interventions suited to the needs of the local areas, public health authorities can continue to make significant strides in improving the health and well-being of the population and reducing the burden of tobacco and alcohol-related diseases.

The authors declare that they have no competing interests.

## Supplementary Information

Supplementary Material 1.

## Data Availability

The study is based on publicly available data and can be accessed from https://dhsprogram.com/data/available-datasets.cfm.

## Abbreviations

UT: Union Territory
NFHS: National Family Health Survey
NCD: Non-communicable Disease
DHS: Demographic and Health Surveys
CEB: Census Enumeration Block
PSU: Primary Sampling Unit
SC: Scheduled Caste
ST: Scheduled Tribe
MCMC: Markov Chain Monte Carlo
IGLS: Iterated Generalized Least Squares
GATS: Global Adult Tobacco Survey
NTCP: National Tobacco Control Programme
