# Supplementary material for: Changes in prevalence of alcohol and tobacco consumption across districts of India, 2016 and 2021
Source: BMC Public Health. 2025 May 27;25:1962. doi: 10.1186/s12889-025-23029-z (PMC12108003; doi:10.1186/s12889-025-23029-z)
Supplement: Supplementary file 1 — Supplementary Material 1. [file 12889_2025_23029_MOESM1_ESM.docx]

Supplementary to:

Changes in Prevalence of Alcohol and Tobacco Consumption Across Districts of India, 2016 and 2021

Figure S1: Flow diagram showing exclusions and final sample size for primary analysis of the study population, NFHS (2016 and 2021)

NFHS Original Sample Size

Men

NFHS 4: 259, 627 (MR)

NFHS 5: 2,843,917 (PR)

Women

NFHS 4: 699,686 (IR)

NFHS 5: 724,115 (IR)

**Sample Exclusion (Criteria-wise)**

| Category (Men) | NFHS 4 | NFHS 5 |
| --- | --- | --- |
| Beyond 15-49 bracket | 8597 | 1853443 |

**Final Analytical Sample**

Men (15-49 years)

**NFHS 4: 103,525**

**NFHS 5: 990,474**

Women (15-49 years)

**NFHS 4: 699,686**

**NFHS 5: 724,115**

Figure S2: Association Between Alcohol and Tobacco Consumption in 2016 and 2021 among Men and Women aged 15-49 years NFHS 2016-21, India


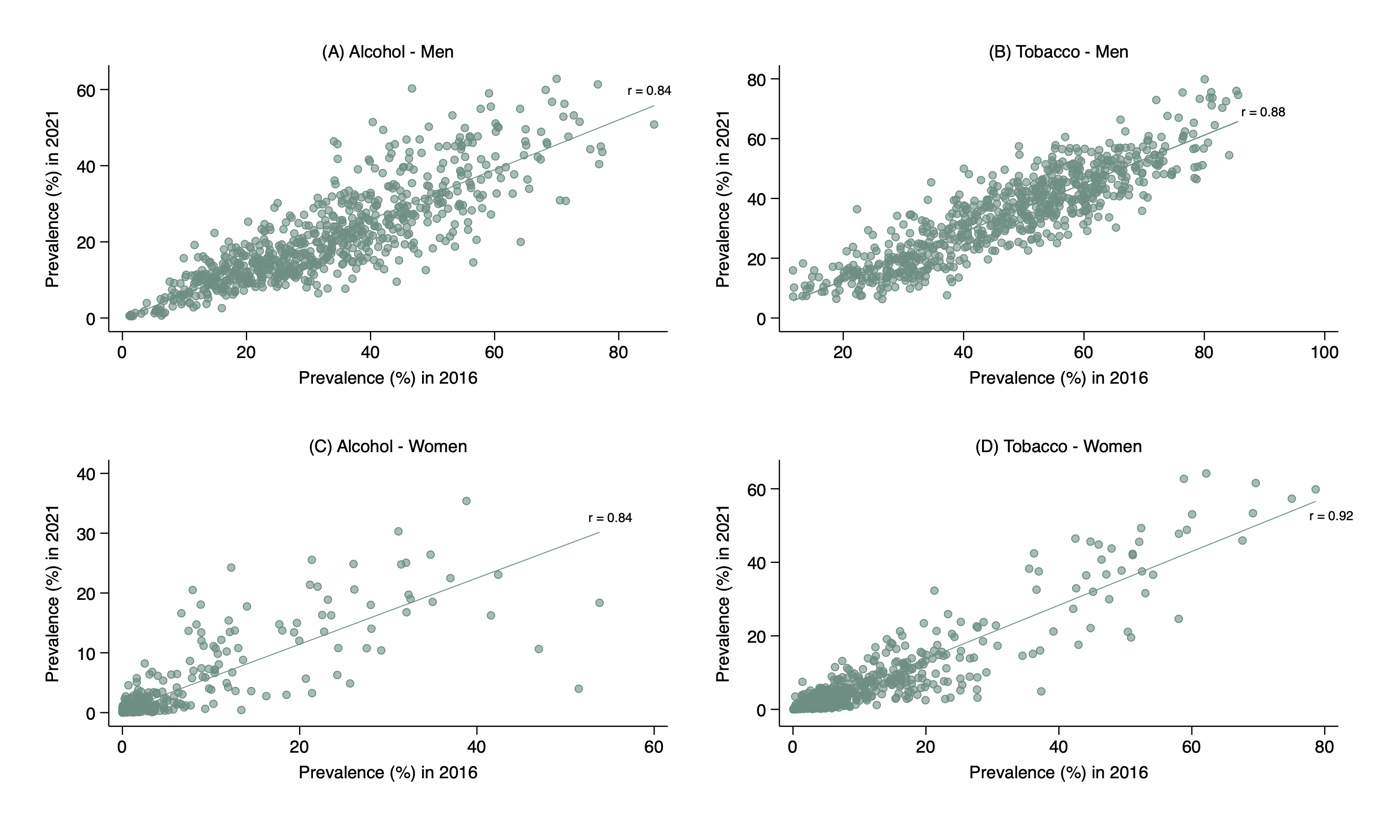


Note: r = Pearson’s correlation coefficient

Exhibit S1 - Estimates from the Four-level random effects Model (MCMC) for all Outcomes, India, NFHS 2016-2021

| Indicator | Beta (Cons) | ESS | Var (cons) - State | ESS | Var (cons) -District | ESS | Var (cons) -Cluster | ESS |
| --- | --- | --- | --- | --- | --- | --- | --- | --- |
| Alcohol Men 2016 | ‐.724 | 3221 | -0.160 | 2041 | 0.270 | 419 | 0.680 | 196 |
| *Standard Deviation* | *-0.138* |  | *0.184* |  | *0.022* |  | *0.022* |  |
| Alcohol Men 2021 | ‐1.457 | 3538 | -0.876 | 1059 | 0.266 | 768 | 0.406 | 326 |
| *Standard Deviation* | *-0.160* |  | *0.249* |  | *0.017* |  | *0.007* |  |
| Alcohol Women 2016 | ‐6.017 | 2814 | 5.919 | 8 | 2.108 | 5 | 2.149 | 34 |
| *Standard Deviation* | *-0.42* |  | *2.259* |  | *0.522* |  | *0.067* |  |
| Alcohol Women 2021 | -6.482 | 3375 | 8.470 | 4 | 3.725 | 3 | 2.127 | 25 |
| *Standard Deviation* | *-0.509* |  | *5.021* |  | *2.479* |  | *0.079* |  |
| Tobacco Men 2016 | ‐.1981063 | 3871 | 0.710 | 3125 | 0.100 | 329 | 0.414 | 193 |
| *Standard Deviation* | *-0.144* |  | *0.188* |  | *0.009* |  | *0.015* |  |
| Tobacco Men 2021 | ‐.7547479 | 4197 | 0.747 | 3046 | 0.111 | 1500 | 0.236 | 270 |
| *Standard Deviation* | *-0.145* |  | *0.196* |  | *0.007* |  | *0.004* |  |
| Tobacco Women 2016 | -3.222 | 4033 | 3.375 | 3269 | 0.501 | 1130 | 0.719 | 190 |
| *Standard Deviation* | *-0.310* |  | *0.901* |  | *0.033* |  | *0.013* |  |
| Tobacco Women 2021 | ‐4.083131 | 4054 | 4.209 | 2544 | 0.761 | 1212 | 0.985 | 120 |
| Standard Deviation | *-0.344* |  | *1.145* |  | *0.049* |  | *0.021* |  |

Note: ESS- Effective Sample Size ; Cons – Constant ; Vars - Variance

Table S1: Percentage Prevalence of Alcohol and Tobacco Consumption Among Men (15-49 years) Across Districts of India, NFHS 2016-21

| District Name | Alcohol | |  | Tobacco | |  |
| --- | --- | --- | --- | --- | --- | --- |
|  | 2016 | 2021 | AAC | 2016 | 2021 | AAC |
| Alluri Sitharama Raju | 42.8 | 27.2 | -3.1 | 29.1 | 20.2 | -1.8 |
| Anakapalli | 46.0 | 27.4 | -3.7 | 27.9 | 15.2 | -2.5 |
| Ananthapuramu | 27.2 | 12.5 | -2.9 | 27.2 | 13.4 | -2.8 |
| Annamayya | 32.6 | 15.2 | -3.5 | 22.3 | 14.5 | -1.6 |
| Bapatla | 33.9 | 19.8 | -2.8 | 33.2 | 20.4 | -2.6 |
| Chittoor | 32.2 | 14.7 | -3.5 | 16.0 | 13.7 | -0.5 |
| Dr. B.R. Ambedkar Konaseema | 37.1 | 21.2 | -3.2 | 22.1 | 14.3 | -1.5 |
| East Godavari | 34.4 | 21.8 | -2.5 | 27.8 | 15.5 | -2.5 |
| Eluru | 27.1 | 19.0 | -1.6 | 21.7 | 14.1 | -1.5 |
| Guntur | 28.8 | 19.3 | -1.9 | 31.4 | 17.4 | -2.8 |
| Kakinada | 35.1 | 25.6 | -1.9 | 24.5 | 16.5 | -1.6 |
| Krishna | 53.6 | 18.8 | -7.0 | 29.4 | 15.2 | -2.8 |
| Kurnool | 33.4 | 17.2 | -3.2 | 31.0 | 15.3 | -3.1 |
| Nandyal | 30.7 | 15.3 | -3.1 | 33.0 | 15.6 | -3.5 |
| Ntr | 28.1 | 19.7 | -1.7 | 20.8 | 15.8 | -1.0 |
| Palnadu | 29.1 | 20.1 | -1.8 | 29.2 | 18.9 | -2.1 |
| Parvathipuram Manyam | 46.9 | 27.3 | -3.9 | 32.7 | 13.4 | -3.9 |
| Prakasam | 27.4 | 17.3 | -2.0 | 30.2 | 20.7 | -1.9 |
| Sri Potti Sriramulu Nellore | 36.0 | 22.3 | -2.7 | 28.9 | 19.7 | -1.8 |
| Sri Sathya Sai | 30.7 | 14.5 | -3.2 | 24.9 | 13.8 | -2.2 |
| Srikakulam | 33.5 | 20.7 | -2.6 | 19.5 | 13.0 | -1.3 |
| Tirupati | 36.0 | 17.5 | -3.7 | 23.0 | 15.3 | -1.5 |
| Visakhapatnam | 36.7 | 22.3 | -2.9 | 21.4 | 11.4 | -2.0 |
| Vizianagaram | 45.1 | 26.4 | -3.7 | 31.8 | 12.0 | -4.0 |
| West Godavari | 37.9 | 19.7 | -3.6 | 23.1 | 13.4 | -1.9 |
| Y.S.R. | 38.8 | 15.0 | -4.7 | 30.2 | 13.6 | -3.3 |
| Muzaffarabad |  |  |  |  |  |  |
| Kolkata | 47.0 | 19.8 | -5.4 | 59.7 | 39.2 | -4.1 |
| Kupwara | 1.3 | 0.5 | -0.2 | 44.6 | 42.7 | -0.4 |
| Badgam | 1.7 | 0.5 | -0.2 | 40.0 | 35.0 | -1.0 |
| Belgaum | 34.4 | 35.1 | 0.2 | 22.3 | 36.4 | 2.8 |
| Punch | 5.5 | 2.1 | -0.7 | 29.0 | 34.2 | 1.0 |
| Kathua | 9.7 | 5.8 | -0.8 | 38.4 | 39.4 | 0.2 |
| Rajouri | 32.8 | 15.8 | -3.4 | 34.3 | 23.8 | -2.1 |
| Baramula | 2.1 | 1.3 | -0.1 | 40.2 | 30.0 | -2.0 |
| Bandipore | 1.2 | 0.6 | -0.1 | 50.9 | 33.7 | -3.4 |
| Srinagar | 1.4 | 0.7 | -0.1 | 38.3 | 31.5 | -1.3 |
| Ganderbal | 1.3 | 0.5 | -0.2 | 45.3 | 28.5 | -3.4 |
| Pulwama | 1.4 | 0.9 | -0.1 | 41.4 | 32.4 | -1.8 |
| Shupiyan | 1.2 | 0.6 | -0.1 | 41.7 | 37.5 | -0.8 |
| Anantnag | 1.5 | 0.5 | -0.2 | 43.7 | 37.4 | -1.3 |
| Kulgam | 1.2 | 0.6 | -0.1 | 42.5 | 28.1 | -2.9 |
| Doda | 11.8 | 5.7 | -1.2 | 40.3 | 31.2 | -1.8 |
| Ramban | 8.3 | 9.2 | 0.2 | 39.1 | 31.1 | -1.6 |
| Kishtwar | 10.0 | 7.5 | -0.5 | 38.9 | 34.8 | -0.8 |
| Udhampur | 23.9 | 23.1 | -0.1 | 43.9 | 39.6 | -0.9 |
| Reasi | 16.4 | 8.5 | -1.6 | 31.3 | 34.7 | 0.7 |
| Jammu | 28.1 | 19.9 | -1.7 | 24.6 | 20.8 | -0.8 |
| Samba | 29.6 | 24.3 | -1.1 | 33.7 | 32.3 | -0.3 |
| Chamba | 50.9 | 30.0 | -4.2 | 51.3 | 26.3 | -5.0 |
| Kangra | 50.7 | 26.1 | -4.9 | 40.2 | 23.6 | -3.3 |
| Lahul & Spiti | 51.0 | 45.0 | -1.2 | 40.9 | 32.1 | -1.8 |
| Kullu | 40.5 | 28.9 | -2.3 | 40.7 | 27.7 | -2.6 |
| Mandi | 39.8 | 25.6 | -2.9 | 42.6 | 23.1 | -3.9 |
| Hamirpur | 39.1 | 23.3 | -3.2 | 38.5 | 26.2 | -2.5 |
| Una | 29.0 | 21.0 | -1.6 | 36.7 | 24.5 | -2.5 |
| Bilaspur | 36.3 | 19.1 | -3.4 | 42.2 | 24.9 | -3.5 |
| Solan | 26.0 | 23.3 | -0.5 | 36.8 | 24.2 | -2.5 |
| Sirmaur | 33.8 | 18.2 | -3.1 | 36.0 | 20.6 | -3.1 |
| Shimla | 36.6 | 28.2 | -1.7 | 38.9 | 23.6 | -3.1 |
| Kinnaur | 56.1 | 47.4 | -1.7 | 49.7 | 40.5 | -1.8 |
| Yanam | 33.7 | 17.8 | -3.2 | 19.4 | 9.2 | -2.0 |
| Puducherry | 33.7 | 17.0 | -3.3 | 18.7 | 8.3 | -2.1 |
| Karaikal | 48.9 | 12.6 | -7.3 | 25.8 | 7.4 | -3.7 |
| Mahe | 41.5 | 14.5 | -5.4 | 22.2 | 8.0 | -2.8 |
| Kapurthala | 21.5 | 20.2 | -0.3 | 13.6 | 10.8 | -0.6 |
| Jalandhar | 29.4 | 17.2 | -2.4 | 17.2 | 11.7 | -1.1 |
| Hoshiarpur | 39.3 | 26.4 | -2.6 | 20.0 | 13.9 | -1.2 |
| Shahid Bhagat Singh Nagar | 24.5 | 29.0 | 0.9 | 13.3 | 18.3 | 1.0 |
| Fatehgarh Sahib | 28.4 | 24.9 | -0.7 | 15.2 | 16.0 | 0.2 |
| Ludhiana | 27.3 | 26.4 | -0.2 | 11.7 | 15.9 | 0.9 |
| Moga | 43.0 | 24.4 | -3.7 | 27.1 | 13.3 | -2.8 |
| Muktsar | 25.7 | 19.3 | -1.3 | 13.9 | 9.6 | -0.9 |
| Faridkot | 37.1 | 14.9 | -4.4 | 13.9 | 7.4 | -1.3 |
| Bathinda | 33.7 | 20.5 | -2.6 | 11.7 | 7.2 | -0.9 |
| Mansa | 27.1 | 17.2 | -2.0 | 16.9 | 8.9 | -1.6 |
| Patiala | 26.9 | 17.9 | -1.8 | 14.6 | 11.0 | -0.7 |
| Amritsar | 39.3 | 18.5 | -4.2 | 16.7 | 10.4 | -1.3 |
| Tarn Taran | 39.7 | 25.9 | -2.8 | 19.1 | 11.6 | -1.5 |
| Chandigarh | 39.4 | 15.8 | -4.7 | 22.5 | 9.4 | -2.6 |
| Uttarkashi | 34.9 | 23.2 | -2.3 | 43.0 | 27.9 | -3.0 |
| Chamoli | 37.2 | 30.9 | -1.3 | 45.7 | 28.7 | -3.4 |
| Rudraprayag | 45.4 | 29.0 | -3.3 | 44.5 | 26.7 | -3.6 |
| Tehri Garhwal | 37.2 | 24.5 | -2.5 | 40.8 | 25.9 | -3.0 |
| Dehradun | 36.2 | 15.6 | -4.1 | 39.3 | 19.2 | -4.0 |
| Garhwal | 34.1 | 26.6 | -1.5 | 47.0 | 31.7 | -3.1 |
| Pithoragarh | 45.2 | 26.0 | -3.8 | 52.2 | 26.6 | -5.1 |
| Bageshwar | 44.6 | 29.3 | -3.1 | 45.7 | 30.0 | -3.1 |
| Almora | 38.7 | 22.8 | -3.2 | 39.9 | 31.3 | -1.7 |
| Champawat | 38.7 | 26.7 | -2.4 | 52.3 | 27.4 | -5.0 |
| Nainital | 35.5 | 22.4 | -2.6 | 43.9 | 26.3 | -3.5 |
| Udham Singh Nagar | 37.1 | 18.4 | -3.7 | 52.7 | 25.2 | -5.5 |
| Hardwar | 21.7 | 14.8 | -1.4 | 36.1 | 24.8 | -2.3 |
| Panchkula | 26.5 | 16.1 | -2.1 | 29.5 | 15.4 | -2.8 |
| Ambala | 40.5 | 15.2 | -5.1 | 34.0 | 16.2 | -3.6 |
| Yamunanagar | 35.1 | 17.3 | -3.6 | 32.7 | 19.5 | -2.6 |
| Kurukshetra | 14.1 | 14.7 | 0.1 | 24.2 | 18.0 | -1.2 |
| Kaithal | 14.3 | 10.6 | -0.7 | 31.5 | 16.3 | -3.0 |
| Karnal | 20.7 | 18.3 | -0.5 | 28.3 | 20.4 | -1.6 |
| Panipat | 27.0 | 14.3 | -2.5 | 31.1 | 20.7 | -2.1 |
| Sonipat | 23.3 | 15.6 | -1.5 | 33.5 | 24.4 | -1.8 |
| Jind | 32.5 | 12.9 | -3.9 | 46.3 | 25.2 | -4.2 |
| Fatehabad | 22.6 | 10.7 | -2.4 | 36.6 | 17.0 | -3.9 |
| Sirsa | 24.6 | 9.3 | -3.1 | 34.5 | 17.5 | -3.4 |
| Hisar | 18.3 | 11.7 | -1.3 | 37.8 | 26.4 | -2.3 |
| Rohtak | 23.6 | 14.3 | -1.9 | 44.8 | 22.6 | -4.4 |
| Jhajjar | 25.5 | 12.4 | -2.6 | 43.8 | 24.9 | -3.8 |
| Mahendragarh | 18.9 | 10.3 | -1.7 | 30.9 | 20.8 | -2.0 |
| Rewari | 26.2 | 16.5 | -1.9 | 42.8 | 24.8 | -3.6 |
| Gurgaon | 28.7 | 16.1 | -2.5 | 39.7 | 26.8 | -2.6 |
| Mewat | 6.9 | 5.4 | -0.3 | 55.9 | 27.4 | -5.7 |
| Faridabad | 25.6 | 13.1 | -2.5 | 27.1 | 18.0 | -1.8 |
| Palwal | 16.6 | 9.8 | -1.4 | 30.9 | 20.6 | -2.1 |
| Ganganagar | 17.2 | 12.0 | -1.1 | 29.5 | 23.9 | -1.1 |
| Hanumangarh | 13.0 | 10.1 | -0.6 | 45.5 | 28.2 | -3.5 |
| Bikaner | 18.1 | 7.1 | -2.2 | 48.3 | 32.9 | -3.1 |
| Churu | 15.3 | 7.2 | -1.6 | 48.1 | 32.1 | -3.2 |
| Jhunjhunun | 13.8 | 8.7 | -1.0 | 46.9 | 29.8 | -3.4 |
| Alwar | 14.2 | 10.5 | -0.7 | 50.7 | 34.1 | -3.3 |
| Bharatpur | 14.3 | 11.7 | -0.5 | 59.9 | 39.7 | -4.1 |
| Dhaulpur | 12.0 | 9.4 | -0.5 | 53.1 | 40.7 | -2.5 |
| Karauli | 9.5 | 5.9 | -0.7 | 55.5 | 42.7 | -2.6 |
| Mirpur | 10.8 | 6.6 | -0.8 | 60.6 | 41.2 | -3.9 |
| Rupnagar | 9.3 | 6.2 | -0.6 | 42.3 | 35.5 | -1.4 |
| Sahibzada Ajit Singh Nagar | 18.2 | 6.8 | -2.3 | 39.2 | 26.0 | -2.7 |
| Sangrur | 18.1 | 7.2 | -2.2 | 43.0 | 27.0 | -3.2 |
| Barnala | 12.7 | 6.0 | -1.3 | 58.0 | 35.1 | -4.6 |
| Fazilka | 10.6 | 6.0 | -0.9 | 44.3 | 36.9 | -1.5 |
| Firozpur | 18.4 | 8.6 | -2.0 | 42.8 | 37.9 | -1.0 |
| Gurdaspur | 9.4 | 9.1 | -0.1 | 46.7 | 40.8 | -1.2 |
| Pathankot | 7.6 | 9.6 | 0.4 | 39.1 | 37.9 | -0.2 |
| Bhiwani | 12.4 | 10.9 | -0.3 | 40.2 | 33.8 | -1.3 |
| Charkhi Dadri | 16.1 | 7.9 | -1.6 | 45.4 | 32.9 | -2.5 |
| Central | 24.9 | 10.3 | -2.9 | 46.4 | 32.0 | -2.9 |
| East | 14.4 | 10.3 | -0.8 | 51.0 | 39.1 | -2.4 |
| New Delhi | 11.7 | 11.2 | -0.1 | 51.8 | 50.6 | -0.2 |
| North | 12.5 | 9.5 | -0.6 | 43.0 | 36.0 | -1.4 |
| North East | 20.3 | 9.1 | -2.2 | 50.6 | 34.9 | -3.1 |
| North West | 17.0 | 9.2 | -1.6 | 41.2 | 34.6 | -1.3 |
| Shahdara | 17.9 | 15.1 | -0.6 | 37.6 | 33.9 | -0.8 |
| South | 13.5 | 9.7 | -0.7 | 54.5 | 38.7 | -3.2 |
| South East | 18.8 | 10.7 | -1.6 | 46.9 | 40.0 | -1.4 |
| South West | 16.7 | 15.9 | -0.2 | 67.3 | 52.8 | -2.9 |
| West | 14.4 | 13.6 | -0.2 | 54.0 | 49.6 | -0.9 |
| Sawai Madhopur | 11.2 | 11.2 | 0.0 | 38.1 | 35.0 | -0.6 |
| Dausa | 13.0 | 16.2 | 0.6 | 46.4 | 32.7 | -2.7 |
| Jaipur | 13.0 | 11.4 | -0.3 | 40.6 | 23.9 | -3.3 |
| Sikar | 16.6 | 13.9 | -0.5 | 49.8 | 29.1 | -4.1 |
| Nagaur | 22.8 | 12.7 | -2.0 | 51.5 | 30.9 | -4.1 |
| Jodhpur | 23.5 | 12.7 | -2.2 | 44.7 | 31.2 | -2.7 |
| Jaisalmer | 12.6 | 11.2 | -0.3 | 37.8 | 19.5 | -3.7 |
| Barmer | 27.9 | 10.6 | -3.5 | 50.7 | 22.6 | -5.6 |
| Jalor | 22.1 | 15.4 | -1.3 | 42.4 | 25.6 | -3.4 |
| Sirohi | 21.0 | 14.5 | -1.3 | 57.3 | 27.9 | -5.9 |
| Pali | 17.8 | 17.1 | -0.2 | 56.8 | 32.7 | -4.8 |
| Ajmer | 17.7 | 12.0 | -1.1 | 54.4 | 37.8 | -3.3 |
| Tonk | 15.9 | 12.5 | -0.7 | 48.2 | 38.6 | -1.9 |
| Bundi | 17.6 | 18.5 | 0.2 | 51.5 | 40.4 | -2.2 |
| Bhilwara | 21.4 | 16.1 | -1.1 | 49.6 | 40.3 | -1.9 |
| Rajsamand | 14.0 | 14.6 | 0.1 | 46.5 | 35.0 | -2.3 |
| Dungarpur | 16.5 | 16.1 | -0.1 | 53.8 | 35.1 | -3.7 |
| Banswara | 19.0 | 13.9 | -1.0 | 62.4 | 33.8 | -5.7 |
| Chittaurgarh | 11.6 | 19.2 | 1.5 | 61.4 | 43.8 | -3.5 |
| Kota | 23.1 | 13.0 | -2.0 | 65.7 | 55.8 | -2.0 |
| Baran | 27.9 | 13.7 | -2.8 | 61.8 | 46.6 | -3.0 |
| Jhalawar | 25.9 | 15.3 | -2.1 | 58.9 | 44.5 | -2.9 |
| Udaipur | 18.3 | 13.1 | -1.0 | 49.6 | 36.3 | -2.7 |
| Pratapgarh | 16.4 | 18.0 | 0.3 | 47.6 | 44.1 | -0.7 |
| Saharanpur | 19.6 | 13.7 | -1.2 | 60.1 | 44.3 | -3.1 |
| Bijnor | 26.2 | 17.1 | -1.8 | 54.8 | 40.0 | -2.9 |
| Rampur | 15.1 | 10.4 | -0.9 | 53.5 | 41.8 | -2.3 |
| Jyotiba Phule Nagar | 29.6 | 16.6 | -2.6 | 58.2 | 45.9 | -2.5 |
| Meerut | 28.0 | 15.3 | -2.5 | 56.3 | 42.8 | -2.7 |
| Baghpat | 13.0 | 11.4 | -0.3 | 61.9 | 44.0 | -3.6 |
| Gautam Buddha Nagar | 19.5 | 9.0 | -2.1 | 56.3 | 43.4 | -2.6 |
| Bulandshahr | 19.2 | 17.1 | -0.4 | 58.0 | 51.4 | -1.3 |
| Aligarh | 18.8 | 13.6 | -1.1 | 64.3 | 58.5 | -1.1 |
| Mahamaya Nagar | 27.6 | 11.1 | -3.3 | 61.2 | 55.6 | -1.1 |
| Mathura | 24.2 | 12.5 | -2.3 | 65.2 | 56.7 | -1.7 |
| Agra | 30.2 | 13.8 | -3.3 | 72.6 | 54.4 | -3.6 |
| Firozabad | 33.1 | 16.2 | -3.4 | 63.8 | 54.2 | -1.9 |
| Mainpuri | 15.9 | 7.4 | -1.7 | 51.5 | 31.0 | -4.1 |
| Bareilly | 20.6 | 12.0 | -1.7 | 56.6 | 46.2 | -2.1 |
| Pilibhit | 26.3 | 11.2 | -3.0 | 53.2 | 37.4 | -3.2 |
| Shahjahanpur | 24.9 | 11.2 | -2.7 | 62.1 | 45.0 | -3.4 |
| Sitapur | 19.3 | 9.0 | -2.0 | 59.3 | 35.1 | -4.8 |
| Hardoi | 17.1 | 14.2 | -0.6 | 61.1 | 43.5 | -3.5 |
| Unnao | 22.0 | 12.2 | -2.0 | 62.4 | 54.1 | -1.7 |
| Lucknow | 14.3 | 8.9 | -1.1 | 72.9 | 60.6 | -2.5 |
| Farrukhabad | 63.2 | 37.7 | -5.1 | 69.9 | 45.4 | -4.9 |
| Kannauj | 15.6 | 9.1 | -1.3 | 56.0 | 45.3 | -2.1 |
| Etawah | 16.9 | 10.0 | -1.4 | 58.6 | 44.9 | -2.7 |
| Auraiya | 16.2 | 14.3 | -0.4 | 46.9 | 42.1 | -1.0 |
| Kanpur Dehat | 27.7 | 12.1 | -3.1 | 61.9 | 41.9 | -4.0 |
| Kanpur Nagar | 25.4 | 13.8 | -2.3 | 56.1 | 39.4 | -3.3 |
| Jalaun | 29.4 | 15.0 | -2.9 | 48.0 | 35.5 | -2.5 |
| Jhansi | 31.4 | 14.7 | -3.3 | 58.2 | 36.8 | -4.3 |
| Lalitpur | 19.6 | 10.8 | -1.8 | 39.8 | 28.6 | -2.2 |
| Hamirpur | 13.5 | 11.6 | -0.4 | 47.5 | 31.1 | -3.3 |
| Mahoba | 13.7 | 10.7 | -0.6 | 43.1 | 29.2 | -2.8 |
| Banda | 16.3 | 8.3 | -1.6 | 39.6 | 27.8 | -2.4 |
| Chitrakoot | 20.1 | 11.2 | -1.8 | 51.3 | 32.6 | -3.7 |
| Fatehpur | 15.0 | 8.4 | -1.3 | 41.1 | 26.2 | -3.0 |
| Pratapgarh | 32.4 | 16.3 | -3.2 | 54.2 | 37.4 | -3.4 |
| Kaushambi | 24.9 | 11.2 | -2.7 | 51.2 | 29.7 | -4.3 |
| Allahabad | 17.8 | 6.2 | -2.3 | 54.5 | 27.1 | -5.5 |
| Bara Banki | 25.9 | 11.8 | -2.8 | 52.8 | 35.2 | -3.5 |
| Faizabad | 35.6 | 21.4 | -2.8 | 63.1 | 46.1 | -3.4 |
| Ambedkar Nagar | 29.3 | 13.7 | -3.1 | 49.5 | 38.4 | -2.2 |
| Bahraich | 12.2 | 16.0 | 0.8 | 55.4 | 38.8 | -3.3 |
| Shrawasti | 25.1 | 10.4 | -2.9 | 49.5 | 38.3 | -2.2 |
| Gonda | 26.6 | 13.8 | -2.6 | 53.0 | 45.0 | -1.6 |
| Siddharthnagar | 28.6 | 16.9 | -2.4 | 54.7 | 45.7 | -1.8 |
| Basti | 30.0 | 13.9 | -3.2 | 45.6 | 44.1 | -0.3 |
| Sant Kabir Nagar | 22.3 | 13.1 | -1.8 | 51.8 | 42.1 | -2.0 |
| Maharajganj | 22.4 | 9.1 | -2.7 | 51.7 | 44.7 | -1.4 |
| Gorakhpur | 22.5 | 10.0 | -2.5 | 53.1 | 46.4 | -1.3 |
| Kushinagar | 17.3 | 7.0 | -2.1 | 60.0 | 41.4 | -3.7 |
| Deoria | 27.0 | 12.7 | -2.9 | 60.2 | 39.9 | -4.1 |
| Azamgarh | 17.7 | 11.0 | -1.3 | 50.5 | 42.3 | -1.6 |
| Mau | 24.3 | 16.8 | -1.5 | 53.1 | 45.1 | -1.6 |
| Ballia | 26.1 | 10.8 | -3.1 | 49.0 | 45.7 | -0.7 |
| Jaunpur | 27.5 | 11.4 | -3.2 | 49.2 | 47.5 | -0.3 |
| Ghazipur | 27.6 | 11.1 | -3.3 | 47.5 | 42.4 | -1.0 |
| Chandauli | 19.7 | 11.7 | -1.6 | 38.8 | 36.7 | -0.4 |
| Varanasi | 20.4 | 11.1 | -1.9 | 40.3 | 36.3 | -0.8 |
| Sant Ravidas Nagar | 23.3 | 15.7 | -1.5 | 50.5 | 33.5 | -3.4 |
| Mirzapur | 38.0 | 16.8 | -4.2 | 59.9 | 35.8 | -4.8 |
| Sonbhadra | 26.0 | 11.1 | -3.0 | 49.0 | 42.1 | -1.4 |
| Etah | 31.5 | 14.0 | -3.5 | 52.7 | 42.5 | -2.0 |
| Kanshiram Nagar | 31.3 | 8.7 | -4.5 | 60.2 | 40.2 | -4.0 |
| Balrampur | 19.7 | 19.4 | -0.1 | 44.9 | 41.6 | -0.7 |
| Amethi | 22.1 | 19.1 | -0.6 | 50.2 | 42.5 | -1.6 |
| Budaun | 31.0 | 14.3 | -3.3 | 49.8 | 36.8 | -2.6 |
| Ghaziabad | 39.5 | 19.8 | -3.9 | 57.4 | 40.0 | -3.5 |
| Hapur | 41.7 | 22.5 | -3.8 | 55.2 | 39.4 | -3.1 |
| Moradabad | 36.8 | 20.9 | -3.2 | 48.0 | 41.8 | -1.2 |
| Muzaffarnagar | 41.5 | 20.4 | -4.2 | 52.8 | 33.2 | -3.9 |
| Rae Bareli | 23.8 | 17.9 | -1.2 | 50.2 | 37.6 | -2.5 |
| Sambhal | 25.4 | 11.7 | -2.7 | 50.0 | 34.4 | -3.1 |
| Shamli | 31.3 | 14.1 | -3.4 | 49.7 | 42.5 | -1.4 |
| Sultanpur | 26.3 | 18.3 | -1.6 | 53.6 | 34.1 | -3.9 |
| Kheri | 32.1 | 27.4 | -1.0 | 55.8 | 43.7 | -2.4 |
| Pashchim Champaran | 47.2 | 26.8 | -4.1 | 51.9 | 39.4 | -2.5 |
| Purba Champaran | 22.4 | 23.4 | 0.2 | 42.9 | 46.1 | 0.6 |
| Sheohar | 43.2 | 26.3 | -3.4 | 56.3 | 40.5 | -3.2 |
| Sitamarhi | 35.5 | 25.1 | -2.1 | 52.2 | 40.3 | -2.4 |
| Madhubani | 60.1 | 42.4 | -3.5 | 31.7 | 33.9 | 0.4 |
| Supaul | 54.6 | 45.3 | -1.9 | 38.8 | 39.6 | 0.2 |
| Araria | 56.2 | 34.2 | -4.4 | 39.3 | 41.3 | 0.4 |
| Kishanganj | 49.8 | 32.7 | -3.4 | 44.2 | 37.6 | -1.3 |
| Purnia | 40.7 | 39.3 | -0.3 | 34.8 | 23.3 | -2.3 |
| Katihar | 46.2 | 41.9 | -0.9 | 55.4 | 35.4 | -4.0 |
| Madhepura | 55.2 | 42.3 | -2.6 | 59.7 | 43.3 | -3.3 |
| Saharsa | 43.4 | 43.2 | 0.0 | 49.4 | 41.3 | -1.6 |
| Darbhanga | 46.7 | 60.3 | 2.7 | 53.6 | 51.4 | -0.4 |
| Muzaffarpur | 71.3 | 56.2 | -3.0 | 57.5 | 55.6 | -0.4 |
| Gopalganj | 69.3 | 56.8 | -2.5 | 71.8 | 54.6 | -3.4 |
| Siwan | 54.7 | 45.1 | -1.9 | 58.6 | 44.9 | -2.7 |
| Saran | 68.2 | 59.9 | -1.7 | 56.5 | 52.3 | -0.8 |
| Vaishali | 71.9 | 47.6 | -4.9 | 66.0 | 49.0 | -3.4 |
| Samastipur | 70.0 | 62.8 | -1.4 | 66.0 | 57.0 | -1.8 |
| Begusarai | 35.4 | 30.8 | -0.9 | 56.9 | 60.6 | 0.7 |
| Khagaria | 49.3 | 27.1 | -4.4 | 78.5 | 56.8 | -4.3 |
| Bhagalpur | 33.9 | 26.9 | -1.4 | 78.1 | 61.6 | -3.3 |
| Banka | 43.5 | 34.0 | -1.9 | 76.4 | 59.8 | -3.3 |
| Munger | 41.0 | 29.6 | -2.3 | 74.6 | 49.0 | -5.1 |
| Lakhisarai | 37.9 | 28.9 | -1.8 | 66.2 | 51.2 | -3.0 |
| Sheikhpura | 28.5 | 20.8 | -1.6 | 65.8 | 56.9 | -1.8 |
| Nalanda | 33.8 | 31.9 | -0.4 | 76.5 | 60.9 | -3.1 |
| Patna | 31.3 | 20.0 | -2.3 | 63.8 | 52.5 | -2.3 |
| Bhojpur | 36.2 | 31.3 | -1.0 | 73.1 | 55.6 | -3.5 |
| Buxer | 51.4 | 30.3 | -4.2 | 80.6 | 58.7 | -4.4 |
| Kaimur (Bhabua) | 55.7 | 37.8 | -3.6 | 81.7 | 64.5 | -3.4 |
| Rohtas | 46.9 | 39.0 | -1.6 | 68.8 | 55.7 | -2.6 |
| Gaya | 53.6 | 45.1 | -1.7 | 81.1 | 75.5 | -1.1 |
| Nawada | 47.9 | 46.9 | -0.2 | 70.8 | 60.9 | -2.0 |
| Jamui | 35.8 | 32.8 | -0.6 | 69.8 | 57.3 | -2.5 |
| Jehanabad | 55.8 | 39.5 | -3.2 | 64.6 | 52.7 | -2.4 |
| Arwal | 61.0 | 36.7 | -4.9 | 72.5 | 52.8 | -4.0 |
| North District | 64.7 | 42.8 | -4.4 | 83.6 | 72.5 | -2.2 |
| West District | 54.3 | 46.3 | -1.6 | 73.9 | 67.6 | -1.3 |
| South District | 48.0 | 28.9 | -3.8 | 80.1 | 79.9 | 0.0 |
| East District | 46.6 | 25.2 | -4.3 | 85.6 | 74.6 | -2.2 |
| Tawang | 54.3 | 30.0 | -4.9 | 83.0 | 70.3 | -2.5 |
| West Kameng | 42.9 | 26.2 | -3.3 | 80.9 | 73.7 | -1.4 |
| East Kameng | 48.9 | 28.5 | -4.1 | 85.3 | 76.0 | -1.9 |
| Papum Pare | 44.9 | 22.6 | -4.5 | 79.2 | 73.3 | -1.2 |
| Upper Subansiri | 39.7 | 31.2 | -1.7 | 76.4 | 75.4 | -0.2 |
| Upper Siang | 67.4 | 41.7 | -5.2 | 79.9 | 56.7 | -4.6 |
| Changlang | 51.2 | 32.8 | -3.7 | 63.5 | 47.6 | -3.2 |
| Lower Subansiri | 41.3 | 30.9 | -2.1 | 73.5 | 57.4 | -3.2 |
| Dibang Valley | 36.4 | 23.1 | -2.6 | 72.1 | 51.2 | -4.2 |
| Lower Dibang Valley | 31.5 | 31.5 | 0.0 | 59.5 | 52.4 | -1.4 |
| Anjaw | 23.6 | 16.3 | -1.5 | 58.7 | 48.2 | -2.1 |
| East Siang | 11.5 | 8.0 | -0.7 | 60.2 | 43.5 | -3.3 |
| Kra Daadi | 38.1 | 14.7 | -4.7 | 73.2 | 52.5 | -4.1 |
| Kurung Kumey | 59.0 | 37.7 | -4.3 | 65.5 | 49.8 | -3.1 |
| Lohit | 59.4 | 55.5 | -0.8 | 70.4 | 56.2 | -2.9 |
| Langding | 48.2 | 43.4 | -1.0 | 74.5 | 48.0 | -5.3 |
| Namsai | 65.0 | 49.6 | -3.1 | 67.5 | 54.1 | -2.7 |
| Siang | 34.2 | 36.1 | 0.4 | 53.7 | 46.7 | -1.4 |
| Tirap | 71.1 | 52.9 | -3.6 | 79.0 | 50.6 | -5.7 |
| West Siang | 35.5 | 14.1 | -4.3 | 78.4 | 46.8 | -6.3 |
| Mon | 31.6 | 6.5 | -5.0 | 75.4 | 49.7 | -5.1 |
| Mokokchung | 29.4 | 10.0 | -3.9 | 84.1 | 54.4 | -5.9 |
| Zunheboto | 14.7 | 13.7 | -0.2 | 43.9 | 39.1 | -1.0 |
| Wokha | 36.4 | 29.9 | -1.3 | 54.7 | 50.4 | -0.9 |
| Dimapur | 25.5 | 18.3 | -1.4 | 53.2 | 41.1 | -2.4 |
| Phek | 38.0 | 23.4 | -2.9 | 55.1 | 36.4 | -3.7 |
| Tuensang | 16.8 | 13.4 | -0.7 | 61.6 | 36.8 | -4.9 |
| Longleng | 38.5 | 31.7 | -1.4 | 49.3 | 54.6 | 1.1 |
| Kiphire | 13.7 | 8.7 | -1.0 | 66.2 | 41.5 | -4.9 |
| Kohima | 54.0 | 33.3 | -4.2 | 54.6 | 47.8 | -1.4 |
| Peren | 47.3 | 30.8 | -3.3 | 48.3 | 45.4 | -0.6 |
| Senapati | 32.3 | 29.8 | -0.5 | 60.8 | 48.4 | -2.5 |
| Tamenglong | 19.4 | 16.2 | -0.6 | 58.9 | 49.3 | -1.9 |
| Churachandpur | 19.9 | 16.4 | -0.7 | 59.6 | 42.9 | -3.3 |
| Bishnupur | 45.9 | 21.8 | -4.8 | 69.9 | 41.1 | -5.8 |
| Thoubal | 27.8 | 14.1 | -2.7 | 61.5 | 39.9 | -4.3 |
| Imphal West | 12.4 | 10.4 | -0.4 | 59.5 | 39.9 | -3.9 |
| Imphal East | 33.0 | 17.9 | -3.0 | 62.0 | 45.2 | -3.4 |
| Ukhrul | 18.2 | 11.7 | -1.3 | 55.3 | 41.8 | -2.7 |
| Chandel | 33.8 | 19.3 | -2.9 | 62.7 | 47.1 | -3.1 |
| Mamit | 34.3 | 24.0 | -2.1 | 55.6 | 50.3 | -1.1 |
| Kolasib | 23.9 | 15.8 | -1.6 | 62.2 | 42.0 | -4.0 |
| Aizawl | 22.2 | 17.6 | -0.9 | 69.2 | 45.6 | -4.7 |
| Champhai | 18.9 | 17.7 | -0.2 | 60.2 | 46.1 | -2.8 |
| Serchhip | 29.4 | 19.6 | -2.0 | 55.6 | 44.0 | -2.3 |
| Lunglei | 21.7 | 15.7 | -1.2 | 49.7 | 35.5 | -2.8 |
| Lawngtlai | 22.9 | 24.7 | 0.3 | 41.8 | 39.2 | -0.5 |
| Saiha | 51.0 | 31.4 | -3.9 | 45.5 | 38.3 | -1.5 |
| Dhalai | 37.7 | 23.7 | -2.8 | 43.3 | 28.0 | -3.1 |
| Gomati | 29.3 | 27.1 | -0.4 | 40.6 | 36.4 | -0.8 |
| Khowai | 44.4 | 27.1 | -3.5 | 55.2 | 45.6 | -1.9 |
| North Tripura | 36.5 | 33.5 | -0.6 | 45.5 | 40.4 | -1.0 |
| South Tripura | 35.1 | 32.2 | -0.6 | 56.6 | 40.5 | -3.2 |
| Unakoti | 45.8 | 32.6 | -2.7 | 66.8 | 44.0 | -4.6 |
| West Tripura | 30.0 | 24.3 | -1.2 | 44.1 | 39.6 | -0.9 |
| Sepahijala | 40.8 | 27.0 | -2.8 | 52.6 | 39.2 | -2.7 |
| South Garo Hills | 60.3 | 32.7 | -5.5 | 63.6 | 43.3 | -4.1 |
| Ribhoi | 26.4 | 25.1 | -0.2 | 41.9 | 34.7 | -1.4 |
| East Khasi Hills | 33.3 | 28.3 | -1.0 | 44.8 | 36.9 | -1.6 |
| East Garo Hills | 39.7 | 41.6 | 0.4 | 34.6 | 45.4 | 2.2 |
| East Jaintia Hills | 36.8 | 29.6 | -1.4 | 43.8 | 33.3 | -2.1 |
| North Garo Hills | 43.1 | 34.5 | -1.7 | 52.0 | 43.4 | -1.7 |
| South West Garo Hills | 39.9 | 41.1 | 0.2 | 55.4 | 49.8 | -1.1 |
| South West Khasi Hills | 35.4 | 30.1 | -1.1 | 50.4 | 45.4 | -1.0 |
| West Garo Hills | 52.1 | 33.0 | -3.8 | 50.4 | 40.1 | -2.1 |
| West Jaintia Hills | 40.4 | 51.4 | 2.2 | 40.0 | 50.0 | 2.0 |
| West Khasi Hills | 45.8 | 46.9 | 0.2 | 58.5 | 46.2 | -2.5 |
| Kokrajhar | 49.4 | 50.2 | 0.2 | 46.8 | 49.6 | 0.5 |
| Goalpara | 53.2 | 53.2 | 0.0 | 58.2 | 51.7 | -1.3 |
| Barpeta | 51.7 | 34.7 | -3.4 | 54.8 | 34.2 | -4.1 |
| Morigaon | 39.3 | 27.7 | -2.3 | 52.5 | 35.4 | -3.4 |
| Lakhimpur | 44.5 | 29.1 | -3.1 | 60.0 | 40.7 | -3.9 |
| Dhemaji | 45.2 | 39.6 | -1.1 | 55.7 | 46.5 | -1.8 |
| Tinsukia | 46.3 | 43.6 | -0.5 | 59.1 | 55.6 | -0.7 |
| Dibrugarh | 54.6 | 44.3 | -2.1 | 66.3 | 47.3 | -3.8 |
| Golaghat | 43.9 | 45.6 | 0.3 | 66.9 | 62.4 | -0.9 |
| Dima Hasao | 43.0 | 45.0 | 0.4 | 55.3 | 57.8 | 0.5 |
| Cachar | 35.2 | 21.0 | -2.8 | 55.9 | 37.3 | -3.7 |
| Karimganj | 30.9 | 22.1 | -1.7 | 52.3 | 52.6 | 0.1 |
| Hailakandi | 23.7 | 23.9 | 0.0 | 51.6 | 47.3 | -0.9 |
| Bongaigaon | 31.1 | 22.2 | -1.8 | 53.5 | 44.2 | -1.9 |
| Chirang | 32.9 | 23.2 | -2.0 | 62.2 | 52.0 | -2.0 |
| Kamrup | 49.4 | 30.7 | -3.7 | 58.1 | 48.3 | -2.0 |
| Kamrup Metropolitan | 44.1 | 34.7 | -1.9 | 54.9 | 51.8 | -0.6 |
| Nalbari | 44.4 | 23.7 | -4.1 | 61.7 | 47.2 | -2.9 |
| Baksa | 27.7 | 22.2 | -1.1 | 52.6 | 39.2 | -2.7 |
| Darrang | 38.5 | 21.9 | -3.3 | 52.5 | 43.5 | -1.8 |
| Udalguri | 29.9 | 21.8 | -1.6 | 54.0 | 36.1 | -3.6 |
| Biswanath | 27.0 | 26.9 | 0.0 | 30.0 | 31.3 | 0.3 |
| Charaideo | 49.0 | 29.2 | -4.0 | 54.8 | 44.7 | -2.0 |
| Dhubri | 38.4 | 24.3 | -2.8 | 52.0 | 37.0 | -3.0 |
| Hojai | 42.0 | 20.9 | -4.2 | 56.9 | 30.7 | -5.2 |
| Jorhat | 40.7 | 24.2 | -3.3 | 54.1 | 35.3 | -3.8 |
| Karbi Anglong | 48.8 | 31.3 | -3.5 | 59.1 | 41.1 | -3.6 |
| Majuli | 38.1 | 29.4 | -1.8 | 54.7 | 44.5 | -2.1 |
| Nagaon | 30.8 | 28.8 | -0.4 | 53.7 | 41.2 | -2.5 |
| Sivasagar | 44.8 | 33.5 | -2.3 | 62.5 | 56.2 | -1.3 |
| Sonitpur | 40.0 | 32.2 | -1.6 | 57.7 | 51.2 | -1.3 |
| South Salmara-Mankachar | 58.6 | 40.2 | -3.7 | 72.3 | 50.9 | -4.3 |
| Karbi Anglong West | 53.9 | 34.9 | -3.8 | 62.8 | 38.5 | -4.9 |
| Darjiling | 55.7 | 46.1 | -1.9 | 55.9 | 49.5 | -1.3 |
| Jalpaiguri | 54.4 | 29.6 | -5.0 | 60.6 | 36.9 | -4.7 |
| Koch Bihar | 51.6 | 30.4 | -4.2 | 48.3 | 37.0 | -2.3 |
| Uttar Dinajpur | 43.6 | 27.2 | -3.3 | 44.5 | 33.7 | -2.2 |
| Dakshin Dinajpur | 40.5 | 25.2 | -3.1 | 47.1 | 35.6 | -2.3 |
| Maldah | 62.9 | 32.7 | -6.1 | 63.1 | 43.1 | -4.0 |
| Murshidabad | 32.7 | 25.7 | -1.4 | 49.5 | 35.3 | -2.8 |
| Birbhum | 50.8 | 30.9 | -4.0 | 58.1 | 35.0 | -4.6 |
| Nadia | 54.9 | 28.0 | -5.4 | 60.3 | 35.8 | -4.9 |
| North Twenty Four Parganas | 64.9 | 46.4 | -3.7 | 59.6 | 47.4 | -2.4 |
| Hugli | 57.7 | 54.9 | -0.6 | 55.2 | 56.7 | 0.3 |
| Bankura | 21.5 | 14.2 | -1.5 | 68.8 | 49.2 | -3.9 |
| Puruliya | 13.7 | 6.1 | -1.5 | 49.5 | 33.2 | -3.3 |
| South Twenty Four Parganas | 16.7 | 5.9 | -2.2 | 49.4 | 30.8 | -3.7 |
| Paschim Medinipur | 30.3 | 9.6 | -4.1 | 55.5 | 25.0 | -6.1 |
| Purba Medinipur | 14.6 | 8.5 | -1.2 | 49.2 | 37.7 | -2.3 |
| Paschim Barddhaman | 22.3 | 11.5 | -2.2 | 62.5 | 37.4 | -5.0 |
| Purba Barddhaman | 30.4 | 15.3 | -3.0 | 59.3 | 49.9 | -1.9 |
| Haora | 23.7 | 17.2 | -1.3 | 67.8 | 57.1 | -2.1 |
| Garhwa | 29.1 | 13.6 | -3.1 | 68.6 | 50.8 | -3.5 |
| Chatra | 24.4 | 15.4 | -1.8 | 67.0 | 47.1 | -4.0 |
| Kodarma | 25.2 | 14.4 | -2.2 | 66.6 | 51.1 | -3.1 |
| Giridih | 35.4 | 25.7 | -1.9 | 64.0 | 52.8 | -2.2 |
| Deoghar | 35.7 | 19.6 | -3.2 | 62.7 | 49.4 | -2.7 |
| Godda | 45.2 | 29.8 | -3.1 | 65.2 | 53.3 | -2.4 |
| Sahibganj | 24.9 | 8.2 | -3.3 | 51.3 | 26.2 | -5.0 |
| Pakur | 24.7 | 10.7 | -2.8 | 59.9 | 31.3 | -5.7 |
| Dhanbad | 26.7 | 12.7 | -2.8 | 60.5 | 29.1 | -6.3 |
| Bokaro | 23.8 | 13.4 | -2.1 | 47.3 | 33.7 | -2.7 |
| Lohardaga | 30.8 | 13.3 | -3.5 | 60.8 | 34.4 | -5.3 |
| Purbi Singhbhum | 34.1 | 23.0 | -2.2 | 60.3 | 40.0 | -4.0 |
| Palamu | 27.6 | 12.5 | -3.0 | 52.9 | 31.6 | -4.3 |
| Latehar | 26.4 | 14.1 | -2.5 | 60.1 | 38.3 | -4.4 |
| Hazaribagh | 31.9 | 18.1 | -2.8 | 60.3 | 38.0 | -4.5 |
| Ramgarh | 16.6 | 7.5 | -1.8 | 51.5 | 43.1 | -1.7 |
| Dumka | 21.9 | 12.6 | -1.8 | 72.0 | 47.9 | -4.8 |
| Jamtara | 18.7 | 11.5 | -1.4 | 53.8 | 28.3 | -5.1 |
| Ranchi | 29.9 | 9.6 | -4.1 | 60.3 | 39.9 | -4.1 |
| Khunti | 20.1 | 12.2 | -1.6 | 66.9 | 45.8 | -4.2 |
| Gumla | 36.3 | 16.0 | -4.1 | 63.8 | 41.2 | -4.5 |
| Simdega | 37.1 | 14.7 | -4.5 | 57.1 | 45.5 | -2.3 |
| Pashchimi Singhbhum | 38.3 | 16.0 | -4.5 | 61.9 | 47.6 | -2.8 |
| Saraikela-Kharsawan | 38.5 | 19.0 | -3.9 | 66.1 | 53.1 | -2.6 |
| Bargarh | 37.3 | 20.9 | -3.3 | 58.3 | 49.7 | -1.7 |
| Jharsuguda | 18.1 | 14.9 | -0.6 | 59.1 | 50.7 | -1.7 |
| Sambalpur | 39.3 | 32.3 | -1.4 | 68.7 | 54.5 | -2.8 |
| Debagarh | 35.0 | 23.1 | -2.4 | 69.2 | 51.4 | -3.6 |
| Sundargarh | 25.7 | 17.5 | -1.6 | 62.5 | 43.1 | -3.9 |
| Kendujhar | 34.9 | 20.3 | -2.9 | 63.1 | 51.6 | -2.3 |
| Mayurbhanj | 39.8 | 22.0 | -3.6 | 65.6 | 44.5 | -4.2 |
| Baleshwar | 26.7 | 12.2 | -2.9 | 60.2 | 35.7 | -4.9 |
| Bhadrak | 30.5 | 11.9 | -3.7 | 67.5 | 41.1 | -5.3 |
| Kendrapara | 38.2 | 25.1 | -2.6 | 61.3 | 40.4 | -4.2 |
| Cuttack | 43.7 | 35.1 | -1.7 | 66.4 | 48.5 | -3.6 |
| Jajapur | 32.9 | 24.4 | -1.7 | 58.1 | 39.7 | -3.7 |
| Dhenkanal | 47.7 | 16.9 | -6.2 | 65.3 | 30.3 | -7.0 |
| Anugul | 71.5 | 30.8 | -8.1 | 65.9 | 40.7 | -5.0 |
| Nayagarh | 22.8 | 14.5 | -1.7 | 64.9 | 40.8 | -4.8 |
| Khordha | 25.2 | 12.0 | -2.7 | 50.6 | 32.8 | -3.6 |
| Puri | 16.0 | 2.6 | -2.7 | 54.4 | 36.2 | -3.6 |
| Ganjam | 3.9 | 3.9 | 0.0 | 51.4 | 34.2 | -3.4 |
| Gajapati | 6.1 | 1.9 | -0.8 | 50.1 | 34.3 | -3.2 |
| Kandhamal | 6.1 | 2.9 | -0.7 | 45.4 | 31.7 | -2.7 |
| Baudh | 13.6 | 3.7 | -2.0 | 51.2 | 29.5 | -4.3 |
| Subarnapur | 6.7 | 4.5 | -0.4 | 70.5 | 59.4 | -2.2 |
| Balangir | 8.1 | 3.1 | -1.0 | 50.7 | 29.6 | -4.2 |
| Nuapada | 14.9 | 10.3 | -0.9 | 57.1 | 50.8 | -1.3 |
| Kalahandi | 13.1 | 7.5 | -1.1 | 52.7 | 45.7 | -1.4 |
| Rayagada | 13.8 | 5.1 | -1.7 | 48.0 | 31.3 | -3.3 |
| Nabarangapur | 28.3 | 11.2 | -3.4 | 58.0 | 39.0 | -3.8 |
| Koraput | 26.3 | 8.7 | -3.5 | 47.7 | 24.5 | -4.6 |
| Malkangiri | 22.3 | 14.6 | -1.5 | 40.7 | 28.6 | -2.4 |
| Jagatsinghapur | 11.8 | 9.7 | -0.4 | 34.5 | 31.5 | -0.6 |
| Balrampur | 37.3 | 30.8 | -1.3 | 31.1 | 34.5 | 0.7 |
| Koriya | 34.0 | 24.0 | -2.0 | 40.1 | 37.6 | -0.5 |
| Jashpur | 19.6 | 10.4 | -1.8 | 47.5 | 37.2 | -2.1 |
| Raigarh | 20.0 | 9.1 | -2.2 | 38.1 | 30.3 | -1.6 |
| Korba | 20.3 | 10.3 | -2.0 | 42.5 | 32.5 | -2.0 |
| Janjgir-Champa | 18.3 | 13.2 | -1.0 | 50.3 | 39.0 | -2.3 |
| Kabeerdham | 10.3 | 11.4 | 0.2 | 40.3 | 37.3 | -0.6 |
| Rajnandgaon | 21.0 | 9.2 | -2.4 | 43.5 | 36.9 | -1.3 |
| Mahasamund | 21.0 | 15.0 | -1.2 | 42.2 | 34.2 | -1.6 |
| Dhamtari | 30.8 | 23.1 | -1.5 | 66.5 | 49.3 | -3.4 |
| Uttar Bastar Kanker | 30.3 | 17.5 | -2.6 | 52.7 | 36.6 | -3.2 |
| Narayanpur | 23.0 | 22.8 | 0.0 | 57.7 | 56.8 | -0.2 |
| Bijapur | 23.6 | 19.3 | -0.8 | 54.3 | 49.1 | -1.0 |
| Balod | 25.0 | 30.2 | 1.0 | 49.2 | 57.5 | 1.7 |
| Baloda Bazar | 23.1 | 19.8 | -0.7 | 49.0 | 45.0 | -0.8 |
| Bastar | 21.1 | 17.0 | -0.8 | 44.9 | 44.7 | 0.0 |
| Bemetara | 15.0 | 11.7 | -0.7 | 46.4 | 33.7 | -2.5 |
| Bilaspur | 14.3 | 8.9 | -1.1 | 34.5 | 27.3 | -1.5 |
| Dantewada | 15.2 | 10.4 | -1.0 | 41.7 | 32.7 | -1.8 |
| Durg | 14.9 | 10.4 | -0.9 | 36.3 | 30.4 | -1.2 |
| Gariaband | 10.2 | 10.8 | 0.1 | 33.3 | 28.9 | -0.9 |
| Kodagaon | 21.5 | 8.2 | -2.7 | 34.1 | 17.6 | -3.3 |
| Mungeli | 23.7 | 16.0 | -1.5 | 30.9 | 18.9 | -2.4 |
| Raipur | 27.9 | 16.9 | -2.2 | 33.0 | 24.7 | -1.7 |
| Sukma | 22.3 | 10.8 | -2.3 | 32.5 | 24.9 | -1.5 |
| Surguja | 13.3 | 7.6 | -1.1 | 31.1 | 28.4 | -0.5 |
| Surajpur | 15.1 | 7.6 | -1.5 | 40.7 | 30.2 | -2.1 |
| Sheopur | 18.2 | 12.5 | -1.1 | 37.5 | 29.9 | -1.5 |
| Morena | 18.8 | 10.9 | -1.6 | 45.4 | 32.3 | -2.6 |
| Bhind | 20.7 | 10.3 | -2.1 | 38.0 | 28.0 | -2.0 |
| Gwalior | 11.6 | 9.0 | -0.5 | 34.0 | 28.6 | -1.1 |
| Datia | 18.2 | 10.0 | -1.6 | 45.2 | 22.5 | -4.6 |
| Shivpuri | 8.4 | 6.6 | -0.4 | 31.7 | 21.3 | -2.1 |
| Tikamgarh | 19.6 | 7.2 | -2.5 | 30.2 | 18.2 | -2.4 |
| Chhatarpur | 13.6 | 6.6 | -1.4 | 25.5 | 22.5 | -0.6 |
| Panna | 33.1 | 7.8 | -5.1 | 35.3 | 21.2 | -2.8 |
| Sagar | 16.4 | 12.3 | -0.8 | 24.2 | 29.4 | 1.0 |
| Damoh | 37.7 | 12.9 | -5.0 | 35.0 | 29.1 | -1.2 |
| Satna | 25.0 | 14.9 | -2.0 | 39.6 | 27.0 | -2.5 |
| Rewa | 28.7 | 13.1 | -3.1 | 32.3 | 24.1 | -1.6 |
| Umaria | 25.2 | 8.9 | -3.3 | 42.4 | 29.7 | -2.5 |
| Neemuch | 25.6 | 11.4 | -2.8 | 33.2 | 29.8 | -0.7 |
| Mandsaur | 23.8 | 14.2 | -1.9 | 33.1 | 32.6 | -0.1 |
| Ratlam | 21.6 | 9.1 | -2.5 | 32.0 | 20.4 | -2.3 |
| Ujjain | 20.5 | 9.3 | -2.3 | 37.4 | 29.2 | -1.6 |
| Dewas | 18.1 | 12.0 | -1.2 | 20.6 | 22.3 | 0.4 |
| Dhar | 35.7 | 16.5 | -3.8 | 39.7 | 31.6 | -1.6 |
| Indore | 20.1 | 9.9 | -2.0 | 27.6 | 24.8 | -0.6 |
| Khargone (West Nimar) | 29.1 | 17.0 | -2.4 | 35.1 | 28.1 | -1.4 |
| Barwani | 31.4 | 20.0 | -2.3 | 45.1 | 27.1 | -3.6 |
| Rajgarh | 30.3 | 16.2 | -2.8 | 33.1 | 21.8 | -2.3 |
| Vidisha | 33.5 | 15.1 | -3.7 | 36.0 | 19.0 | -3.4 |
| Bhopal | 24.6 | 13.7 | -2.2 | 28.4 | 14.7 | -2.7 |
| Sehore | 32.2 | 20.4 | -2.4 | 34.0 | 21.3 | -2.5 |
| Raisen | 35.9 | 7.7 | -5.7 | 31.1 | 9.9 | -4.2 |
| Betul | 20.3 | 25.3 | 1.0 | 22.3 | 21.4 | -0.2 |
| Harda | 35.6 | 19.2 | -3.3 | 40.0 | 20.0 | -4.0 |
| Hoshangabad | 32.5 | 18.3 | -2.8 | 40.5 | 18.0 | -4.5 |
| Katni | 18.8 | 14.3 | -0.9 | 24.9 | 27.8 | 0.6 |
| Jabalpur | 26.1 | 16.2 | -2.0 | 36.6 | 27.4 | -1.8 |
| Narsimhapur | 14.6 | 10.6 | -0.8 | 29.4 | 13.8 | -3.1 |
| Dindori | 17.3 | 10.0 | -1.5 | 20.1 | 15.2 | -1.0 |
| Mandla | 21.1 | 14.3 | -1.4 | 32.0 | 18.8 | -2.6 |
| Chhindwara | 23.3 | 14.7 | -1.7 | 27.5 | 19.0 | -1.7 |
| Seoni | 32.8 | 30.5 | -0.5 | 18.3 | 17.1 | -0.2 |
| Balaghat | 57.8 | 36.3 | -4.3 | 23.2 | 12.4 | -2.2 |
| Guna | 43.7 | 20.6 | -4.6 | 28.7 | 19.7 | -1.8 |
| Ashoknagar | 37.1 | 10.3 | -5.4 | 23.1 | 7.5 | -3.1 |
| Shahdol | 16.2 | 6.7 | -1.9 | 16.5 | 8.8 | -1.5 |
| Anuppur | 29.0 | 19.9 | -1.8 | 25.2 | 16.2 | -1.8 |
| Singrauli | 36.2 | 20.7 | -3.1 | 24.4 | 12.0 | -2.5 |
| Jhabua | 50.0 | 18.3 | -6.3 | 24.9 | 11.7 | -2.6 |
| Alirajpur | 34.2 | 20.2 | -2.8 | 33.7 | 18.9 | -3.0 |
| Khandwa (East Nimar) | 36.9 | 22.3 | -2.9 | 19.1 | 12.2 | -1.4 |
| Burhanpur | 52.8 | 21.4 | -6.3 | 29.1 | 15.1 | -2.8 |
| Agar Malwa | 45.5 | 19.4 | -5.2 | 33.7 | 13.9 | -4.0 |
| Shajapur | 42.2 | 12.8 | -5.9 | 26.2 | 9.8 | -3.3 |
| Sidhi | 56.6 | 14.6 | -8.4 | 37.3 | 7.6 | -5.9 |
| Kachchh | 37.2 | 23.9 | -2.7 | 24.7 | 13.5 | -2.2 |
| Banas Kantha | 41.3 | 18.7 | -4.5 | 26.5 | 11.0 | -3.1 |
| Patan | 43.9 | 19.4 | -4.9 | 27.2 | 11.7 | -3.1 |
| Mahesana | 37.5 | 32.0 | -1.1 | 21.0 | 16.7 | -0.8 |
| Gandhinagar | 38.0 | 21.7 | -3.3 | 19.3 | 17.4 | -0.4 |
| Porbandar | 57.1 | 20.5 | -7.3 | 37.8 | 13.2 | -4.9 |
| Anand | 46.6 | 19.0 | -5.5 | 37.0 | 16.4 | -4.1 |
| Dohad | 39.7 | 20.2 | -3.9 | 30.7 | 16.3 | -2.9 |
| Narmada | 45.4 | 25.5 | -4.0 | 37.8 | 22.6 | -3.0 |
| Bharuch | 38.1 | 25.5 | -2.5 | 25.8 | 17.6 | -1.6 |
| The Dangs | 37.0 | 26.2 | -2.2 | 27.4 | 13.6 | -2.7 |
| Navsari | 45.8 | 27.3 | -3.7 | 25.5 | 13.9 | -2.3 |
| Valsad | 44.9 | 27.4 | -3.5 | 31.7 | 14.6 | -3.4 |
| Tapi | 58.0 | 28.8 | -5.8 | 37.7 | 17.2 | -4.1 |
| Ahmadabad | 45.9 | 29.5 | -3.3 | 31.8 | 16.1 | -3.1 |
| Aravali | 54.9 | 29.6 | -5.0 | 38.4 | 17.0 | -4.3 |
| Bhavnagar | 54.2 | 29.3 | -5.0 | 31.6 | 16.8 | -3.0 |
| Botad | 40.7 | 30.4 | -2.1 | 30.0 | 21.0 | -1.8 |
| Chhota Udaipur | 64.2 | 20.0 | -8.8 | 38.1 | 12.0 | -5.2 |
| Devbhoomi Dwarka | 41.2 | 23.9 | -3.4 | 30.8 | 15.5 | -3.1 |
| Gir Somnath | 45.9 | 23.9 | -4.4 | 28.0 | 19.2 | -1.7 |
| Jamnagar | 34.5 | 21.8 | -2.5 | 22.3 | 16.1 | -1.2 |
| Junagadh | 35.6 | 16.6 | -3.8 | 26.1 | 13.9 | -2.4 |
| Kheda | 42.4 | 17.2 | -5.0 | 30.4 | 12.5 | -3.6 |
| Mahisagar | 34.7 | 11.6 | -4.6 | 26.6 | 8.5 | -3.6 |
| Morbi | 39.2 | 20.4 | -3.8 | 20.3 | 13.5 | -1.4 |
| Panch Mahals | 47.0 | 18.2 | -5.8 | 31.6 | 13.3 | -3.7 |
| Rajkot | 51.2 | 21.0 | -6.0 | 33.4 | 15.8 | -3.5 |
| Sabar Kantha | 53.6 | 24.6 | -5.8 | 35.7 | 16.9 | -3.8 |
| Surendranagar | 44.6 | 15.1 | -5.9 | 26.5 | 6.3 | -4.0 |
| Vadodara | 40.4 | 21.6 | -3.7 | 14.4 | 10.0 | -0.9 |
| Surat | 57.3 | 47.7 | -1.9 | 60.9 | 47.9 | -2.6 |
| Amreli | 31.6 | 29.8 | -0.4 | 35.0 | 23.2 | -2.4 |
| Daman | 34.7 | 41.8 | 1.4 | 34.1 | 39.5 | 1.1 |
| Dadra & Nagar Haveli | 68.4 | 46.1 | -4.5 | 68.5 | 50.2 | -3.7 |
| Diu | 85.7 | 50.8 | -7.0 | 78.4 | 50.4 | -5.6 |
| Nandurbar | 76.7 | 61.3 | -3.1 | 78.2 | 65.3 | -2.6 |
| Dhule | 64.1 | 54.9 | -1.8 | 63.5 | 49.4 | -2.8 |
| Jalgaon | 77.4 | 43.6 | -6.8 | 70.6 | 40.3 | -6.1 |
| Buldana | 60.2 | 48.9 | -2.3 | 49.2 | 43.8 | -1.1 |
| Akola | 51.7 | 40.7 | -2.2 | 71.5 | 57.8 | -2.7 |
| Washim | 34.1 | 46.4 | 2.5 | 68.9 | 53.2 | -3.1 |
| Amravati | 6.2 | 5.0 | -0.2 | 59.2 | 50.6 | -1.7 |
| Wardha | 19.6 | 16.5 | -0.6 | 69.7 | 48.6 | -4.2 |
| Nagpur | 43.9 | 31.2 | -2.5 | 66.3 | 43.0 | -4.7 |
| Bhandara | 65.1 | 45.3 | -3.9 | 61.8 | 49.4 | -2.5 |
| Gondiya | 34.7 | 45.7 | 2.2 | 73.3 | 48.2 | -5.0 |
| Gadchiroli | 16.4 | 12.8 | -0.7 | 67.5 | 43.3 | -4.8 |
| Chandrapur | 57.8 | 41.7 | -3.2 | 78.2 | 56.5 | -4.3 |
| Yavatmal | 51.6 | 27.4 | -4.8 | 58.7 | 56.8 | -0.4 |
| Nanded | 5.3 | 1.7 | -0.7 | 53.1 | 46.0 | -1.4 |
| Hingoli | 73.7 | 51.5 | -4.4 | 72.5 | 51.6 | -4.2 |
| Parbhani | 49.2 | 25.2 | -4.8 | 55.6 | 31.0 | -4.9 |
| Jalna | 52.7 | 32.3 | -4.1 | 54.3 | 35.2 | -3.8 |
| Nashik | 15.8 | 11.8 | -0.8 | 62.9 | 53.3 | -1.9 |
| Mumbai Suburban | 54.5 | 38.7 | -3.2 | 55.9 | 51.6 | -0.9 |
| Mumbai | 46.6 | 27.2 | -3.9 | 55.0 | 33.5 | -4.3 |
| Raigarh | 57.6 | 31.5 | -5.2 | 57.8 | 36.2 | -4.3 |
| Pune | 75.4 | 44.3 | -6.2 | 64.7 | 50.5 | -2.9 |
| Ahmadnagar | 52.5 | 28.7 | -4.8 | 51.6 | 31.6 | -4.0 |
| Bid | 45.1 | 31.8 | -2.7 | 55.9 | 38.7 | -3.5 |
| Latur | 42.9 | 34.6 | -1.7 | 52.4 | 41.1 | -2.2 |
| Osmanabad | 55.7 | 23.2 | -6.5 | 60.6 | 32.3 | -5.7 |
| Solapur | 58.1 | 32.3 | -5.2 | 52.5 | 34.2 | -3.6 |
| Satara | 60.6 | 50.1 | -2.1 | 56.3 | 56.5 | 0.0 |
| Ratnagiri | 54.6 | 37.4 | -3.4 | 62.5 | 43.0 | -3.9 |
| Sindhudurg | 32.0 | 18.4 | -2.7 | 33.5 | 24.2 | -1.9 |
| Kolhapur | 33.8 | 18.3 | -3.1 | 37.9 | 20.9 | -3.4 |
| Sangli | 31.9 | 24.4 | -1.5 | 35.6 | 24.5 | -2.2 |
| Palghar | 27.6 | 20.2 | -1.5 | 31.2 | 25.2 | -1.2 |
| Thane | 21.8 | 14.5 | -1.4 | 32.3 | 19.9 | -2.5 |
| Aurangabad | 20.5 | 18.8 | -0.3 | 27.5 | 20.2 | -1.5 |
| Bagalkot | 35.6 | 14.3 | -4.3 | 35.2 | 18.9 | -3.3 |
| Bijapur | 14.8 | 22.3 | 1.5 | 21.6 | 23.8 | 0.4 |
| Bidar | 18.5 | 17.1 | -0.3 | 23.9 | 16.3 | -1.5 |
| Raichur | 25.2 | 19.0 | -1.3 | 29.5 | 23.1 | -1.3 |
| Koppal | 22.8 | 22.8 | 0.0 | 30.8 | 24.7 | -1.2 |
| Gadag | 11.5 | 2.8 | -1.7 | 45.5 | 30.6 | -3.0 |
| Dharwad | 9.5 | 3.6 | -1.2 | 52.0 | 42.8 | -1.8 |
| Uttara Kannada | 10.9 | 3.8 | -1.4 | 40.9 | 48.1 | 1.4 |
| Haveri | 6.0 | 1.4 | -0.9 | 44.8 | 47.8 | 0.6 |
| Bellary | 8.0 | 7.5 | -0.1 | 45.0 | 42.4 | -0.5 |
| Chitradurga | 5.4 | 2.0 | -0.7 | 66.9 | 57.0 | -2.0 |
| Davanagere | 9.6 | 4.3 | -1.0 | 60.1 | 56.6 | -0.7 |
| Shimoga | 6.9 | 1.4 | -1.1 | 63.2 | 47.9 | -3.1 |
| Chikmagalur | 5.9 | 2.3 | -0.7 | 59.1 | 53.0 | -1.2 |
| Tumkur | 8.2 | 4.6 | -0.7 | 54.9 | 41.1 | -2.8 |
| Bangalore | 11.9 | 3.4 | -1.7 | 58.5 | 39.5 | -3.8 |
| Mandya | 3.5 | 1.8 | -0.3 | 54.7 | 52.6 | -0.4 |
| Hassan | 10.0 | 6.2 | -0.8 | 53.9 | 37.9 | -3.2 |
| Dakshina Kannada | 5.2 | 1.3 | -0.8 | 63.6 | 51.3 | -2.5 |
| Kodagu | 8.3 | 5.0 | -0.6 | 45.8 | 35.5 | -2.1 |
| Mysore | 3.0 | 1.2 | -0.4 | 52.8 | 44.4 | -1.7 |
| Chamarajanagar | 10.8 | 4.0 | -1.4 | 49.9 | 24.7 | -5.1 |
| Gulbarga | 13.5 | 11.6 | -0.4 | 34.2 | 25.4 | -1.8 |
| Yadgir | 15.7 | 9.4 | -1.2 | 36.6 | 28.3 | -1.7 |
| Kolar | 29.7 | 8.0 | -4.3 | 64.2 | 33.7 | -6.1 |
| Chikkaballapura | 20.4 | 10.5 | -2.0 | 58.0 | 38.8 | -3.8 |
| Bangalore Rural | 18.3 | 18.5 | 0.0 | 27.5 | 21.8 | -1.2 |
| Ramanagara | 18.7 | 14.5 | -0.8 | 30.9 | 20.4 | -2.1 |
| Udupi | 56.2 | 34.9 | -4.2 | 79.7 | 51.1 | -5.7 |
| North Goa | 43.0 | 42.0 | -0.2 | 75.7 | 67.0 | -1.8 |
| South Goa | 45.4 | 39.6 | -1.1 | 71.9 | 51.5 | -4.1 |
| Lakshadweep | 59.4 | 27.2 | -6.4 | 78.7 | 46.4 | -6.5 |
| Wayanad | 40.9 | 29.6 | -2.3 | 76.5 | 60.7 | -3.2 |
| Kozhikode | 46.7 | 35.4 | -2.3 | 65.0 | 50.9 | -2.8 |
| Malappuram | 49.4 | 34.6 | -3.0 | 76.8 | 62.4 | -2.9 |
| Palakkad | 40.0 | 32.9 | -1.4 | 81.2 | 71.1 | -2.0 |
| Thrissur | 42.8 | 18.4 | -4.9 | 32.8 | 14.1 | -3.7 |
| Ernakulam | 46.2 | 22.2 | -4.8 | 22.9 | 10.9 | -2.4 |
| Idukki | 27.7 | 16.6 | -2.2 | 13.2 | 7.3 | -1.2 |
| Kottayam | 22.8 | 20.7 | -0.4 | 11.8 | 10.2 | -0.3 |
| Pathanamthitta | 55.7 | 25.1 | -6.1 | 41.8 | 22.9 | -3.8 |
| Kollam | 52.1 | 36.0 | -3.2 | 31.3 | 22.0 | -1.9 |
| Thiruvananthapuram | 43.6 | 23.3 | -4.1 | 27.4 | 10.2 | -3.4 |
| Kasaragod | 67.0 | 42.3 | -4.9 | 33.9 | 16.5 | -3.5 |
| Kannur |  | 54.4 |  |  | 17.8 |  |
| Alappuzha | 56.0 | 47.7 | -1.7 | 23.5 | 20.6 | -0.6 |
| Chennai | 50.6 | 32.6 | -3.6 | 29.8 | 16.2 | -2.7 |
| Kancheepuram | 54.6 | 38.2 | -3.3 | 21.7 | 17.7 | -0.8 |
| Vellore | 77.1 | 45.1 | -6.4 | 39.2 | 13.5 | -5.1 |
| Tiruvannamalai | 45.5 | 27.8 | -3.5 | 23.9 | 13.5 | -2.1 |
| Viluppuram | 37.4 | 32.5 | -1.0 | 33.9 | 30.5 | -0.7 |
| Salem | 60.2 | 51.1 | -1.8 | 29.7 | 19.2 | -2.1 |
| Namakkal | 38.0 | 39.0 | 0.2 | 20.6 | 16.1 | -0.9 |
| Erode | 44.8 | 38.4 | -1.3 | 27.3 | 16.2 | -2.2 |
| The Nilgiris | 60.5 | 50.3 | -2.0 | 30.4 | 18.2 | -2.4 |
| Dindigul | 51.6 | 33.0 | -3.7 | 25.4 | 12.6 | -2.6 |
| Karur | 67.5 | 48.9 | -3.7 | 30.3 | 20.1 | -2.0 |
| Tiruchirappalli | 55.2 | 49.9 | -1.1 | 27.6 | 18.0 | -1.9 |
| Perambalur | 42.0 | 38.1 | -0.8 | 34.6 | 18.7 | -3.2 |
| Ariyalur | 49.2 | 37.2 | -2.4 | 21.8 | 15.5 | -1.3 |
| Cuddalore | 76.9 | 40.4 | -7.3 | 32.5 | 14.4 | -3.6 |
| Nagapattinam | 56.5 | 46.0 | -2.1 | 30.8 | 13.5 | -3.5 |
| Thiruvarur | 52.3 | 45.2 | -1.4 | 21.6 | 13.5 | -1.6 |
| Thanjavur | 65.3 | 36.4 | -5.8 | 29.9 | 18.8 | -2.2 |
| Pudukkottai | 68.5 | 45.7 | -4.6 | 22.4 | 15.4 | -1.4 |
| Sivaganga | 51.3 | 39.2 | -2.4 | 27.3 | 13.8 | -2.7 |
| Madurai |  | 40.6 | 0.0 |  | 19.9 | 0.0 |
| Theni | 53.5 | 41.5 | -2.4 | 26.0 | 18.1 | -1.6 |
| Virudhunagar | 54.4 | 47.4 | -1.4 | 21.8 | 15.6 | -1.2 |
| Thoothukkudi | 60.7 | 38.8 | -4.4 | 28.6 | 10.3 | -3.7 |
| Tirunelveli | 72.8 | 53.2 | -3.9 | 24.2 | 15.8 | -1.7 |
| Kanniyakumari | 65.6 | 33.9 | -6.3 | 69.7 | 48.3 | -4.3 |
| Dharmapuri | 61.8 | 39.6 | -4.4 | 70.8 | 53.2 | -3.5 |
| Krishnagiri | 49.2 | 34.8 | -2.9 | 70.7 | 59.4 | -2.3 |
| Coimbatore | 70.5 | 30.9 | -7.9 | 74.6 | 47.8 | -5.4 |
| Tiruppur | 43.2 | 24.4 | -3.8 | 71.6 | 54.5 | -3.4 |
| Thiruvallur | 56.0 | 35.6 | -4.1 | 61.7 | 51.4 | -2.1 |
| Ramanathapuram | 24.0 | 9.3 | -2.9 | 53.9 | 43.2 | -2.1 |
| South Andaman | 28.5 | 11.9 | -3.3 | 55.3 | 34.6 | -4.2 |
| Nicobars | 23.6 | 11.5 | -2.4 | 44.1 | 19.6 | -4.9 |
| North & Middle Andaman | 21.8 | 13.3 | -1.7 | 42.3 | 23.9 | -3.7 |
| Adilabad | 12.8 | 12.5 | -0.1 | 46.8 | 29.1 | -3.5 |
| Bhadradri Kothagudem | 15.5 | 12.4 | -0.6 | 49.1 | 25.4 | -4.7 |
| Hyderabad | 22.3 | 15.1 | -1.4 | 63.0 | 44.6 | -3.7 |
| Jagtial | 9.9 | 15.7 | 1.2 | 47.0 | 33.9 | -2.6 |
| Jangaon | 14.4 | 9.2 | -1.0 | 47.3 | 24.1 | -4.7 |
| Jayashankar Bhupalapally | 22.6 | 8.1 | -2.9 | 47.2 | 37.3 | -2.0 |
| Jogulambha Gadwal | 41.1 | 13.7 | -5.5 | 69.7 | 35.8 | -6.8 |
| Kamareddy | 32.8 | 21.9 | -2.2 | 64.6 | 46.5 | -3.6 |
| Karimnagar | 53.3 | 22.5 | -6.2 | 33.6 | 12.0 | -4.3 |
| Khammam | 10.7 | 6.9 | -0.8 | 49.4 | 29.6 | -4.0 |
| Kumuram Bheem (Asifabad) | 6.6 | 1.2 | -1.1 | 54.7 | 54.0 | -0.2 |
| Mahabubabad | 46.3 | 39.7 | -1.3 | 59.2 | 47.5 | -2.4 |
| Mahbubnagar | 35.6 | 21.4 | -2.8 | 58.1 | 48.9 | -1.9 |
| Mancherial | 13.4 | 15.5 | 0.4 | 70.5 | 51.9 | -3.7 |
| Medak | 56.3 | 28.3 | -5.6 | 66.4 | 42.7 | -4.7 |
| Medchal-Malkajgiri | 27.9 | 11.7 | -3.2 | 28.2 | 12.8 | -3.1 |
| Nagarkurnool | 30.9 | 11.4 | -3.9 | 22.5 | 9.2 | -2.7 |
| Nalgonda | 6.3 | 0.6 | -1.1 | 25.2 | 23.5 | -0.3 |
| Nirmal | 44.2 | 9.5 | -6.9 | 22.8 | 7.4 | -3.1 |
| Nizamabad | 52.1 | 20.2 | -6.4 | 32.5 | 10.6 | -4.4 |
| Peddapalle | 42.6 | 25.2 | -3.5 | 81.4 | 73.7 | -1.5 |
| Rajanna Sircilla | 57.2 | 32.5 | -4.9 | 62.3 | 46.7 | -3.1 |
| Ranga Reddy | 38.5 | 27.2 | -2.3 | 27.2 | 15.7 | -2.3 |
| Sangareddy | 59.1 | 59.0 | 0.0 | 72.0 | 72.9 | 0.2 |
| Siddipet | 40.6 | 24.1 | -3.3 | 14.9 | 13.8 | -0.2 |
| Suryapet | 32.8 | 21.1 | -2.3 | 61.4 | 42.3 | -3.8 |
| Vikarabad | 42.8 | 21.0 | -4.4 | 56.4 | 45.5 | -2.2 |
| Wanaparthy | 42.0 | 49.4 | 1.5 | 66.1 | 66.3 | 0.1 |
| Warangal Rural | 7.9 | 5.6 | -0.4 | 28.7 | 27.0 | -0.3 |
| Warangal Urban |  |  |  |  |  |  |
| Yadadri Bhuvanagiri | 38.5 | 22.6 | -3.2 | 53.7 | 38.1 | -3.1 |
| Leh | 12.1 | 11.5 | -0.1 | 44.5 | 30.8 | -2.7 |
| Kargil | 17.1 | 20.0 | 0.6 | 28.0 | 29.2 | 0.3 |
| Aurangabad | 39.7 | 17.4 | -4.5 | 18.9 | 6.4 | -2.5 |

Note: AAC (% points per annum) = (P_t_ −P_t-n_ )∗(1/n ).

Table S2: Percentage Prevalence of Alcohol and Tobacco Consumption Among Women (15-49 years) Across Districts of India, NFHS 2016-21

| District Name | Alcohol | |  | Tobacco | |  |
| --- | --- | --- | --- | --- | --- | --- |
|  | 2016 | 2021 | AAC | 2016 | 2021 | AAC |
| Alluri Sitharama Raju | 5.8 | 1.8 | -0.8 | 6.5 | 5.7 | -0.2 |
| Anakapalli | 0.6 | 0.3 | 0.0 | 2.7 | 1.6 | -0.2 |
| Ananthapuramu | 0.1 | 0.3 | 0.0 | 1.3 | 1.8 | 0.1 |
| Annamayya | 0.1 | 0.1 | 0.0 | 2.6 | 1.0 | -0.3 |
| Bapatla | 0.0 | 0.1 | 0.0 | 0.8 | 0.3 | -0.1 |
| Chittoor | 0.1 | 0.2 | 0.0 | 2.3 | 1.9 | -0.1 |
| Dr. B.R. Ambedkar Konaseema | 0.1 | 0.3 | 0.0 | 0.9 | 0.4 | -0.1 |
| East Godavari | 0.1 | 0.2 | 0.0 | 1.2 | 0.4 | -0.2 |
| Eluru | 0.2 | 0.1 | 0.0 | 1.1 | 0.2 | -0.2 |
| Guntur | 0.0 | 0.0 | 0.0 | 0.6 | 0.4 | -0.1 |
| Kakinada | 0.1 | 0.2 | 0.0 | 1.1 | 0.5 | -0.1 |
| Krishna | 0.0 | 0.1 | 0.0 | 0.5 | 0.1 | -0.1 |
| Kurnool | 0.1 | 0.1 | 0.0 | 3.2 | 0.4 | -0.6 |
| Nandyal | 0.1 | 0.1 | 0.0 | 1.7 | 0.4 | -0.3 |
| Ntr | 0.0 | 0.1 | 0.0 | 0.6 | 0.1 | -0.1 |
| Palnadu | 0.1 | 0.0 | 0.0 | 0.9 | 0.3 | -0.1 |
| Parvathipuram Manyam | 0.4 | 0.2 | 0.0 | 6.3 | 1.7 | -0.9 |
| Prakasam | 0.1 | 0.1 | 0.0 | 1.2 | 0.3 | -0.2 |
| Sri Potti Sriramulu Nellore | 0.0 | 0.2 | 0.0 | 1.6 | 0.8 | -0.2 |
| Sri Sathya Sai | 0.2 | 0.3 | 0.0 | 1.7 | 4.1 | 0.5 |
| Srikakulam | 0.0 | 0.1 | 0.0 | 2.1 | 1.0 | -0.2 |
| Tirupati | 0.1 | 0.2 | 0.0 | 1.8 | 1.1 | -0.1 |
| Visakhapatnam | 0.4 | 0.3 | 0.0 | 1.9 | 1.0 | -0.2 |
| Vizianagaram | 0.2 | 0.3 | 0.0 | 4.5 | 0.9 | -0.7 |
| West Godavari | 0.1 | 0.1 | 0.0 | 1.2 | 0.3 | -0.2 |
| Y.S.R. | 0.0 | 0.1 | 0.0 | 3.6 | 0.7 | -0.6 |
| Muzaffarabad |  |  |  |  |  |  |
| Kolkata | 0.5 | 0.4 | 0.0 | 2.9 | 1.9 | -0.2 |
| Kupwara | 0.0 | 0.0 | 0.0 | 6.4 | 2.3 | -0.8 |
| Badgam | 0.0 | 0.1 | 0.0 | 6.4 | 0.6 | -1.2 |
| Belgaum | 1.6 | 5.1 | 0.7 | 0.3 | 3.5 | 0.6 |
| Punch | 0.0 | 0.2 | 0.0 | 1.0 | 0.4 | -0.1 |
| Kathua | 0.1 | 0.1 | 0.0 | 1.1 | 0.3 | -0.2 |
| Rajouri | 0.0 | 0.2 | 0.0 | 0.7 | 0.2 | -0.1 |
| Baramula | 0.0 | 0.1 | 0.0 | 2.7 | 3.6 | 0.2 |
| Bandipore | 0.0 | 0.1 | 0.0 | 10.9 | 4.9 | -1.2 |
| Srinagar | 0.0 | 0.0 | 0.0 | 0.5 | 0.5 | 0.0 |
| Ganderbal | 0.0 | 0.1 | 0.0 | 3.8 | 2.3 | -0.3 |
| Pulwama | 0.0 | 0.0 | 0.0 | 1.7 | 0.5 | -0.2 |
| Shupiyan | 0.0 | 0.1 | 0.0 | 3.0 | 0.2 | -0.6 |
| Anantnag | 0.0 | 0.1 | 0.0 | 3.9 | 0.2 | -0.7 |
| Kulgam | 0.0 | 0.0 | 0.0 | 4.2 | 0.2 | -0.8 |
| Doda | 0.0 | 0.1 | 0.0 | 1.2 | 0.6 | -0.1 |
| Ramban | 0.0 | 0.3 | 0.1 | 1.8 | 0.6 | -0.2 |
| Kishtwar | 0.1 | 0.1 | 0.0 | 1.8 | 1.1 | -0.1 |
| Udhampur | 0.0 | 0.3 | 0.1 | 1.2 | 1.0 | 0.0 |
| Reasi | 0.0 | 0.1 | 0.0 | 1.5 | 0.5 | -0.2 |
| Jammu | 0.1 | 0.1 | 0.0 | 0.7 | 0.2 | -0.1 |
| Samba | 0.0 | 0.4 | 0.1 | 0.7 | 0.2 | -0.1 |
| Chamba | 0.0 | 0.2 | 0.0 | 0.2 | 0.1 | 0.0 |
| Kangra | 0.0 | 0.5 | 0.1 | 0.2 | 0.1 | 0.0 |
| Lahul & Spiti | 0.3 | 0.7 | 0.1 | 0.2 | 0.1 | 0.0 |
| Kullu | 1.5 | 0.4 | -0.2 | 0.6 | 0.1 | -0.1 |
| Mandi | 0.0 | 0.1 | 0.0 | 0.3 | 0.1 | 0.0 |
| Hamirpur | 0.1 | 0.2 | 0.0 | 0.1 | 0.1 | 0.0 |
| Una | 0.0 | 0.1 | 0.0 | 0.2 | 0.2 | 0.0 |
| Bilaspur | 0.1 | 0.1 | 0.0 | 0.3 | 0.1 | 0.0 |
| Solan | 0.1 | 0.3 | 0.0 | 0.5 | 0.3 | 0.0 |
| Sirmaur | 0.0 | 0.1 | 0.0 | 0.6 | 0.1 | -0.1 |
| Shimla | 0.1 | 0.2 | 0.0 | 0.7 | 0.2 | -0.1 |
| Kinnaur | 0.2 | 0.3 | 0.0 | 0.2 | 0.1 | 0.0 |
| Yanam | 0.0 | 0.1 | 0.0 | 0.1 | 0.0 | 0.0 |
| Puducherry | 0.0 | 0.2 | 0.0 | 0.1 | 0.0 | 0.0 |
| Karaikal | 0.0 | 0.1 | 0.0 | 0.1 | 0.0 | 0.0 |
| Mahe | 0.0 | 0.1 | 0.0 | 0.1 | 0.0 | 0.0 |
| Kapurthala | 0.0 | 0.0 | 0.0 | 0.1 | 0.0 | 0.0 |
| Jalandhar | 0.0 | 0.2 | 0.0 | 0.1 | 0.1 | 0.0 |
| Hoshiarpur | 0.0 | 0.2 | 0.0 | 0.1 | 0.1 | 0.0 |
| Shahid Bhagat Singh Nagar | 0.0 | 0.1 | 0.0 | 0.1 | 0.1 | 0.0 |
| Fatehgarh Sahib | 0.0 | 0.1 | 0.0 | 0.1 | 0.0 | 0.0 |
| Ludhiana | 0.0 | 0.2 | 0.0 | 0.1 | 0.1 | 0.0 |
| Moga | 0.0 | 0.1 | 0.0 | 0.2 | 0.0 | 0.0 |
| Muktsar | 0.0 | 0.1 | 0.0 | 0.1 | 0.0 | 0.0 |
| Faridkot | 0.0 | 0.1 | 0.0 | 0.1 | 0.0 | 0.0 |
| Bathinda | 0.0 | 0.3 | 0.1 | 0.1 | 0.0 | 0.0 |
| Mansa | 0.0 | 0.1 | 0.0 | 0.1 | 0.0 | 0.0 |
| Patiala | 0.1 | 0.2 | 0.0 | 0.1 | 0.0 | 0.0 |
| Amritsar | 0.0 | 0.2 | 0.0 | 0.1 | 0.0 | 0.0 |
| Tarn Taran | 0.1 | 0.1 | 0.0 | 0.1 | 0.0 | 0.0 |
| Chandigarh | 0.2 | 0.2 | 0.0 | 0.3 | 0.0 | -0.1 |
| Uttarkashi | 0.0 | 0.2 | 0.0 | 0.6 | 0.1 | -0.1 |
| Chamoli | 0.0 | 0.1 | 0.0 | 1.2 | 0.1 | -0.2 |
| Rudraprayag | 0.0 | 0.0 | 0.0 | 0.5 | 0.1 | -0.1 |
| Tehri Garhwal | 0.0 | 0.1 | 0.0 | 0.5 | 0.1 | -0.1 |
| Dehradun | 0.1 | 0.1 | 0.0 | 1.6 | 0.3 | -0.3 |
| Garhwal | 0.0 | 0.1 | 0.0 | 1.3 | 0.5 | -0.2 |
| Pithoragarh | 0.2 | 0.1 | 0.0 | 2.2 | 0.2 | -0.4 |
| Bageshwar | 0.0 | 0.1 | 0.0 | 2.9 | 0.1 | -0.6 |
| Almora | 0.2 | 0.1 | 0.0 | 1.3 | 0.1 | -0.2 |
| Champawat | 0.0 | 0.1 | 0.0 | 2.7 | 0.4 | -0.5 |
| Nainital | 0.2 | 0.1 | 0.0 | 2.0 | 1.1 | -0.2 |
| Udham Singh Nagar | 0.0 | 0.1 | 0.0 | 5.0 | 0.3 | -0.9 |
| Hardwar | 0.0 | 0.2 | 0.0 | 3.3 | 1.5 | -0.4 |
| Panchkula | 0.1 | 0.2 | 0.0 | 0.3 | 0.1 | 0.0 |
| Ambala | 0.3 | 0.1 | 0.0 | 1.0 | 0.1 | -0.2 |
| Yamunanagar | 0.0 | 0.1 | 0.0 | 0.5 | 0.1 | -0.1 |
| Kurukshetra | 0.0 | 0.2 | 0.0 | 0.4 | 0.1 | -0.1 |
| Kaithal | 0.0 | 0.0 | 0.0 | 0.7 | 0.1 | -0.1 |
| Karnal | 0.0 | 0.1 | 0.0 | 0.6 | 0.2 | -0.1 |
| Panipat | 0.0 | 0.1 | 0.0 | 0.3 | 0.8 | 0.1 |
| Sonipat | 0.0 | 0.1 | 0.0 | 0.8 | 0.1 | -0.1 |
| Jind | 0.0 | 0.0 | 0.0 | 1.0 | 0.1 | -0.2 |
| Fatehabad | 0.0 | 0.2 | 0.0 | 0.5 | 0.1 | -0.1 |
| Sirsa | 0.0 | 0.1 | 0.0 | 0.3 | 0.1 | 0.0 |
| Hisar | 0.0 | 0.1 | 0.0 | 0.8 | 0.1 | -0.1 |
| Rohtak | 0.0 | 0.2 | 0.0 | 0.8 | 0.2 | -0.1 |
| Jhajjar | 0.0 | 0.1 | 0.0 | 1.7 | 0.1 | -0.3 |
| Mahendragarh | 0.0 | 0.1 | 0.0 | 1.3 | 0.3 | -0.2 |
| Rewari | 0.0 | 0.1 | 0.0 | 1.8 | 0.3 | -0.3 |
| Gurgaon | 0.1 | 0.2 | 0.0 | 2.2 | 0.3 | -0.4 |
| Mewat | 0.0 | 0.2 | 0.0 | 4.6 | 0.3 | -0.9 |
| Faridabad | 0.1 | 0.2 | 0.0 | 1.7 | 0.4 | -0.3 |
| Palwal | 0.1 | 0.1 | 0.0 | 3.9 | 0.9 | -0.6 |
| Ganganagar | 0.0 | 0.3 | 0.0 | 0.5 | 0.5 | 0.0 |
| Hanumangarh | 0.0 | 0.1 | 0.0 | 1.0 | 0.4 | -0.1 |
| Bikaner | 0.0 | 0.2 | 0.0 | 4.1 | 1.7 | -0.5 |
| Churu | 0.1 | 0.1 | 0.0 | 4.3 | 4.0 | 0.0 |
| Jhunjhunun | 0.0 | 0.4 | 0.1 | 3.7 | 1.8 | -0.4 |
| Alwar | 0.0 | 0.1 | 0.0 | 6.6 | 2.8 | -0.8 |
| Bharatpur | 0.0 | 0.1 | 0.0 | 7.0 | 6.0 | -0.2 |
| Dhaulpur | 0.0 | 0.1 | 0.0 | 7.8 | 6.0 | -0.4 |
| Karauli | 0.0 | 0.2 | 0.0 | 14.8 | 6.9 | -1.6 |
| Mirpur | 0.0 | 0.1 | 0.0 | 11.1 | 7.7 | -0.7 |
| Rupnagar | 0.0 | 0.1 | 0.0 | 11.1 | 3.6 | -1.5 |
| Sahibzada Ajit Singh Nagar | 0.0 | 0.2 | 0.0 | 5.9 | 1.3 | -0.9 |
| Sangrur | 0.0 | 0.1 | 0.0 | 7.6 | 3.0 | -0.9 |
| Barnala | 0.0 | 0.2 | 0.0 | 5.8 | 3.7 | -0.4 |
| Fazilka | 0.0 | 0.1 | 0.0 | 8.2 | 3.3 | -1.0 |
| Firozpur | 0.0 | 0.0 | 0.0 | 3.1 | 1.5 | -0.3 |
| Gurdaspur | 0.0 | 0.2 | 0.0 | 5.6 | 0.7 | -1.0 |
| Pathankot | 0.0 | 0.1 | 0.0 | 3.2 | 1.5 | -0.3 |
| Bhiwani | 0.0 | 0.1 | 0.0 | 4.6 | 3.2 | -0.3 |
| Charkhi Dadri | 0.0 | 0.1 | 0.0 | 6.5 | 0.9 | -1.1 |
| Central | 0.0 | 0.1 | 0.0 | 3.2 | 0.8 | -0.5 |
| East | 0.0 | 0.3 | 0.1 | 4.1 | 3.9 | 0.0 |
| New Delhi | 0.0 | 0.2 | 0.0 | 16.9 | 17.3 | 0.1 |
| North | 0.0 | 0.1 | 0.0 | 3.2 | 1.8 | -0.3 |
| North East | 0.0 | 0.1 | 0.0 | 5.0 | 0.9 | -0.8 |
| North West | 0.0 | 0.2 | 0.0 | 2.5 | 0.6 | -0.4 |
| Shahdara | 0.6 | 0.3 | -0.1 | 4.8 | 4.1 | -0.1 |
| South | 0.0 | 0.2 | 0.0 | 3.8 | 2.5 | -0.3 |
| South East | 0.0 | 0.2 | 0.0 | 9.9 | 8.3 | -0.3 |
| South West | 0.0 | 0.9 | 0.2 | 14.1 | 19.1 | 1.0 |
| West | 0.0 | 0.1 | 0.0 | 1.4 | 7.5 | 1.2 |
| Sawai Madhopur | 0.2 | 0.1 | 0.0 | 1.7 | 1.2 | -0.1 |
| Dausa | 0.0 | 0.1 | 0.0 | 1.0 | 1.1 | 0.0 |
| Jaipur | 0.0 | 0.2 | 0.0 | 4.1 | 0.6 | -0.7 |
| Sikar | 0.0 | 0.1 | 0.0 | 2.1 | 0.9 | -0.2 |
| Nagaur | 0.0 | 0.0 | 0.0 | 5.8 | 0.3 | -1.1 |
| Jodhpur | 0.0 | 0.2 | 0.0 | 1.7 | 0.4 | -0.3 |
| Jaisalmer | 0.0 | 0.1 | 0.0 | 2.4 | 0.5 | -0.4 |
| Barmer | 0.0 | 0.1 | 0.0 | 2.4 | 0.4 | -0.4 |
| Jalor | 0.0 | 0.1 | 0.0 | 3.8 | 0.6 | -0.6 |
| Sirohi | 0.0 | 0.1 | 0.0 | 2.2 | 0.3 | -0.4 |
| Pali | 0.0 | 0.1 | 0.0 | 7.6 | 0.9 | -1.3 |
| Ajmer | 0.0 | 0.2 | 0.0 | 8.5 | 4.3 | -0.8 |
| Tonk | 0.0 | 0.1 | 0.0 | 6.5 | 4.0 | -0.5 |
| Bundi | 0.0 | 0.2 | 0.0 | 7.3 | 5.2 | -0.4 |
| Bhilwara | 0.0 | 0.1 | 0.0 | 5.2 | 1.5 | -0.7 |
| Rajsamand | 0.1 | 0.3 | 0.0 | 2.2 | 0.8 | -0.3 |
| Dungarpur | 0.0 | 0.1 | 0.0 | 6.0 | 1.5 | -0.9 |
| Banswara | 0.0 | 0.1 | 0.0 | 7.8 | 1.5 | -1.3 |
| Chittaurgarh | 0.0 | 0.1 | 0.0 | 7.1 | 2.5 | -0.9 |
| Kota | 0.6 | 0.1 | -0.1 | 8.3 | 5.2 | -0.6 |
| Baran | 0.1 | 0.1 | 0.0 | 4.3 | 1.9 | -0.5 |
| Jhalawar | 0.1 | 0.2 | 0.0 | 7.0 | 4.3 | -0.5 |
| Udaipur | 0.0 | 0.1 | 0.0 | 5.6 | 3.3 | -0.5 |
| Pratapgarh | 0.0 | 0.1 | 0.0 | 4.6 | 2.7 | -0.4 |
| Saharanpur | 0.0 | 0.1 | 0.0 | 7.4 | 2.5 | -1.0 |
| Bijnor | 0.0 | 0.1 | 0.0 | 3.2 | 1.6 | -0.3 |
| Rampur | 0.0 | 0.1 | 0.0 | 3.6 | 3.6 | 0.0 |
| Jyotiba Phule Nagar | 0.0 | 0.2 | 0.0 | 7.3 | 4.8 | -0.5 |
| Meerut | 0.1 | 0.1 | 0.0 | 6.8 | 3.2 | -0.7 |
| Baghpat | 0.1 | 0.1 | 0.0 | 16.2 | 6.7 | -1.9 |
| Gautam Buddha Nagar | 0.0 | 0.1 | 0.0 | 5.0 | 2.2 | -0.6 |
| Bulandshahr | 0.0 | 0.4 | 0.1 | 3.2 | 1.5 | -0.3 |
| Aligarh | 0.0 | 0.1 | 0.0 | 17.4 | 4.8 | -2.5 |
| Mahamaya Nagar | 0.0 | 0.1 | 0.0 | 11.8 | 3.8 | -1.6 |
| Mathura | 0.0 | 0.2 | 0.0 | 16.8 | 7.3 | -1.9 |
| Agra | 0.1 | 0.2 | 0.0 | 13.4 | 4.1 | -1.8 |
| Firozabad | 0.0 | 0.4 | 0.1 | 15.3 | 7.2 | -1.6 |
| Mainpuri | 0.0 | 0.2 | 0.0 | 7.7 | 1.8 | -1.2 |
| Bareilly | 0.0 | 0.2 | 0.0 | 9.6 | 4.0 | -1.1 |
| Pilibhit | 0.0 | 0.1 | 0.0 | 9.1 | 4.8 | -0.9 |
| Shahjahanpur | 0.0 | 0.1 | 0.0 | 7.9 | 3.8 | -0.8 |
| Sitapur | 0.3 | 0.1 | 0.0 | 5.9 | 1.2 | -0.9 |
| Hardoi | 0.0 | 0.1 | 0.0 | 11.1 | 3.0 | -1.6 |
| Unnao | 0.1 | 0.1 | 0.0 | 21.2 | 16.0 | -1.1 |
| Lucknow | 0.0 | 0.1 | 0.0 | 19.4 | 12.8 | -1.3 |
| Farrukhabad | 10.5 | 10.7 | 0.0 | 17.9 | 11.0 | -1.4 |
| Kannauj | 0.0 | 0.2 | 0.0 | 17.9 | 7.0 | -2.2 |
| Etawah | 0.0 | 0.1 | 0.0 | 10.7 | 3.7 | -1.4 |
| Auraiya | 0.0 | 0.1 | 0.0 | 8.1 | 1.7 | -1.3 |
| Kanpur Dehat | 0.0 | 0.2 | 0.0 | 6.9 | 2.1 | -1.0 |
| Kanpur Nagar | 0.3 | 0.1 | 0.0 | 4.3 | 0.9 | -0.7 |
| Jalaun | 0.0 | 0.0 | 0.0 | 4.0 | 1.3 | -0.5 |
| Jhansi | 0.0 | 0.1 | 0.0 | 1.7 | 0.6 | -0.2 |
| Lalitpur | 0.0 | 0.1 | 0.0 | 2.0 | 2.8 | 0.2 |
| Hamirpur | 0.0 | 0.1 | 0.0 | 7.1 | 2.3 | -0.9 |
| Mahoba | 0.0 | 0.2 | 0.0 | 5.9 | 0.6 | -1.1 |
| Banda | 0.0 | 0.2 | 0.0 | 2.4 | 0.4 | -0.4 |
| Chitrakoot | 0.0 | 0.3 | 0.1 | 8.6 | 0.7 | -1.6 |
| Fatehpur | 0.0 | 0.1 | 0.0 | 3.5 | 0.7 | -0.6 |
| Pratapgarh | 0.2 | 0.2 | 0.0 | 6.2 | 1.3 | -1.0 |
| Kaushambi | 0.1 | 0.1 | 0.0 | 6.9 | 0.5 | -1.3 |
| Allahabad | 0.0 | 0.1 | 0.0 | 8.0 | 1.0 | -1.4 |
| Bara Banki | 0.0 | 0.1 | 0.0 | 8.5 | 1.0 | -1.5 |
| Faizabad | 3.6 | 0.2 | -0.7 | 8.5 | 0.9 | -1.5 |
| Ambedkar Nagar | 0.0 | 0.3 | 0.1 | 3.9 | 1.7 | -0.5 |
| Bahraich | 0.0 | 0.2 | 0.0 | 4.3 | 4.3 | 0.0 |
| Shrawasti | 0.1 | 0.2 | 0.0 | 1.2 | 0.9 | -0.1 |
| Gonda | 0.0 | 0.3 | 0.0 | 3.2 | 0.9 | -0.5 |
| Siddharthnagar | 0.1 | 0.2 | 0.0 | 3.5 | 0.5 | -0.6 |
| Basti | 0.0 | 0.1 | 0.0 | 3.3 | 0.2 | -0.6 |
| Sant Kabir Nagar | 0.0 | 0.1 | 0.0 | 2.9 | 0.3 | -0.5 |
| Maharajganj | 0.0 | 0.0 | 0.0 | 2.9 | 1.2 | -0.4 |
| Gorakhpur | 0.1 | 0.1 | 0.0 | 6.8 | 1.1 | -1.1 |
| Kushinagar | 0.6 | 0.3 | -0.1 | 5.8 | 2.1 | -0.7 |
| Deoria | 0.2 | 0.2 | 0.0 | 4.0 | 0.8 | -0.6 |
| Azamgarh | 0.1 | 0.1 | 0.0 | 2.2 | 0.5 | -0.3 |
| Mau | 0.3 | 0.3 | 0.0 | 4.3 | 1.1 | -0.7 |
| Ballia | 0.1 | 0.1 | 0.0 | 3.1 | 0.9 | -0.4 |
| Jaunpur | 0.0 | 0.2 | 0.0 | 1.8 | 0.5 | -0.3 |
| Ghazipur | 0.0 | 0.1 | 0.0 | 3.1 | 0.4 | -0.5 |
| Chandauli | 0.0 | 0.1 | 0.0 | 2.3 | 0.2 | -0.4 |
| Varanasi | 0.0 | 0.2 | 0.0 | 1.2 | 0.6 | -0.1 |
| Sant Ravidas Nagar | 0.1 | 0.3 | 0.0 | 2.3 | 0.5 | -0.3 |
| Mirzapur | 0.0 | 0.1 | 0.0 | 1.5 | 0.5 | -0.2 |
| Sonbhadra | 0.1 | 0.1 | 0.0 | 1.4 | 0.3 | -0.2 |
| Etah | 0.0 | 0.2 | 0.0 | 1.2 | 0.3 | -0.2 |
| Kanshiram Nagar | 0.0 | 0.1 | 0.0 | 1.7 | 0.3 | -0.3 |
| Balrampur | 0.3 | 0.1 | 0.0 | 2.3 | 0.4 | -0.4 |
| Amethi | 0.5 | 0.3 | 0.0 | 3.0 | 0.2 | -0.6 |
| Budaun | 0.0 | 0.1 | 0.0 | 0.6 | 0.3 | 0.0 |
| Ghaziabad | 0.7 | 0.4 | -0.1 | 0.7 | 0.3 | -0.1 |
| Hapur | 0.1 | 0.1 | 0.0 | 0.9 | 0.2 | -0.1 |
| Moradabad | 0.3 | 0.1 | 0.0 | 1.3 | 0.3 | -0.2 |
| Muzaffarnagar | 0.0 | 0.1 | 0.0 | 0.7 | 0.2 | -0.1 |
| Rae Bareli | 0.2 | 0.2 | 0.0 | 4.3 | 0.4 | -0.8 |
| Sambhal | 0.2 | 0.1 | 0.0 | 0.4 | 0.2 | 0.0 |
| Shamli | 0.1 | 0.2 | 0.0 | 1.4 | 0.5 | -0.2 |
| Sultanpur | 0.0 | 0.1 | 0.0 | 0.7 | 0.1 | -0.1 |
| Kheri | 0.2 | 0.2 | 0.0 | 1.6 | 0.3 | -0.3 |
| Pashchim Champaran | 0.2 | 0.5 | 0.0 | 1.0 | 0.1 | -0.2 |
| Purba Champaran | 0.0 | 0.3 | 0.1 | 0.5 | 0.2 | -0.1 |
| Sheohar | 0.0 | 0.3 | 0.1 | 2.3 | 1.3 | -0.2 |
| Sitamarhi | 0.2 | 0.2 | 0.0 | 2.1 | 0.4 | -0.3 |
| Madhubani | 35.0 | 18.5 | -3.3 | 5.2 | 5.2 | 0.0 |
| Supaul | 22.5 | 16.3 | -1.2 | 4.6 | 3.4 | -0.2 |
| Araria | 24.4 | 10.8 | -2.7 | 5.1 | 5.2 | 0.0 |
| Kishanganj | 19.4 | 13.4 | -1.2 | 8.4 | 10.8 | 0.5 |
| Purnia | 23.2 | 18.9 | -0.9 | 4.9 | 4.7 | 0.0 |
| Katihar | 19.7 | 15.0 | -0.9 | 15.1 | 10.8 | -0.9 |
| Madhepura | 22.0 | 21.1 | -0.2 | 16.5 | 12.4 | -0.8 |
| Saharsa | 17.7 | 14.8 | -0.6 | 15.6 | 13.1 | -0.5 |
| Darbhanga | 38.8 | 35.4 | -0.7 | 18.0 | 11.3 | -1.3 |
| Muzaffarpur | 23.6 | 16.3 | -1.5 | 12.0 | 9.4 | -0.5 |
| Gopalganj | 32.0 | 25.1 | -1.4 | 26.6 | 8.9 | -3.5 |
| Siwan | 8.9 | 13.4 | 0.9 | 5.8 | 8.9 | 0.6 |
| Saran | 32.3 | 19.7 | -2.5 | 14.3 | 7.2 | -1.4 |
| Vaishali | 12.0 | 15.4 | 0.7 | 10.9 | 6.8 | -0.8 |
| Samastipur | 42.4 | 23.1 | -3.9 | 16.2 | 8.0 | -1.6 |
| Begusarai | 0.6 | 0.2 | -0.1 | 9.4 | 6.0 | -0.7 |
| Khagaria | 1.3 | 0.3 | -0.2 | 50.4 | 21.1 | -5.9 |
| Bhagalpur | 0.6 | 0.2 | -0.1 | 14.2 | 8.5 | -1.1 |
| Banka | 1.3 | 0.8 | -0.1 | 25.1 | 14.1 | -2.2 |
| Munger | 3.7 | 1.3 | -0.5 | 34.5 | 14.6 | -4.0 |
| Lakhisarai | 3.7 | 0.6 | -0.6 | 21.5 | 9.7 | -2.4 |
| Sheikhpura | 0.7 | 0.4 | -0.1 | 23.0 | 14.9 | -1.6 |
| Nalanda | 0.4 | 0.3 | 0.0 | 16.2 | 8.7 | -1.5 |
| Patna | 1.6 | 0.4 | -0.2 | 16.8 | 9.3 | -1.5 |
| Bhojpur | 3.6 | 1.1 | -0.5 | 26.6 | 13.9 | -2.6 |
| Buxer | 4.7 | 0.7 | -0.8 | 36.1 | 15.1 | -4.2 |
| Kaimur (Bhabua) | 7.2 | 1.2 | -1.2 | 58.1 | 47.8 | -2.1 |
| Rohtas | 6.7 | 1.4 | -1.1 | 21.9 | 14.8 | -1.4 |
| Gaya | 13.4 | 0.4 | -2.6 | 69.6 | 61.6 | -1.6 |
| Nawada | 3.6 | 0.5 | -0.6 | 52.1 | 45.6 | -1.3 |
| Jamui | 2.2 | 0.4 | -0.3 | 47.9 | 43.8 | -0.8 |
| Jehanabad | 4.8 | 0.3 | -0.9 | 44.1 | 36.4 | -1.5 |
| Arwal | 3.7 | 0.4 | -0.6 | 46.4 | 40.7 | -1.1 |
| North District | 9.3 | 0.6 | -1.7 | 45.1 | 32.0 | -2.6 |
| West District | 7.0 | 0.9 | -1.2 | 52.4 | 49.3 | -0.6 |
| South District | 2.8 | 0.3 | -0.5 | 62.2 | 64.2 | 0.4 |
| East District | 4.7 | 0.7 | -0.8 | 78.6 | 59.9 | -3.8 |
| Tawang | 5.7 | 0.5 | -1.0 | 54.2 | 36.6 | -3.5 |
| West Kameng | 2.6 | 2.6 | 0.0 | 58.8 | 62.7 | 0.8 |
| East Kameng | 3.0 | 0.3 | -0.5 | 75.1 | 57.3 | -3.5 |
| Papum Pare | 3.0 | 0.7 | -0.5 | 69.2 | 53.4 | -3.2 |
| Upper Subansiri | 2.0 | 0.6 | -0.3 | 60.1 | 53.1 | -1.4 |
| Upper Siang | 7.9 | 5.8 | -0.4 | 42.5 | 46.5 | 0.8 |
| Changlang | 1.2 | 1.0 | 0.0 | 17.2 | 12.9 | -0.9 |
| Lower Subansiri | 0.8 | 0.4 | -0.1 | 44.8 | 22.1 | -4.5 |
| Dibang Valley | 0.6 | 0.7 | 0.0 | 39.2 | 21.2 | -3.6 |
| Lower Dibang Valley | 10.3 | 11.1 | 0.2 | 11.3 | 13.9 | 0.5 |
| Anjaw | 3.5 | 3.4 | 0.0 | 6.9 | 8.2 | 0.3 |
| East Siang | 0.1 | 0.4 | 0.1 | 16.1 | 6.2 | -2.0 |
| Kra Daadi | 3.8 | 1.3 | -0.5 | 16.4 | 20.1 | 0.7 |
| Kurung Kumey | 22.8 | 13.5 | -1.8 | 19.0 | 12.0 | -1.4 |
| Lohit | 34.8 | 26.4 | -1.7 | 19.5 | 8.4 | -2.2 |
| Langding | 11.2 | 12.2 | 0.2 | 30.8 | 17.3 | -2.7 |
| Namsai | 8.4 | 14.7 | 1.3 | 14.8 | 18.1 | 0.7 |
| Siang | 2.5 | 8.2 | 1.1 | 12.5 | 14.5 | 0.4 |
| Tirap | 26.2 | 20.6 | -1.1 | 23.1 | 11.2 | -2.4 |
| West Siang | 0.4 | 1.9 | 0.3 | 50.9 | 19.6 | -6.3 |
| Mon | 0.3 | 1.0 | 0.1 | 42.2 | 27.4 | -3.0 |
| Mokokchung | 0.4 | 2.7 | 0.5 | 36.6 | 32.6 | -0.8 |
| Zunheboto | 0.3 | 0.7 | 0.1 | 16.8 | 11.8 | -1.0 |
| Wokha | 10.4 | 6.6 | -0.8 | 16.6 | 10.5 | -1.2 |
| Dimapur | 0.4 | 1.8 | 0.3 | 6.8 | 8.0 | 0.2 |
| Phek | 1.8 | 2.5 | 0.1 | 7.2 | 7.8 | 0.1 |
| Tuensang | 0.7 | 1.2 | 0.1 | 14.9 | 6.6 | -1.7 |
| Longleng | 13.6 | 8.8 | -1.0 | 4.0 | 5.8 | 0.4 |
| Kiphire | 0.6 | 0.2 | -0.1 | 15.4 | 2.9 | -2.5 |
| Kohima | 10.9 | 8.1 | -0.6 | 11.1 | 4.9 | -1.2 |
| Peren | 10.0 | 3.8 | -1.2 | 11.2 | 7.4 | -0.8 |
| Senapati | 2.5 | 1.7 | -0.2 | 16.8 | 12.2 | -0.9 |
| Tamenglong | 0.0 | 0.2 | 0.0 | 21.0 | 13.8 | -1.4 |
| Churachandpur | 0.2 | 0.8 | 0.1 | 6.5 | 3.1 | -0.7 |
| Bishnupur | 2.0 | 1.3 | -0.2 | 6.0 | 4.5 | -0.3 |
| Thoubal | 0.7 | 0.2 | -0.1 | 3.6 | 3.1 | -0.1 |
| Imphal West | 0.1 | 0.3 | 0.0 | 2.2 | 0.7 | -0.3 |
| Imphal East | 0.7 | 0.7 | 0.0 | 5.3 | 4.2 | -0.2 |
| Ukhrul | 0.0 | 0.1 | 0.0 | 4.1 | 2.4 | -0.3 |
| Chandel | 0.3 | 0.3 | 0.0 | 5.3 | 1.7 | -0.7 |
| Mamit | 0.1 | 0.9 | 0.2 | 3.6 | 1.5 | -0.4 |
| Kolasib | 0.2 | 0.2 | 0.0 | 19.9 | 13.3 | -1.3 |
| Aizawl | 0.3 | 0.7 | 0.1 | 28.7 | 23.8 | -1.0 |
| Champhai | 0.1 | 0.2 | 0.0 | 4.6 | 2.1 | -0.5 |
| Serchhip | 0.8 | 0.6 | 0.0 | 13.1 | 10.4 | -0.5 |
| Lunglei | 0.1 | 1.2 | 0.2 | 7.9 | 3.8 | -0.8 |
| Lawngtlai | 2.0 | 0.9 | -0.2 | 2.6 | 0.8 | -0.4 |
| Saiha | 3.0 | 2.4 | -0.1 | 2.9 | 1.4 | -0.3 |
| Dhalai | 0.1 | 0.2 | 0.0 | 0.4 | 0.2 | 0.0 |
| Gomati | 0.1 | 0.5 | 0.1 | 1.4 | 0.5 | -0.2 |
| Khowai | 0.3 | 1.0 | 0.1 | 2.9 | 1.4 | -0.3 |
| North Tripura | 1.4 | 1.6 | 0.0 | 0.9 | 0.4 | -0.1 |
| South Tripura | 3.8 | 3.3 | -0.1 | 4.9 | 0.4 | -0.9 |
| Unakoti | 2.1 | 2.7 | 0.1 | 3.2 | 0.4 | -0.6 |
| West Tripura | 0.4 | 0.4 | 0.0 | 7.3 | 3.6 | -0.7 |
| Sepahijala | 0.5 | 0.6 | 0.0 | 7.1 | 3.8 | -0.7 |
| South Garo Hills | 2.5 | 2.3 | 0.0 | 3.4 | 1.7 | -0.4 |
| Ribhoi | 1.3 | 1.4 | 0.0 | 6.5 | 0.8 | -1.2 |
| East Khasi Hills | 1.6 | 0.8 | -0.2 | 4.4 | 1.1 | -0.7 |
| East Garo Hills | 4.6 | 5.4 | 0.2 | 3.5 | 1.8 | -0.3 |
| East Jaintia Hills | 0.4 | 1.4 | 0.2 | 1.6 | 1.1 | -0.1 |
| North Garo Hills | 1.0 | 2.4 | 0.3 | 4.5 | 2.5 | -0.4 |
| South West Garo Hills | 3.9 | 3.1 | -0.1 | 4.3 | 1.6 | -0.5 |
| South West Khasi Hills | 1.6 | 3.2 | 0.3 | 9.6 | 7.4 | -0.4 |
| West Garo Hills | 2.5 | 2.1 | -0.1 | 5.0 | 0.9 | -0.8 |
| West Jaintia Hills | 12.7 | 13.7 | 0.2 | 5.1 | 3.4 | -0.3 |
| West Khasi Hills | 9.7 | 7.3 | -0.5 | 9.4 | 1.3 | -1.6 |
| Kokrajhar | 11.8 | 10.2 | -0.3 | 11.8 | 3.5 | -1.7 |
| Goalpara | 37.0 | 22.5 | -2.9 | 13.6 | 5.7 | -1.6 |
| Barpeta | 5.5 | 6.4 | 0.2 | 11.1 | 2.5 | -1.7 |
| Morigaon | 0.0 | 1.1 | 0.2 | 15.3 | 7.2 | -1.6 |
| Lakhimpur | 0.6 | 1.0 | 0.1 | 11.7 | 8.0 | -0.7 |
| Dhemaji | 0.7 | 4.6 | 0.8 | 12.9 | 7.7 | -1.0 |
| Tinsukia | 1.8 | 3.8 | 0.4 | 13.2 | 9.6 | -0.7 |
| Dibrugarh | 3.1 | 6.4 | 0.7 | 14.5 | 7.6 | -1.4 |
| Golaghat | 9.2 | 11.2 | 0.4 | 24.3 | 19.7 | -0.9 |
| Dima Hasao | 8.9 | 12.0 | 0.6 | 23.9 | 20.5 | -0.7 |
| Cachar | 0.9 | 2.5 | 0.3 | 15.4 | 12.7 | -0.5 |
| Karimganj | 0.1 | 0.3 | 0.0 | 14.5 | 16.9 | 0.5 |
| Hailakandi | 0.1 | 0.2 | 0.0 | 19.2 | 15.6 | -0.7 |
| Bongaigaon | 0.4 | 0.3 | 0.0 | 11.4 | 8.8 | -0.5 |
| Chirang | 2.0 | 2.1 | 0.0 | 21.7 | 17.5 | -0.8 |
| Kamrup | 1.0 | 1.0 | 0.0 | 16.9 | 11.2 | -1.2 |
| Kamrup Metropolitan | 2.0 | 2.3 | 0.1 | 12.2 | 6.1 | -1.2 |
| Nalbari | 0.2 | 0.2 | 0.0 | 6.5 | 2.7 | -0.8 |
| Baksa | 0.3 | 0.2 | 0.0 | 8.3 | 7.0 | -0.3 |
| Darrang | 0.1 | 0.1 | 0.0 | 15.5 | 9.5 | -1.2 |
| Udalguri | 0.1 | 0.2 | 0.0 | 10.3 | 6.4 | -0.8 |
| Biswanath | 2.9 | 2.8 | 0.0 | 5.9 | 5.7 | 0.0 |
| Charaideo | 1.1 | 0.3 | -0.2 | 14.8 | 8.4 | -1.3 |
| Dhubri | 0.3 | 0.5 | 0.0 | 17.1 | 6.6 | -2.1 |
| Hojai | 0.2 | 0.4 | 0.0 | 11.3 | 4.2 | -1.4 |
| Jorhat | 0.1 | 0.3 | 0.0 | 17.8 | 8.1 | -1.9 |
| Karbi Anglong | 0.1 | 0.5 | 0.1 | 19.0 | 12.0 | -1.4 |
| Majuli | 0.9 | 0.6 | -0.1 | 15.9 | 8.7 | -1.4 |
| Nagaon | 5.1 | 2.0 | -0.6 | 25.3 | 15.9 | -1.9 |
| Sivasagar | 3.0 | 3.4 | 0.1 | 27.8 | 22.2 | -1.1 |
| Sonitpur | 3.4 | 6.8 | 0.7 | 25.3 | 23.8 | -0.3 |
| South Salmara-Mankachar | 7.5 | 13.7 | 1.2 | 28.6 | 18.5 | -2.0 |
| Karbi Anglong West | 2.9 | 2.3 | -0.1 | 17.0 | 8.6 | -1.7 |
| Darjiling | 9.1 | 6.0 | -0.6 | 19.9 | 6.1 | -2.8 |
| Jalpaiguri | 4.1 | 1.5 | -0.5 | 21.8 | 5.4 | -3.3 |
| Koch Bihar | 2.7 | 1.3 | -0.3 | 17.7 | 4.6 | -2.6 |
| Uttar Dinajpur | 1.4 | 0.6 | -0.2 | 10.6 | 3.9 | -1.3 |
| Dakshin Dinajpur | 2.5 | 1.9 | -0.1 | 22.9 | 2.8 | -4.0 |
| Maldah | 1.5 | 0.7 | -0.2 | 16.1 | 9.1 | -1.4 |
| Murshidabad | 2.1 | 1.7 | -0.1 | 25.2 | 8.4 | -3.4 |
| Birbhum | 2.7 | 1.3 | -0.3 | 25.5 | 6.7 | -3.8 |
| Nadia | 3.2 | 1.6 | -0.3 | 27.7 | 5.4 | -4.5 |
| North Twenty Four Parganas | 21.2 | 21.4 | 0.0 | 23.3 | 25.9 | 0.5 |
| Hugli | 12.3 | 24.3 | 2.4 | 21.3 | 32.3 | 2.2 |
| Bankura | 0.2 | 0.2 | 0.0 | 18.9 | 8.5 | -2.1 |
| Puruliya | 0.0 | 0.1 | 0.0 | 4.5 | 1.9 | -0.5 |
| South Twenty Four Parganas | 0.1 | 0.2 | 0.0 | 4.5 | 1.5 | -0.6 |
| Paschim Medinipur | 0.1 | 0.1 | 0.0 | 4.5 | 2.3 | -0.4 |
| Purba Medinipur | 0.0 | 0.1 | 0.0 | 7.1 | 2.8 | -0.9 |
| Paschim Barddhaman | 0.1 | 0.2 | 0.0 | 6.8 | 4.5 | -0.5 |
| Purba Barddhaman | 0.2 | 0.2 | 0.0 | 7.1 | 1.5 | -1.1 |
| Haora | 0.1 | 0.4 | 0.0 | 11.0 | 7.5 | -0.7 |
| Garhwa | 0.0 | 0.1 | 0.0 | 11.9 | 7.9 | -0.8 |
| Chatra | 0.1 | 0.2 | 0.0 | 10.1 | 7.8 | -0.5 |
| Kodarma | 0.0 | 0.1 | 0.0 | 13.6 | 10.4 | -0.6 |
| Giridih | 0.0 | 0.1 | 0.0 | 5.7 | 3.1 | -0.5 |
| Deoghar | 0.0 | 0.2 | 0.0 | 2.6 | 5.1 | 0.5 |
| Godda | 0.9 | 1.0 | 0.0 | 11.6 | 7.9 | -0.7 |
| Sahibganj | 0.1 | 0.2 | 0.0 | 2.6 | 1.1 | -0.3 |
| Pakur | 0.2 | 0.2 | 0.0 | 1.9 | 0.6 | -0.3 |
| Dhanbad | 0.2 | 0.1 | 0.0 | 4.4 | 0.8 | -0.7 |
| Bokaro | 0.1 | 0.3 | 0.1 | 3.9 | 1.1 | -0.6 |
| Lohardaga | 0.5 | 0.3 | 0.0 | 5.6 | 2.2 | -0.7 |
| Purbi Singhbhum | 6.3 | 3.3 | -0.6 | 7.6 | 4.2 | -0.7 |
| Palamu | 0.1 | 0.1 | 0.0 | 3.3 | 1.2 | -0.4 |
| Latehar | 1.1 | 0.5 | -0.1 | 9.5 | 2.7 | -1.4 |
| Hazaribagh | 2.1 | 1.0 | -0.2 | 19.9 | 2.5 | -3.5 |
| Ramgarh | 0.0 | 0.2 | 0.0 | 3.1 | 3.5 | 0.1 |
| Dumka | 0.2 | 0.0 | 0.0 | 7.8 | 3.5 | -0.9 |
| Jamtara | 0.1 | 0.1 | 0.0 | 5.2 | 4.1 | -0.2 |
| Ranchi | 2.1 | 0.1 | -0.4 | 7.4 | 4.2 | -0.7 |
| Khunti | 0.3 | 0.1 | 0.0 | 15.9 | 4.3 | -2.3 |
| Gumla | 1.5 | 0.5 | -0.2 | 12.4 | 7.1 | -1.1 |
| Simdega | 1.7 | 0.1 | -0.3 | 8.4 | 5.7 | -0.5 |
| Pashchimi Singhbhum | 0.3 | 0.4 | 0.0 | 10.5 | 10.9 | 0.1 |
| Saraikela-Kharsawan | 0.1 | 0.2 | 0.0 | 14.7 | 7.7 | -1.4 |
| Bargarh | 0.1 | 0.2 | 0.0 | 17.9 | 13.9 | -0.8 |
| Jharsuguda | 0.2 | 0.2 | 0.0 | 19.7 | 23.4 | 0.7 |
| Sambalpur | 6.1 | 4.2 | -0.4 | 20.1 | 14.8 | -1.1 |
| Debagarh | 6.1 | 1.4 | -1.0 | 37.2 | 16.0 | -4.2 |
| Sundargarh | 1.1 | 0.7 | -0.1 | 22.4 | 15.6 | -1.4 |
| Kendujhar | 0.8 | 0.6 | 0.0 | 27.6 | 22.6 | -1.0 |
| Mayurbhanj | 1.2 | 0.8 | -0.1 | 20.2 | 7.2 | -2.6 |
| Baleshwar | 0.1 | 0.2 | 0.0 | 8.2 | 3.0 | -1.0 |
| Bhadrak | 0.6 | 0.1 | -0.1 | 6.8 | 3.3 | -0.7 |
| Kendrapara | 1.3 | 0.9 | -0.1 | 7.6 | 5.2 | -0.5 |
| Cuttack | 2.7 | 2.0 | -0.2 | 11.7 | 7.4 | -0.8 |
| Jajapur | 1.2 | 0.5 | -0.1 | 6.0 | 0.8 | -1.0 |
| Dhenkanal | 5.2 | 0.4 | -1.0 | 7.5 | 2.1 | -1.1 |
| Anugul | 51.5 | 4.0 | -9.5 | 18.2 | 5.3 | -2.6 |
| Nayagarh | 0.3 | 0.3 | 0.0 | 10.0 | 2.3 | -1.5 |
| Khordha | 0.3 | 0.3 | 0.0 | 9.5 | 2.5 | -1.4 |
| Puri | 0.2 | 0.2 | 0.0 | 8.7 | 4.9 | -0.7 |
| Ganjam | 0.0 | 0.1 | 0.0 | 7.0 | 4.1 | -0.6 |
| Gajapati | 0.0 | 0.1 | 0.0 | 5.3 | 3.4 | -0.4 |
| Kandhamal | 0.0 | 0.2 | 0.0 | 4.8 | 2.4 | -0.5 |
| Baudh | 0.0 | 0.0 | 0.0 | 10.7 | 3.9 | -1.3 |
| Subarnapur | 0.0 | 0.1 | 0.0 | 7.4 | 13.2 | 1.2 |
| Balangir | 0.0 | 0.2 | 0.0 | 10.0 | 5.0 | -1.0 |
| Nuapada | 0.1 | 0.4 | 0.1 | 9.9 | 8.9 | -0.2 |
| Kalahandi | 0.1 | 0.4 | 0.1 | 8.9 | 9.4 | 0.1 |
| Rayagada | 0.1 | 0.2 | 0.0 | 10.8 | 5.7 | -1.0 |
| Nabarangapur | 1.5 | 0.5 | -0.2 | 12.3 | 6.4 | -1.2 |
| Koraput | 0.0 | 0.4 | 0.1 | 3.3 | 2.3 | -0.2 |
| Malkangiri | 0.2 | 0.3 | 0.0 | 3.0 | 1.1 | -0.4 |
| Jagatsinghapur | 0.6 | 0.4 | -0.1 | 5.3 | 4.2 | -0.2 |
| Balrampur | 1.2 | 0.1 | -0.2 | 0.4 | 1.3 | 0.2 |
| Koriya | 0.0 | 0.1 | 0.0 | 1.8 | 0.8 | -0.2 |
| Jashpur | 0.9 | 0.7 | 0.0 | 12.4 | 16.9 | 0.9 |
| Raigarh | 0.0 | 0.1 | 0.0 | 8.2 | 8.6 | 0.1 |
| Korba | 0.0 | 0.4 | 0.1 | 4.9 | 5.9 | 0.2 |
| Janjgir-Champa | 0.1 | 0.1 | 0.0 | 4.2 | 1.1 | -0.6 |
| Kabeerdham | 0.0 | 0.1 | 0.0 | 2.9 | 1.6 | -0.3 |
| Rajnandgaon | 0.0 | 0.1 | 0.0 | 4.1 | 5.9 | 0.3 |
| Mahasamund | 0.1 | 0.6 | 0.1 | 3.7 | 1.3 | -0.5 |
| Dhamtari | 0.6 | 0.3 | -0.1 | 5.5 | 1.8 | -0.8 |
| Uttar Bastar Kanker | 0.1 | 0.2 | 0.0 | 4.0 | 0.9 | -0.6 |
| Narayanpur | 0.0 | 0.3 | 0.1 | 7.1 | 6.8 | -0.1 |
| Bijapur | 0.0 | 0.2 | 0.0 | 7.0 | 3.1 | -0.8 |
| Balod | 0.3 | 1.2 | 0.2 | 7.4 | 8.7 | 0.3 |
| Baloda Bazar | 0.0 | 0.5 | 0.1 | 3.4 | 1.6 | -0.4 |
| Bastar | 0.0 | 0.4 | 0.1 | 8.1 | 5.9 | -0.4 |
| Bemetara | 0.0 | 0.2 | 0.0 | 3.3 | 1.0 | -0.5 |
| Bilaspur | 0.0 | 0.1 | 0.0 | 3.5 | 0.9 | -0.5 |
| Dantewada | 0.0 | 0.1 | 0.0 | 1.8 | 1.2 | -0.1 |
| Durg | 0.0 | 0.1 | 0.0 | 1.9 | 1.5 | -0.1 |
| Gariaband | 0.0 | 0.1 | 0.0 | 6.6 | 8.0 | 0.3 |
| Kodagaon | 0.1 | 0.2 | 0.0 | 3.4 | 0.7 | -0.5 |
| Mungeli | 0.7 | 0.2 | -0.1 | 3.1 | 1.0 | -0.4 |
| Raipur | 0.0 | 0.1 | 0.0 | 3.2 | 4.1 | 0.2 |
| Sukma | 0.0 | 0.1 | 0.0 | 7.1 | 5.0 | -0.4 |
| Surguja | 0.0 | 0.1 | 0.0 | 3.8 | 5.0 | 0.2 |
| Surajpur | 0.4 | 0.3 | 0.0 | 2.7 | 0.7 | -0.4 |
| Sheopur | 0.0 | 0.1 | 0.0 | 1.8 | 0.2 | -0.3 |
| Morena | 0.0 | 0.0 | 0.0 | 1.1 | 0.7 | -0.1 |
| Bhind | 0.0 | 0.1 | 0.0 | 4.4 | 3.1 | -0.2 |
| Gwalior | 0.1 | 0.1 | 0.0 | 14.0 | 15.4 | 0.3 |
| Datia | 0.0 | 0.4 | 0.1 | 4.4 | 5.4 | 0.2 |
| Shivpuri | 0.0 | 0.1 | 0.0 | 3.9 | 2.5 | -0.3 |
| Tikamgarh | 0.1 | 0.0 | 0.0 | 2.9 | 2.0 | -0.2 |
| Chhatarpur | 0.0 | 0.1 | 0.0 | 1.4 | 3.4 | 0.4 |
| Panna | 0.0 | 0.2 | 0.0 | 3.7 | 1.5 | -0.5 |
| Sagar | 0.0 | 0.2 | 0.0 | 1.5 | 2.4 | 0.2 |
| Damoh | 0.5 | 0.2 | -0.1 | 4.3 | 3.4 | -0.2 |
| Satna | 0.2 | 0.1 | 0.0 | 3.0 | 1.4 | -0.3 |
| Rewa | 0.1 | 0.2 | 0.0 | 2.3 | 4.5 | 0.4 |
| Umaria | 0.1 | 0.3 | 0.0 | 4.4 | 5.6 | 0.2 |
| Neemuch | 0.5 | 0.2 | -0.1 | 4.5 | 2.5 | -0.4 |
| Mandsaur | 0.0 | 0.2 | 0.0 | 2.2 | 1.6 | -0.1 |
| Ratlam | 0.1 | 0.2 | 0.0 | 3.2 | 4.4 | 0.2 |
| Ujjain | 0.0 | 0.2 | 0.0 | 3.5 | 3.3 | 0.0 |
| Dewas | 0.0 | 0.2 | 0.0 | 1.5 | 1.4 | 0.0 |
| Dhar | 0.5 | 0.4 | 0.0 | 5.6 | 2.3 | -0.7 |
| Indore | 0.1 | 0.4 | 0.1 | 1.5 | 3.6 | 0.4 |
| Khargone (West Nimar) | 0.2 | 0.5 | 0.1 | 4.6 | 4.6 | 0.0 |
| Barwani | 0.3 | 0.5 | 0.0 | 3.5 | 2.8 | -0.1 |
| Rajgarh | 0.1 | 0.2 | 0.0 | 5.9 | 1.6 | -0.9 |
| Vidisha | 2.7 | 0.4 | -0.4 | 3.9 | 0.4 | -0.7 |
| Bhopal | 0.1 | 0.3 | 0.0 | 2.3 | 1.1 | -0.2 |
| Sehore | 1.5 | 0.1 | -0.3 | 5.7 | 2.5 | -0.6 |
| Raisen | 0.1 | 0.2 | 0.0 | 2.1 | 0.6 | -0.3 |
| Betul | 0.6 | 1.1 | 0.1 | 3.4 | 1.8 | -0.3 |
| Harda | 0.4 | 0.4 | 0.0 | 2.8 | 2.5 | -0.1 |
| Hoshangabad | 0.1 | 0.2 | 0.0 | 1.4 | 0.3 | -0.2 |
| Katni | 0.1 | 0.6 | 0.1 | 1.3 | 3.8 | 0.5 |
| Jabalpur | 0.1 | 0.5 | 0.1 | 4.3 | 4.3 | 0.0 |
| Narsimhapur | 0.0 | 0.1 | 0.0 | 4.4 | 3.0 | -0.3 |
| Dindori | 0.4 | 0.2 | 0.0 | 8.8 | 5.6 | -0.6 |
| Mandla | 0.1 | 0.2 | 0.0 | 7.5 | 2.9 | -0.9 |
| Chhindwara | 0.2 | 0.3 | 0.0 | 2.1 | 0.5 | -0.3 |
| Seoni | 2.1 | 3.5 | 0.3 | 1.3 | 1.0 | -0.1 |
| Balaghat | 6.2 | 6.5 | 0.1 | 2.2 | 0.5 | -0.3 |
| Guna | 0.6 | 0.3 | -0.1 | 6.2 | 8.1 | 0.4 |
| Ashoknagar | 1.3 | 0.0 | -0.2 | 0.2 | 0.1 | 0.0 |
| Shahdol | 0.0 | 0.1 | 0.0 | 0.3 | 0.1 | 0.0 |
| Anuppur | 0.2 | 0.3 | 0.0 | 1.6 | 0.4 | -0.2 |
| Singrauli | 3.3 | 0.1 | -0.7 | 0.3 | 0.1 | 0.0 |
| Jhabua | 2.9 | 0.3 | -0.5 | 0.3 | 0.1 | 0.0 |
| Alirajpur | 1.2 | 0.2 | -0.2 | 0.9 | 0.4 | -0.1 |
| Khandwa (East Nimar) | 0.4 | 0.1 | -0.1 | 0.2 | 0.1 | 0.0 |
| Burhanpur | 1.9 | 0.1 | -0.4 | 0.4 | 0.3 | 0.0 |
| Agar Malwa | 0.1 | 0.1 | 0.0 | 0.9 | 0.2 | -0.1 |
| Shajapur | 0.0 | 0.0 | 0.0 | 0.3 | 0.1 | 0.0 |
| Sidhi | 0.6 | 0.1 | -0.1 | 1.1 | 0.1 | -0.2 |
| Kachchh | 0.2 | 0.5 | 0.1 | 1.6 | 1.1 | -0.1 |
| Banas Kantha | 0.0 | 0.0 | 0.0 | 0.4 | 0.2 | 0.0 |
| Patan | 0.0 | 0.1 | 0.0 | 1.5 | 0.2 | -0.3 |
| Mahesana | 0.0 | 0.1 | 0.0 | 2.6 | 1.5 | -0.2 |
| Gandhinagar | 0.0 | 0.0 | 0.0 | 2.2 | 0.9 | -0.2 |
| Porbandar | 0.0 | 0.1 | 0.0 | 1.4 | 0.7 | -0.1 |
| Anand | 1.0 | 0.1 | -0.2 | 2.1 | 1.6 | -0.1 |
| Dohad | 0.5 | 0.5 | 0.0 | 2.2 | 0.8 | -0.3 |
| Narmada | 0.0 | 0.0 | 0.0 | 1.3 | 0.9 | -0.1 |
| Bharuch | 0.1 | 0.1 | 0.0 | 1.2 | 0.7 | -0.1 |
| The Dangs | 0.1 | 0.2 | 0.0 | 1.0 | 0.8 | 0.0 |
| Navsari | 0.0 | 0.1 | 0.0 | 2.7 | 0.5 | -0.4 |
| Valsad | 0.0 | 0.0 | 0.0 | 2.5 | 1.2 | -0.2 |
| Tapi | 0.0 | 0.3 | 0.1 | 3.0 | 0.6 | -0.5 |
| Ahmadabad | 0.3 | 0.2 | 0.0 | 4.1 | 3.4 | -0.1 |
| Aravali | 0.1 | 0.1 | 0.0 | 4.7 | 1.7 | -0.6 |
| Bhavnagar | 0.2 | 0.2 | 0.0 | 2.9 | 1.5 | -0.3 |
| Botad | 0.1 | 0.4 | 0.1 | 3.4 | 1.6 | -0.4 |
| Chhota Udaipur | 0.0 | 0.2 | 0.0 | 0.8 | 0.3 | -0.1 |
| Devbhoomi Dwarka | 0.3 | 0.3 | 0.0 | 0.4 | 0.1 | -0.1 |
| Gir Somnath | 0.0 | 0.1 | 0.0 | 1.2 | 0.4 | -0.2 |
| Jamnagar | 0.4 | 0.2 | 0.0 | 0.3 | 0.2 | 0.0 |
| Junagadh | 0.1 | 0.0 | 0.0 | 0.7 | 0.2 | -0.1 |
| Kheda | 0.3 | 0.0 | -0.1 | 0.4 | 0.2 | 0.0 |
| Mahisagar | 0.1 | 0.0 | 0.0 | 0.6 | 0.1 | -0.1 |
| Morbi | 0.3 | 0.1 | 0.0 | 1.8 | 0.6 | -0.2 |
| Panch Mahals | 0.0 | 0.1 | 0.0 | 2.7 | 1.3 | -0.3 |
| Rajkot | 0.0 | 0.1 | 0.0 | 4.8 | 1.4 | -0.7 |
| Sabar Kantha | 0.0 | 0.1 | 0.0 | 1.1 | 1.0 | 0.0 |
| Surendranagar | 0.1 | 0.1 | 0.0 | 0.1 | 0.1 | 0.0 |
| Vadodara | 0.2 | 0.2 | 0.0 | 0.7 | 0.1 | -0.1 |
| Surat | 28.1 | 14.0 | -2.8 | 15.0 | 17.8 | 0.6 |
| Amreli | 10.4 | 7.2 | -0.6 | 9.5 | 2.6 | -1.4 |
| Daman | 7.9 | 20.5 | 2.5 | 6.1 | 6.2 | 0.0 |
| Dadra & Nagar Haveli | 28.0 | 18.0 | -2.0 | 27.7 | 8.7 | -3.8 |
| Diu | 41.6 | 16.3 | -5.1 | 18.7 | 4.0 | -2.9 |
| Nandurbar | 31.5 | 24.8 | -1.3 | 29.1 | 10.1 | -3.8 |
| Dhule | 32.5 | 19.0 | -2.7 | 10.6 | 6.7 | -0.8 |
| Jalgaon | 47.0 | 10.6 | -7.3 | 23.7 | 3.2 | -4.1 |
| Buldana | 26.1 | 24.9 | -0.2 | 8.1 | 11.2 | 0.6 |
| Akola | 10.8 | 9.8 | -0.2 | 15.6 | 16.4 | 0.2 |
| Washim | 12.1 | 13.5 | 0.3 | 27.2 | 14.1 | -2.6 |
| Amravati | 0.1 | 0.2 | 0.0 | 21.1 | 6.8 | -2.9 |
| Wardha | 0.5 | 1.1 | 0.1 | 22.7 | 7.6 | -3.0 |
| Nagpur | 5.8 | 2.7 | -0.6 | 17.5 | 10.7 | -1.4 |
| Bhandara | 14.1 | 17.8 | 0.7 | 14.6 | 7.6 | -1.4 |
| Gondiya | 31.1 | 30.3 | -0.2 | 13.1 | 11.8 | -0.3 |
| Gadchiroli | 0.4 | 2.1 | 0.3 | 12.6 | 9.6 | -0.6 |
| Chandrapur | 13.1 | 10.8 | -0.5 | 19.5 | 15.3 | -0.8 |
| Yavatmal | 6.8 | 3.1 | -0.7 | 15.1 | 8.5 | -1.3 |
| Nanded | 0.1 | 0.2 | 0.0 | 12.1 | 13.1 | 0.2 |
| Hingoli | 53.8 | 18.4 | -7.1 | 25.2 | 5.2 | -4.0 |
| Parbhani | 2.2 | 0.8 | -0.3 | 27.7 | 3.2 | -4.9 |
| Jalna | 0.9 | 0.7 | 0.0 | 16.9 | 3.7 | -2.6 |
| Nashik | 0.1 | 0.2 | 0.0 | 19.6 | 12.2 | -1.5 |
| Mumbai Suburban | 18.0 | 13.7 | -0.9 | 47.6 | 30.0 | -3.5 |
| Mumbai | 0.9 | 0.4 | -0.1 | 17.4 | 3.2 | -2.8 |
| Raigarh | 0.8 | 1.6 | 0.2 | 12.4 | 4.9 | -1.5 |
| Pune | 32.1 | 16.8 | -3.1 | 58.0 | 24.6 | -6.7 |
| Ahmadnagar | 0.7 | 0.3 | -0.1 | 12.6 | 1.2 | -2.3 |
| Bid | 0.9 | 0.7 | 0.0 | 37.4 | 4.9 | -6.5 |
| Latur | 20.0 | 12.0 | -1.6 | 43.0 | 17.6 | -5.1 |
| Osmanabad | 0.9 | 0.5 | -0.1 | 13.6 | 7.0 | -1.3 |
| Solapur | 0.7 | 0.5 | 0.0 | 17.1 | 2.9 | -2.9 |
| Satara | 21.4 | 25.5 | 0.8 | 53.0 | 31.6 | -4.3 |
| Ratnagiri | 8.9 | 7.4 | -0.3 | 22.6 | 14.5 | -1.6 |
| Sindhudurg | 0.4 | 0.1 | -0.1 | 0.9 | 1.9 | 0.2 |
| Kolhapur | 0.2 | 0.3 | 0.0 | 1.2 | 0.6 | -0.1 |
| Sangli | 0.1 | 0.0 | 0.0 | 1.4 | 0.6 | -0.2 |
| Palghar | 0.2 | 0.3 | 0.0 | 1.8 | 2.3 | 0.1 |
| Thane | 0.1 | 0.1 | 0.0 | 1.2 | 0.9 | -0.1 |
| Aurangabad | 1.0 | 0.1 | -0.2 | 1.8 | 1.3 | -0.1 |
| Bagalkot | 0.1 | 0.2 | 0.0 | 1.0 | 0.4 | -0.1 |
| Bijapur | 1.0 | 0.3 | -0.1 | 1.0 | 1.5 | 0.1 |
| Bidar | 0.6 | 0.6 | 0.0 | 1.2 | 0.5 | -0.1 |
| Raichur | 0.2 | 0.4 | 0.0 | 1.2 | 0.9 | -0.1 |
| Koppal | 0.3 | 0.3 | 0.0 | 0.9 | 1.2 | 0.1 |
| Gadag | 0.0 | 0.1 | 0.0 | 3.6 | 3.4 | 0.0 |
| Dharwad | 0.0 | 0.1 | 0.0 | 8.6 | 4.3 | -0.9 |
| Uttara Kannada | 0.0 | 0.0 | 0.0 | 5.0 | 5.4 | 0.1 |
| Haveri | 0.0 | 0.3 | 0.1 | 3.0 | 1.1 | -0.4 |
| Bellary | 1.0 | 0.5 | -0.1 | 9.7 | 3.5 | -1.2 |
| Chitradurga | 0.2 | 0.2 | 0.0 | 16.1 | 21.2 | 1.0 |
| Davanagere | 0.0 | 0.2 | 0.0 | 12.2 | 13.4 | 0.2 |
| Shimoga | 0.2 | 0.1 | 0.0 | 7.5 | 4.2 | -0.7 |
| Chikmagalur | 0.0 | 0.1 | 0.0 | 6.3 | 11.5 | 1.0 |
| Tumkur | 0.1 | 0.2 | 0.0 | 9.2 | 9.8 | 0.1 |
| Bangalore | 0.1 | 0.1 | 0.0 | 7.0 | 3.4 | -0.7 |
| Mandya | 0.0 | 0.1 | 0.0 | 3.5 | 3.6 | 0.0 |
| Hassan | 0.3 | 0.1 | 0.0 | 5.1 | 4.7 | -0.1 |
| Dakshina Kannada | 0.1 | 0.1 | 0.0 | 2.6 | 3.1 | 0.1 |
| Kodagu | 0.0 | 0.2 | 0.0 | 4.9 | 2.9 | -0.4 |
| Mysore | 0.0 | 0.1 | 0.0 | 4.5 | 1.7 | -0.6 |
| Chamarajanagar | 0.1 | 0.2 | 0.0 | 11.2 | 3.1 | -1.6 |
| Gulbarga | 0.0 | 0.1 | 0.0 | 0.7 | 0.1 | -0.1 |
| Yadgir | 0.0 | 0.1 | 0.0 | 0.6 | 0.1 | -0.1 |
| Kolar | 0.0 | 0.2 | 0.0 | 1.7 | 0.7 | -0.2 |
| Chikkaballapura | 0.1 | 0.2 | 0.0 | 2.0 | 1.1 | -0.2 |
| Bangalore Rural | 0.2 | 0.8 | 0.1 | 4.9 | 2.8 | -0.4 |
| Ramanagara | 0.1 | 0.2 | 0.0 | 7.8 | 3.0 | -0.9 |
| Udupi | 2.2 | 0.5 | -0.3 | 9.2 | 8.6 | -0.1 |
| North Goa | 0.3 | 2.3 | 0.4 | 51.1 | 42.3 | -1.8 |
| South Goa | 2.2 | 1.5 | -0.1 | 8.0 | 3.5 | -0.9 |
| Lakshadweep | 1.0 | 0.6 | -0.1 | 16.6 | 12.7 | -0.8 |
| Wayanad | 0.1 | 0.8 | 0.1 | 44.8 | 45.6 | 0.2 |
| Kozhikode | 6.3 | 1.6 | -0.9 | 21.6 | 21.3 | -0.1 |
| Malappuram | 0.3 | 0.8 | 0.1 | 49.4 | 37.8 | -2.3 |
| Palakkad | 0.3 | 0.9 | 0.1 | 46.0 | 44.8 | -0.2 |
| Thrissur | 0.0 | 0.1 | 0.0 | 0.1 | 0.0 | 0.0 |
| Ernakulam | 0.0 | 0.1 | 0.0 | 0.1 | 0.0 | 0.0 |
| Idukki | 0.0 | 0.4 | 0.1 | 0.1 | 0.0 | 0.0 |
| Kottayam | 0.0 | 0.1 | 0.0 | 0.1 | 0.0 | 0.0 |
| Pathanamthitta | 0.4 | 0.8 | 0.1 | 5.7 | 5.4 | -0.1 |
| Kollam | 1.7 | 1.0 | -0.1 | 4.0 | 1.1 | -0.6 |
| Thiruvananthapuram | 0.9 | 0.2 | -0.1 | 1.5 | 0.3 | -0.2 |
| Kasaragod | 10.3 | 1.5 | -1.8 | 8.9 | 5.1 | -0.8 |
| Kannur | 12.4 | 6.7 | -1.1 | 1.2 | 0.7 | -0.1 |
| Alappuzha | 7.7 | 1.2 | -1.3 | 3.9 | 0.7 | -0.6 |
| Chennai | 2.9 | 1.9 | -0.2 | 3.5 | 3.5 | 0.0 |
| Kancheepuram | 29.2 | 10.4 | -3.8 | 3.6 | 1.4 | -0.5 |
| Vellore | 4.9 | 2.4 | -0.5 | 3.6 | 2.3 | -0.3 |
| Tiruvannamalai | 3.4 | 0.3 | -0.6 | 1.8 | 0.3 | -0.3 |
| Viluppuram | 0.9 | 1.5 | 0.1 | 4.3 | 5.2 | 0.2 |
| Salem | 10.4 | 7.2 | -0.6 | 1.2 | 0.5 | -0.1 |
| Namakkal | 18.5 | 3.0 | -3.1 | 3.3 | 1.5 | -0.4 |
| Erode | 0.9 | 0.4 | -0.1 | 3.4 | 1.1 | -0.5 |
| The Nilgiris | 6.7 | 16.6 | 2.0 | 2.3 | 1.4 | -0.2 |
| Dindigul | 3.4 | 1.0 | -0.5 | 1.4 | 1.0 | -0.1 |
| Karur | 24.2 | 6.3 | -3.6 | 2.9 | 0.9 | -0.4 |
| Tiruchirappalli | 11.8 | 5.0 | -1.4 | 1.0 | 0.3 | -0.1 |
| Perambalur | 1.2 | 1.3 | 0.0 | 6.3 | 3.6 | -0.5 |
| Ariyalur | 9.8 | 4.0 | -1.2 | 3.5 | 2.7 | -0.2 |
| Cuddalore | 2.0 | 1.9 | 0.0 | 2.9 | 1.2 | -0.3 |
| Nagapattinam | 14.5 | 3.6 | -2.2 | 4.1 | 2.9 | -0.3 |
| Thiruvarur | 16.2 | 2.8 | -2.7 | 2.2 | 0.8 | -0.3 |
| Thanjavur | 9.4 | 5.9 | -0.7 | 2.1 | 1.3 | -0.2 |
| Pudukkottai | 25.7 | 4.9 | -4.2 | 3.4 | 1.3 | -0.4 |
| Sivaganga | 21.4 | 3.3 | -3.6 | 0.9 | 0.3 | -0.1 |
| Madurai | 27.6 | 10.8 | -3.4 | 2.7 | 2.8 | 0.0 |
| Theni | 12.8 | 3.6 | -1.8 | 3.3 | 2.0 | -0.3 |
| Virudhunagar | 4.7 | 2.3 | -0.5 | 0.9 | 0.4 | -0.1 |
| Thoothukkudi | 4.7 | 1.3 | -0.7 | 1.1 | 0.3 | -0.2 |
| Tirunelveli | 20.7 | 5.7 | -3.0 | 1.0 | 0.8 | -0.1 |
| Kanniyakumari | 8.0 | 7.0 | -0.2 | 51.1 | 42.0 | -1.8 |
| Dharmapuri | 7.6 | 8.6 | 0.2 | 35.5 | 38.3 | 0.5 |
| Krishnagiri | 1.6 | 5.8 | 0.8 | 47.1 | 36.7 | -2.1 |
| Coimbatore | 4.5 | 3.5 | -0.2 | 42.6 | 32.9 | -1.9 |
| Tiruppur | 1.6 | 2.7 | 0.2 | 52.5 | 37.5 | -3.0 |
| Thiruvallur | 1.2 | 2.8 | 0.3 | 37.0 | 37.5 | 0.1 |
| Ramanathapuram | 0.1 | 0.1 | 0.0 | 6.9 | 3.5 | -0.7 |
| South Andaman | 0.0 | 0.2 | 0.0 | 5.4 | 1.1 | -0.9 |
| Nicobars | 0.0 | 0.0 | 0.0 | 2.2 | 0.4 | -0.4 |
| North & Middle Andaman | 0.0 | 0.0 | 0.0 | 2.3 | 0.6 | -0.3 |
| Adilabad | 0.0 | 0.1 | 0.0 | 2.9 | 1.3 | -0.3 |
| Bhadradri Kothagudem | 0.0 | 0.1 | 0.0 | 3.5 | 0.3 | -0.6 |
| Hyderabad | 0.1 | 0.2 | 0.0 | 10.9 | 4.3 | -1.3 |
| Jagtial | 0.0 | 0.1 | 0.0 | 3.0 | 1.0 | -0.4 |
| Jangaon | 0.0 | 0.1 | 0.0 | 4.9 | 0.3 | -0.9 |
| Jayashankar Bhupalapally | 0.0 | 0.1 | 0.0 | 4.8 | 3.7 | -0.2 |
| Jogulambha Gadwal | 0.1 | 0.3 | 0.0 | 16.1 | 9.5 | -1.3 |
| Kamareddy | 0.2 | 0.4 | 0.0 | 7.4 | 3.2 | -0.8 |
| Karimnagar | 0.2 | 0.1 | 0.0 | 0.5 | 0.6 | 0.0 |
| Khammam | 0.4 | 0.2 | 0.0 | 4.4 | 2.9 | -0.3 |
| Kumuram Bheem (Asifabad) | 0.0 | 0.2 | 0.0 | 4.3 | 2.2 | -0.4 |
| Mahabubabad | 11.9 | 4.3 | -1.5 | 23.3 | 7.5 | -3.2 |
| Mahbubnagar | 0.0 | 0.4 | 0.1 | 2.2 | 2.0 | 0.0 |
| Mancherial | 0.1 | 0.1 | 0.0 | 19.3 | 5.9 | -2.7 |
| Medak | 4.0 | 6.2 | 0.4 | 36.3 | 42.4 | 1.2 |
| Medchal-Malkajgiri | 0.6 | 0.4 | 0.0 | 2.8 | 1.0 | -0.4 |
| Nagarkurnool | 0.1 | 0.1 | 0.0 | 0.6 | 0.4 | 0.0 |
| Nalgonda | 0.0 | 0.1 | 0.0 | 15.5 | 3.2 | -2.5 |
| Nirmal | 1.1 | 0.1 | -0.2 | 0.3 | 0.1 | -0.1 |
| Nizamabad | 0.9 | 0.1 | -0.2 | 0.3 | 0.1 | 0.0 |
| Peddapalle | 1.6 | 0.5 | -0.2 | 67.6 | 45.9 | -4.3 |
| Rajanna Sircilla | 0.9 | 1.1 | 0.0 | 19.4 | 9.7 | -1.9 |
| Ranga Reddy | 0.0 | 0.2 | 0.0 | 0.7 | 0.2 | -0.1 |
| Sangareddy | 8.8 | 18.1 | 1.8 | 59.3 | 48.8 | -2.1 |
| Siddipet | 0.6 | 0.0 | -0.1 | 1.6 | 0.3 | -0.3 |
| Suryapet | 0.1 | 0.3 | 0.0 | 3.6 | 1.3 | -0.5 |
| Vikarabad | 0.1 | 0.3 | 0.0 | 13.6 | 9.8 | -0.8 |
| Wanaparthy | 1.7 | 2.7 | 0.2 | 30.5 | 22.8 | -1.5 |
| Warangal Rural | 3.6 | 0.7 | -0.6 | 0.8 | 0.3 | -0.1 |
| Warangal Urban |  |  |  |  |  |  |
| Yadadri Bhuvanagiri | 0.0 | 0.4 | 0.1 | 1.7 | 0.1 | -0.3 |
| Leh | 0.0 | 0.1 | 0.0 | 2.1 | 1.8 | -0.1 |
| Kargil | 0.0 | 0.1 | 0.0 | 0.5 | 0.2 | -0.1 |
| Aurangabad | 4.2 | 0.3 | -0.8 | 0.2 | 0.1 | 0.0 |

Note: AAC (% points per annum) = (P_t_ −P_t-n_ )∗(1/n ).
